# Modular synthesis of unsymmetrical [1]benzothieno[3,2-*b*][1]benzothiophene molecular semiconductors for organic transistors†

**DOI:** 10.1039/d1sc05070b

**Published:** 2021-11-29

**Authors:** Masanori Tayu, Aiman Rahmanudin, Gregory J. P. Perry, Raja U. Khan, Daniel J. Tate, Raymundo Marcial-Hernandez, Yuan Shen, Ingo Dierking, Yurachat Janpatompong, Suphaluk Aphichatpanichakul, Adibah Zamhuri, Inigo Victoria-Yrezabal, Michael L. Turner, David J. Procter

**Affiliations:** Department of Chemistry, University of Manchester Oxford Road Manchester M13 9PL UK michael.turner@manchester.ac.uk david.j.procter@manchester.ac.uk; Department of Physics & Astronomy, University of Manchester Oxford Road Manchester M13 9PL UK

## Abstract

A modular approach to underexplored, unsymmetrical [1]benzothieno[3,2-*b*][1]benzothiophene (BTBT) scaffolds delivers a library of BTBT materials from readily available coupling partners by combining a transition-metal free Pummerer CH–CH-type cross-coupling and a Newman–Kwart reaction. This effective approach to unsymmetrical BTBT materials has allowed their properties to be studied. In particular, tuning the functional groups on the BTBT scaffold allows the solid-state assembly and molecular orbital energy levels to be modulated. Investigation of the charge transport properties of BTBT-containing small-molecule:polymer blends revealed the importance of molecular ordering during phase segregation and matching the highest occupied molecular orbital energy level with that of the semiconducting polymer binder, polyindacenodithiophene-benzothiadiazole (PIDTBT). The hole mobilities extracted from transistors fabricated using blends of PIDTBT with phenyl or methoxy functionalized unsymmetrical BTBTs were double those measured for devices fabricated using pristine PIDTBT. This study underscores the value of the synthetic methodology in providing a platform from which to study structure–property relationships in an underrepresented family of unsymmetrical BTBT molecular semiconductors.

## Abstract 2

A modular approach to underexplored, unsymmetrical [1]benzothieno[3,2-*b*][1]benzothiophene (BTBT) scaffolds, combining a transition-metal free Pummerer CH–CH-type cross-coupling and a Newman–Kwart reaction, delivers a library of BTBT materials.

## Graphical abstract

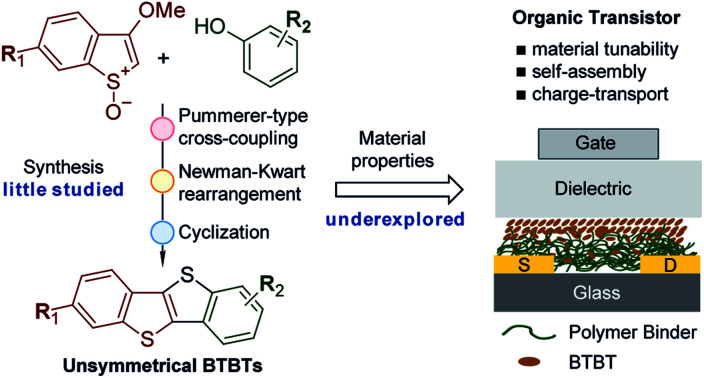

## Introduction

**Scheme 1:**
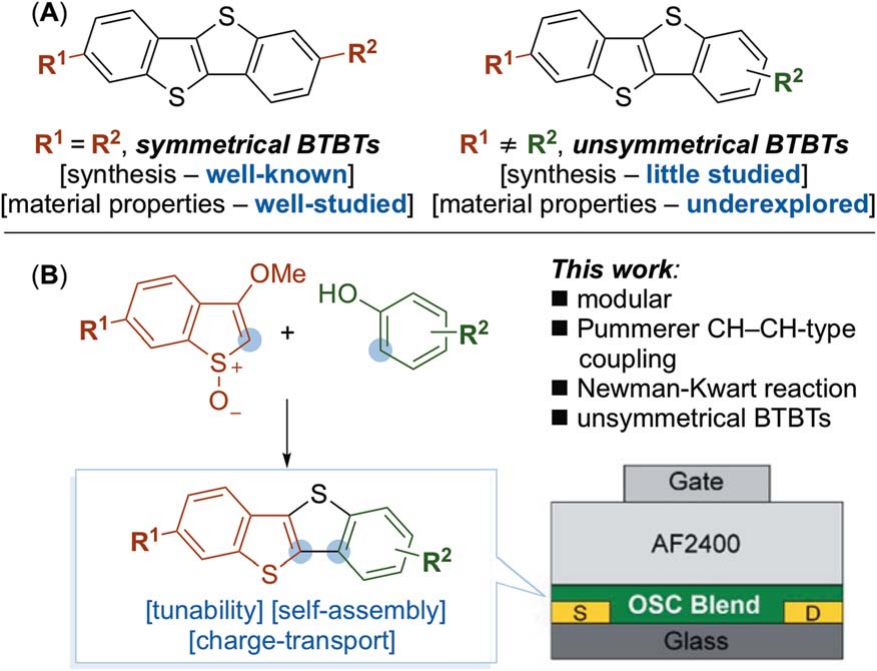
(A) Comparison of symmetrical and unsymmetrical BTBT compounds. (B) This work: the synthesis and properties of underexplored unsymmetrical BTBT compounds. OSC = organic semiconductor.

Organic semiconductors (OSCs) are essential active components in a wide range of next-generation electronic and energy devices including field-effect transistors,^1^ solar energy convertors,^2^ and chemical/bio sensors.^3^ The charge transport in OSCs is strongly governed by their molecular organization in the solid-state (*i.e.* thin-film structure) and their electronic properties. In general, these properties can be tuned through molecular design of the OSC architecture (*e.g.* side-chain engineering, conjugated backbone structure),^4^ conjugation break spacers,^5^ and/or the type of thin-film deposition technique.^6^ Among the different OSCs, conjugated [1]benzothieno[3,2-*b*][1]benzothiophene (BTBT) scaffolds have been extensively used as a platform for the construction of high-performing small-molecule semiconductors.^7^ Their tendency to pack into highly ordered structures during film formation often results in favorable charge (hole) transport in transistors.^8^ Most studies have investigated the packing behavior of symmetrical 2,7-functionalized BTBTs and the relationship between transistor properties and variations in length (odd-even effect),^9^ functional end-groups on the aliphatic side-chains, and bulky substituent groups (Scheme 1A).^10^ A prototypical example is the widely used 2,7-dioctyl-BTBT (C_8_-BTBT) which has reported some of the highest hole mobilities (*μ*_Hole_); above 10 cm^2^ V^−1^ s^−1^ from solution-processed OFETs as single-crystals, binary blends with polymer insulators (*e.g.* polystyrene),^11^ and ternary blends with a polymer semiconductor and various p-dopants.^12^

Unsymmetrical BTBT compounds have the potential to outperform symmetrical BTBTs; for example, Hanna and coworkers have reported mobilities as high as 14.7 cm^2^ V^−1^ s^−1^ for a liquid crystalline 2-decyl-7-phenyl-BTBT.^13^ Despite these promising results, the investigation of unsymmetrical BTBT derivatives lags behind that of their symmetrical counterparts (Scheme 1A).^14^ This is in part due to a lack of efficient methods for their synthesis. For example, most routes towards unsymmetrical BTBTs are limited in scope and/or use transition metals during the final step of the synthesis – leading to metallic impurities that are known to affect the performance of organic materials.^15,16^ As there are few general approaches for the synthesis of unsymmetrical BTBTs,^17^ we considered a modular synthesis, less reliant on the use of transition metals, that would grant access to a variety of compounds and allow facile exploration of their material properties (Scheme 1B).

Here we describe a versatile, modular synthesis of unsymmetrical BTBT compounds (Scheme 1B). Key to our approach is the application of a transition-metal free Pummerer CH–CH-type cross-coupling in conjunction with a Newman–Kwart rearrangement. Exploiting the new approach, we investigated the material properties of a selected series of unsymmetrical BTBT derivatives containing a decane aliphatic side chain at the C2 position (required for solubility), and a variety of electron donating and electron withdrawing groups at the C7 position. Tuning the substituent at the C7 position influenced the electronic properties and solid-state assembly of the BTBTs. The BTBT molecules were blended with a polymer semiconductor binder, PIDTBT to aid processing into thin-films in small-molecule:polymer (S-M:polymer) blend transistors. Preliminary investigations into the charge transport properties highlighted the importance of molecular ordering of the BTBT molecules during phase segregation and matching of the highest occupied molecular orbital (HOMO) level with that of the polymer semiconductor binder. Crucially, hole mobilities extracted from devices fabricated using phenyl and methoxy functionalized unsymmetrical BTBT molecules blended with PIDTBT were higher than those extracted from comparable devices fabricated using pristine PIDTBT or the symmetrical C_8_-BTBT in a S-M:polymer blend transistor.

## Results and discussion

**Scheme 2:**
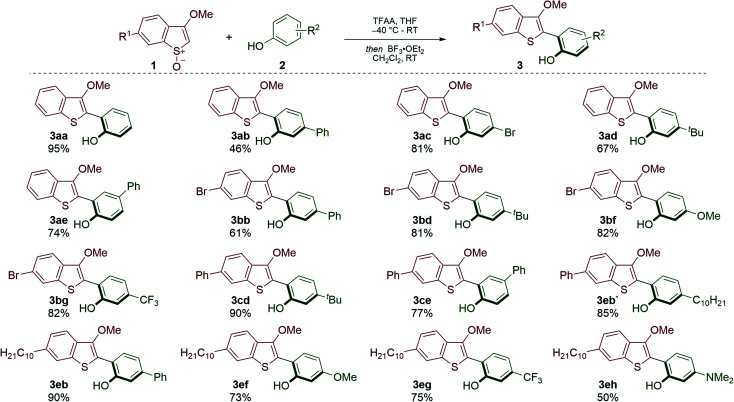
Pummerer CH–CH-type couplings of benzothiophene *S*-oxides and phenols. Reaction conditions: 1 (1.0 equiv.), 2 (1.5 equiv.), TFAA (1.5 equiv.), THF (0.1 M), −40 °C to RT, then BF_3_·OEt_2_ (0.2 equiv.), CH_2_Cl_2_, 0.1 M, RT.

We have recently developed Pummerer CH–CH-type cross-coupling processes^18^ that exploit activation of the benzothiophene partner by convenient *S*-oxidation and deliver functionalized benzothiophenes.^19^ We reasoned that this new metal-free cross-coupling process could provide modular access to underexplored BTBT materials. Our investigation began with the metal-free coupling of benzothiophenes, activated as their *S*-oxides, 1 with phenols 2. Accordingly, cross-coupled products 3 bearing various functionalities were efficiently prepared (Scheme 2). Notably, bromo-substituents were tolerated on the biaryl scaffold (3ac, 3bb–3bg), thus allowing further transformations. Electron-withdrawing (3bg, 3eg) and electron-donating (3bf, 3ef, 3eh) substituents were compatible with the process, thus providing an opportunity to tune the electronic properties of the target BTBT materials.

**Scheme 3:**
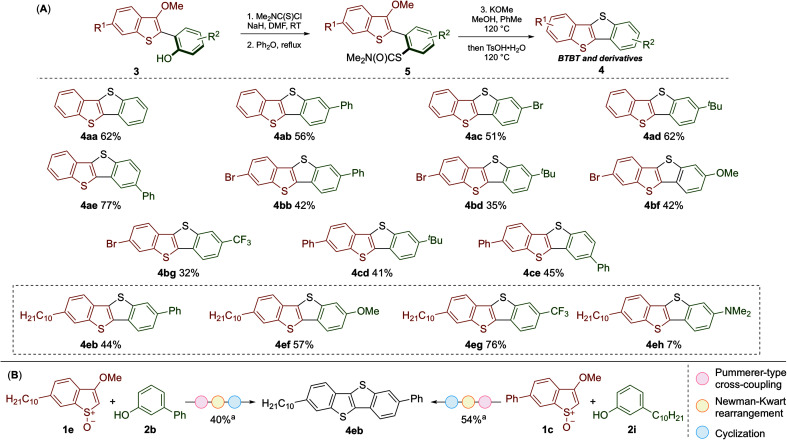
(A) Scope of the metal-free synthesis of BTBT materials by Newman–Kwart reaction of coupling products 3, followed by cyclization. Yields are of the overall process from 3 to 4. (B) Parallel synthesis of 4eb from 1e and 2b, and 1c and 2i. ^*a*^Isolated yield for the overall process from 1 and 2. Reaction conditions for 3 to 5: 3 (1.0 equiv.), Me_2_NC(S)Cl (2.0 equiv.), NaH (3.0 equiv.), DMF (0.1 M), RT·Ph_2_O (0.1 M), reflux. Reaction conditions for 5 to 4: 5 (1 equiv.), KOMe (2.0 equiv.), MeOH/PhMe (1 : 1, 0.05 M), 120 °C, then TsOH·H_2_O (8.0 equiv.), 120 °C.

The conversion of the coupling products 3 into the desired BTBT products 4 involved a Newmann–Kwart reaction, to give intermediates 5,^20^ followed by cyclization (Scheme 3A). While this method can deliver symmetrical products (*e.g.*4aa), we focused on preparing more elusive unsymmetrical BTBT materials. The position of the substituents around the BTBT core was easily altered by the choice of phenol coupling partner 2; for example, regioisomers 4ab and 4ae were obtained using the same synthetic route but selecting either *meta*- or *para*-substituted phenol partners. As a variety of substituted benzothiophene and phenol partners are commercially available, this flexibility will prove useful when planning the synthesis of target unsymmetrical BTBT materials. The adaptability of our approach was also demonstrated in the synthesis of 4eb (Scheme 3B). This BTBT material was prepared by parallel routes from either benzothiophene *S*-oxide 1e and phenol 2b, or 1c and 2i (*via*3eb or 3eb′ respectively, see Scheme 2). We were particularly attracted to the synthesis of BTBT 4eb as it has displayed high charge mobility (14.7 cm^2^ V^−1^ s^−1^).^13^ Therefore, we prepared a range of related derivatives (4ef–4eh) to investigate how substituents affect the properties of these unsymmetrical materials.

**Fig. 1:**
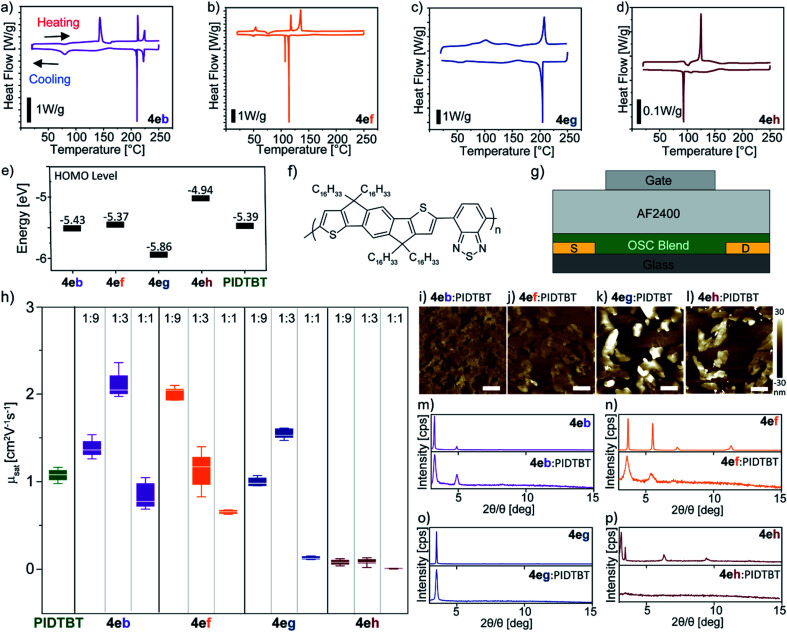
Thermal and electrochemical properties of the BTBT molecules: DSC thermograms taken from 2^nd^ heating and cooling cycle: (a) 4eb (R^2^ = Ph); (b) 4ef (R^2^ = OMe); (c) 4eg (R^2^ = CF_3_); (d) 4eh (R^2^ = NMe_2_) at 5 °C min^−1^; (e) energy level diagram indicating HOMO levels including PIDTBT; (f) molecular structure of the polymer semiconductor PIDTBT; (g) top-gated OFET device structure with an aluminium gate electrode, AF2400 as the gate dielectric, and gold source-drain electrodes with a channel width and length of 1000 mm and 60 mm, respectively; (h) box and whiskers plot comparing the saturated mobility (*μ*_sat_) from measured devices containing pristine PIDTBT and blends of 4e(b,f,g,h):PIDTBT at a ratio of 1 : 9, 1 : 3, 1 : 1; atomic force microscopy (AFM) topography images of the 4e(b,f,g,h):PIDTBT blend films at 1 : 3 ratio processed from tetralin:chlorobenzene (i–l). The white scale bar indicates a length of 2 μm; grazing incidence X-ray diffraction of thin films based on individual BTBT derivatives (top) and as a blend with PIDTBT (bottom): (m) 4eb; (n) 4ef; (o) 4eg; (p) 4eh.

With a range of new BTBT derivatives in hand, we firstly examined the thermal properties of the selected BTBT derivatives (4eb, 4ef–4eh) using differential scanning calorimetry (DSC). As previously reported,^13^4eb exhibited liquid crystal phase transitions of SmE at 143 °C and 79 °C, and SmA at 212 °C and 210 °C during the heating and cooling cycle, respectively (Fig. 1a). The typical smooth fan-shaped texture of the fluid SmA phase and the striated fan-like one of the soft crystal SmE phase were confirmed by polarized microscopy (POM) (ESI Fig. S1†). In contrast, 4ef, 4eg and 4eh showed typical behaviour of crystalline material in the DSC curves and this was supported by the POM images. BTBT 4ef showed multiple phase transitions with a large sharp transition enthalpy at 114 °C upon cooling from an isotropic phase, with a second smaller peak at 107 °C; this is most likely a 2nd polymorphic phase (Fig. 1b). On the other hand, the DSC curves for 4eg and 4eh only show sharp melting and crystallization peaks upon heating and cooling (Fig. 1c and d). Next, the energy levels of the selected unsymmetrical BTBT derivatives (4eb, 4ef–4eh) were investigated using cyclic voltammetry; Fig. 1e shows the respective energy levels. The highest occupied molecular orbital (HOMO) levels were estimated from the onset of the oxidation peak (*E*^oxd^_onset_) in the cyclic voltammogram of the BTBT molecules (Fig. S2†). The HOMO level obtained for 4eb is similar to reported values; *E*_HOMO_ = −5.43 eV.^13^ It is evident that the type of functional group at the C7 position influences the HOMO level of the unsymmetrical BTBT scaffold. The electron donating ability of the –NMe_2_ and –OMe groups resulted in higher HOMO levels where *E*_HOMO_ = −5.37 and −4.94 eV for 4ef and 4eh, respectively, while the electron withdrawing group (–CF_3_) on 4eg decreases its HOMO level to *E*_HOMO_ = −5.86 eV.^21^ The trend observed is qualitatively confirmed by values calculated using density functional theory (Fig. S2†).

Initial attempts at reproducing transistor performance using adapted reported conditions for 4eb resulted in low *μ*_sat_ = 1.59 cm^2^ V^−1^ s^−1^, and we were unable to reproduce the reported high mobilities of 14.7 cm^2^ V^−1^ s^−1^ on bottom-gate/top-contact transistors (Si/SiO_2_/4eb/Au).^13^ Fig. S3† shows the relevant transistor characteristics. Furthermore, solution processing the remaining BTBT derivatives resulted in largely non-uniform films (Fig. S4†) that led to inconsistent transistor behaviour. To improve the film forming properties of the unsymmetrical BTBT derivatives, and to investigate their charge-carrier properties, a polymer semiconductor PIDTBT was used as a binder (Fig. 1f). This approach is based on recent reports of S-M:polymer blend transistors of symmetrical C_8_-BTBT:PIDTBT that exploit the highly ordered nature of small-molecules for efficient charge transport and the superior film forming properties of the polymer binder.^12,22^ Top-gated OFETs using poly[4,5-difluoro-2,2bis(trifluoromethyl)-1,3-dioxole-*co*-tetrafluoroethylene] (Teflon AF2400) as a dielectric were fabricated (Fig. 1g) to assess the charge transport properties of OSC blends of 4e(b,f,g,h):PIDTBT. We performed preliminary optimization of the blend ratio at 1 : 9, 1 : 3, and 1 : 1. As controls, transistors based on pristine PIDTBT and its blend with the prototypical symmetrical C_8_-BTBT were fabricated, and their performance evaluated. The BTBT molecules and PIDTBT were dissolved in tetralin and chlorobenzene separately before mixing into a blend solution at the respective ratios for thin-film processing. The calculated mobility values *μ*_sat_ for all devices were taken in the saturation regime at *V*_GS_ = −60 V from the corresponding mobility dependence on applied gate voltage plot extracted from the transfer and output characteristics (Fig. S5–S10†). The overall transistor parameters (*V*_th_ and *I*_on_/*I*_off_) are summarized in Table S1 (see the ESI† for further details of device fabrication).

A summary of the *μ*_sat_ values extracted from devices containing pristine PIDTBT and 4e(b,f,g,h):PIDTBT are shown in Fig. 1h, and the mobility values for C_8_-BTBT:PIDTBT are highlighted in Table S1.† In general, all devices for blends at higher amounts of the unsymmetrical and symmetrical BTBT molecules – *i.e.* 1 : 1 ratio – had the lowest mobility (<0.8 cm^2^ V^−1^ s^−1^) (Table S1†). A similar *μ*_sat_ trend in the blend ratio was also reported in earlier work for C_8_-BTBT:PIDTBT.^12*e*^ Amongst the unsymmetrical BTBT molecules, blends using 4eh gave the lowest mobilities (<0.08 cm^2^ V^−1^ s^−1^) across all ratios. On the other hand, blends with 4eb and 4ef at a ratio of 1 : 3 and 1 : 9 achieved the highest mobility of 1.89 ± 0.3 cm^2^ V^−1^ s^−1^ and 1.87 ± 0.2 cm^2^ V^−1^ s^−1^, respectively. Devices from pristine PIDTBT obtained mobilities of 1.02 ± 0.1 cm^2^ V^−1^ s^−1^ which indicate that any improvements are due to the presence of unsymmetrical BTBT molecules in the OSC blends. Furthermore, the mobility of the best performing unsymmetrical BTBT blends were higher than comparable devices containing the symmetrical C_8_-BTBT which obtained a mobility of 1.19 ± 0.3 cm^2^ V^−1^ s^−1^ at a 1 : 3 blend ratio (Table S1†). Substantial improvements in the measured mobilities could be achieved by the use of secondary dopants in ternary blend devices, an approach that has been previously reported for blends of PIDTBT with the symmetrical C_8_-BTBT semiconductor.^12,22^

To gain insights into the differences in device performance, atomic force microscopy (AFM) topography analysis (Fig. 1i–l) and grazing incidence X-ray diffraction (GIXD) experiments (Fig. 1m–p) were performed. AFM analysis showed aggregates forming on the top-layer of the blend film with varying morphologies depending on the BTBT molecule. The size and shape of these aggregates are consistent with the morphology of pristine BTBT films and are distinct from the amorphous topography of pristine PIDTBT (Fig. S11†). This indicates that the BTBT molecules vertically phase segregate from the polymer during thin-film formation. As highlighted by reports on C_8_-BTBT:PIDTBT, a vertically phase separated blend morphology consisting of highly ordered domains of the small-molecules on the upper surface of the film, interfacing with the dielectric layer (*i.e.* the conduction channel) in a top-gate device, is crucial for efficient charge transport.^12,22^

AFM images of 4eb/4ef:PIDTBT (Fig. 1i and j) show a connected terrace-like morphology which is in line with previous reports on unsymmetrical BTBT molecules.^13,15*d*^ GIXD of 4eb/4ef:PIDTBT films revealed crystalline peaks at 2*θ*/*θ* = 3.3° and 4.9° for 4eb, and 2*θ*/*θ* = 3.6° and 5.4° for 4ef which were similarly observed in the diffraction of their respective pristine films (Fig. 1k and l). This indicates that the BTBT molecules form ordered connected domains in the blend. On the other hand, the BTBT molecules in films of 4eg/4eh:PIDTBT (Fig. 1o and p) lead to large disconnected aggregates of the BTBT molecules. Here, the GIXD for 4eg has the same peak at 2*θ*/*θ* = 3.5° in the pristine and blend films indicating that the aggregates of 4eg are ordered but disconnected, while no clear peaks were observed in the diffraction of blends with 4eh suggesting the formation of large, disordered aggregates. Based on this observation, the higher *μ*_sat_ values in 4eb/4ef:PIDTBT devices are a result of better charge transport within the connected, ordered domains of the BTBT molecules on the top surface of the blend films. In addition, the HOMO levels of 4eb and 4ef closely match that of PIDTBT (Fig. 1e); this is crucial in minimizing energetic disorder for hole transport between the crystalline domains of small-molecules and the amorphous polymer.^12*e*,23^

## Conclusions

In summary, we have developed a modular synthetic approach to the unsymmetrical BTBT scaffold; an underexplored architecture in molecular semiconductors. The BTBT materials are prepared from readily available partners using a metal-free, Pummerer CH–CH-type cross-coupling followed by a Newman–Kwart reaction of the coupling products. Access to unsymmetric BTBT structures permitted the study of their material properties; varying the functional groups attached to the conjugated core of the BTBT scaffold modulated the molecular orbital energy levels and self-assembly properties. Preliminary investigation into the structure–property relationships of the unsymmetrical BTBT molecules in S-M:polymer blend transistors highlighted the influence of molecular structure on the charge transport ability in thin-films. We showed that devices fabricated using the phenyl and methoxy functionalized unsymmetrical BTBT molecules show higher hole mobilities than comparable devices fabricated using pristine PIDTBT or blends with the symmetrical C_8_-BTBT. Improved access to unsymmetrical BTBT molecular semiconductors using a modular synthetic approach provides a foundation for future studies targeting improved transistor performance, for example, through the use of secondary dopants such as fluorinated fullerene derivative, C_60_F_48_ or molecular Lewis acids, B(C_6_F_5_)_3_ and [Zn(C_6_F_5_)_2_] in ternary blends that have been widely studied in PIDTBT blends containing the symmetrical C_8_-BTBT.^12,22^

## Data availability

Crystallographic data for 4ef has been deposited at the CCDC under 2103832.

## Author contributions

Experiments were conceived and designed by M. T., A. R., G. J. P. P., M. L. T. and D. J. P. and executed by all co-authors. M. T. and G. J. P. P. synthesised and characterised the BTBT molecules. D. J. T. and R. M.-H. synthesised and characterised the polymer semiconductor PIDTBT. A. R., R. U. K., S. A., A. Z. performed the material characterization (DSC, CV, AFM) and the transistor fabrication and analysis. Y. S. and I. D. performed the POM experiment and analysis of the BTBT molecules. Y. J. performed the DFT calculations. I. V.-Y. acquired the XRD and single crystal measurements. M. T., A. R., G. J. P. P., M. L. T. and D. J. P. wrote the paper. M. L. T. and D. J. P. supervised the work. All authors contributed to the finalization of the paper.

## Conflicts of interest

There are no conflicts to declare.

## Supplementary Material

SC-013-D1SC05070B-s001

SC-013-D1SC05070B-s002

## References

[cit1] Paterson A. F., Singh S., Fallon K. J., Hodsden T., Han Y., Schroeder B. C., Bronstein H., Heeney M., McCulloch I., Anthopoulos T. D. (2018). Recent Progress in High-Mobility Organic Transistors: A Reality Check. Adv. Mater..

[cit2] Inganäs O. (2018). Organic Photovoltaics over Three Decades. Adv. Mater..

[cit3] Borges-González J., Kousseff C. J., Nielsen C. B. (2019). Organic Semiconductors for Biological Sensing. J. Mater. Chem. C.

[cit4] Bronstein H., Nielsen C. B., Schroeder B. C., McCulloch I. (2020). The Role of Chemical Design in the Performance of Organic Semiconductors. Nat. Rev. Chem..

[cit5] Rahmanudin A., Yao L., Sivula K. (2018). Conjugation Break Spacers and Flexible Linkers as Tools to Engineer the Properties of Semiconducting Polymers. Polym. J..

[cit6] Diao Y., Shaw L., Bao Z., Mannsfeld S. C. B. (2014). Morphology Control Strategies for Solution-Processed Organic Semiconductor Thin Films. Energy Environ. Sci..

[cit7] Takimiya K., Osaka I., Mori T., Nakano M. (2014). Organic Semiconductors Based on [1]Benzothieno[3,2-*b*][1]Benzothiophene Substructure. Acc. Chem. Res..

[cit8] Ma Z., Geng H., Wang D., Shuai Z. (2016). Influence of Alkyl Side-Chain Length on the Carrier Mobility in Organic Semiconductors: Herringbone vs. Pi–Pi Stacking. J. Mater. Chem. C.

[cit9] Ebata H., Izawa T., Miyazaki E., Takimiya K., Ikeda M., Kuwabara H., Yui T. (2007). Highly Soluble [1]Benzothieno[3,2-*b*]Benzothiophene (BTBT) Derivatives for High-Performance, Solution-Processed Organic Field-Effect Transistors. J. Am. Chem. Soc..

[cit10] Takimiya K., Ebata H., Sakamoto K., Izawa T., Otsubo T., Kunugi Y. (2006). 2,7-Diphenyl[1]Benzothieno[3,2-*b*]Benzothiophene, A New Organic Semiconductor for Air-Stable Organic Field-Effect Transistors with Mobilities up to 2.0 cm^2^ V^−1^ s^−1^. J. Am. Chem. Soc..

[cit11] Yuan Y., Giri G., Ayzner A. L., Zoombelt A. P., Mannsfeld S. C. B., Chen J., Nordlund D., Toney M. F., Huang J., Bao Z. (2014). Ultra-High Mobility Transparent Organic Thin Film Transistors Grown by an off-Centre Spin-Coating Method. Nat. Commun..

[cit12] Scaccabarozzi A. D., Scuratti F., Barker A. J., Basu A., Paterson A. F., Fei Z., Solomeshch O., Petrozza A., Tessler N., Heeney M., Anthopoulos T. D., Caironi M. (2020). Understanding Charge Transport in High-Mobility P-Doped Multicomponent Blend Organic Transistors. Adv. Electron. Mater..

[cit13] Iino H., Usui T., Hanna J. (2015). Liquid Crystals for Organic Thin-Film Transistors. Nat. Commun..

[cit14] Um M.-C., Kwak J., Hong J.-P., Kang J., Yoon D. Y., Lee S. H., Lee C., Hong J.-I. (2008). High-Performance Organic Semiconductors for Thin-Film Transistors Based on 2,7-Divinyl[1]Benzothieno[3,2-*b*]Benzothiophene. J. Mater. Chem..

[cit15] Kienle M., Unsinn A., Knochel P. (2010). Synthesis of Dibenzothiophenes and Related Classes of Heterocycles by Using Functionalized Dithiocarbamates. Angew. Chem., Int. Ed..

[cit16] Usluer Ö., Abbas M., Wantz G., Vignau L., Hirsch L., Grana E., Brochon C., Cloutet E., Hadziioannou G. (2014). Metal Residues in Semiconducting Polymers: Impact on the Performance of Organic Electronic Devices. ACS Macro Lett..

[cit17] Kitamura T., Morita K., Nakamori H., Oyamada J. (2019). Synthesis of [1]Benzothieno[3,2-*b*][1]Benzothiophene Derivatives via Successive Iodocyclization/Photocyclization of Alkynes. J. Org. Chem..

[cit18] Huang X., Maulide N. (2011). Sulfoxide-Mediated α-Arylation of Carbonyl Compounds. J. Am. Chem. Soc..

[cit19] Shrives H. J., Fernández-Salas J. A., Hedtke C., Pulis A. P., Procter D. J. (2017). Regioselective Synthesis of C3 Alkylated and Arylated Benzothiophenes. Nat. Commun..

[cit20] Kwart H., Evans E. R. (1966). The Vapor Phase Rearrangement of Thioncarbonates and Thioncarbamates. J. Org. Chem..

[cit21] Higashino T., Ueda A., Mori H. (2019). Di- and Tetramethoxy Benzothienobenzothiophenes: Substitution Position Effects on the Intermolecular Interactions, Crystal Packing and Transistor Properties. New J. Chem..

[cit22] Basu A., Niazi M. R., Scaccabarozzi A. D., Faber H., Fei Z., Anjum D. H., Paterson A. F., Boltalina O., Heeney M., Anthopoulos T. D. (2020). Impact of P-Type Doping on Charge Transport in Blade-Coated Small-Molecule:Polymer Blend Transistors. J. Mater. Chem. C.

[cit23] Smith J., Zhang W., Sougrat R., Zhao K., Li R., Cha D., Amassian A., Heeney M., McCulloch I., Anthopoulos T. D. (2012). Solution-Processed Small Molecule-Polymer Blend Organic Thin-Film Transistors with Hole Mobility Greater than 5 cm^2^/Vs. Adv. Mater..

