# Supplementary material for: Modular synthesis of unsymmetrical [1]benzothieno[3,2-b][1]benzothiophene molecular semiconductors for organic transistors
Source: Chem Sci. 2021 Nov 29;13(2):421–9. doi: 10.1039/d1sc05070b (PMC8730195; doi:10.1039/d1sc05070b)
Supplement: SC-013-D1SC05070B-s001 [file SC-013-D1SC05070B-s001.pdf]

Electronic Supplementary Material (ESI) for Chemical Science.
This journal is © The Royal Society of Chemistry 2021

Supporting Information

Part 1: Synthesis Section

Modular synthesis of unsymmetrical [1]benzothieno[3,2-b][1]benzothiophene (BTBT) molecular semiconductors for organic transistors

Masanori Tayu, Aiman Rahmanudin, Gregory J. P. Perry, Raja U. Khan, Daniel J. Tate, Raymundo Marcial-Hernandez, Yuan Shen, Ingo Dierking, Yurachat Janpatompong, Suphaluk Aphichatpanichakul, Adibah Zamhuri, Inigo Victoria-Yrezabal, Michael L. Turner* and David J. Procter*

Email: michael.turner@manchester.ac.uk, david.j.procter@manchester.ac.uk

Table of Contents

Part 1: Synthesis Section

1. **General synthesis procedures for BTBT derivatives** **S3**

   General Experimental **S3**

   General Procedure A: C-H Arylation of 3-Methoxybenzo[*b*]thiophene *S*-oxides **S4**

   General Procedure B: Synthesis of *S*-thiocarbamates **S4**

   General Procedure C: Hydrolysis and Cyclization to give BTBTs **S5**

2. **Experimental details for the synthesis of BTBT derivatives** **S6**

3. **^{1}H and ^{13}C NMR of BTBT derivatives** **S36**

4. **X-ray structure of 4ef** **S80**

Part 2: Materials Section

5. **Supporting figures and tables (as described in the main text)** **S83**

   Figure S1. Polarized optical microscopy of BTBT derivatives. **S83**

   Figure S2. Cyclic voltammograms of the BTBT derivatives. **S84**

   Figure S3. Transistor characteristics of 4eb semiconductor in bottom-gate/ top-contact configuration. **S85**

   Figure S4. Optical microscope images of the BTBT derivatives on SiO_2/ Si substrates. **S85**

   Figure S5-10. Representative transistor characteristics of pristine IDTBT and blends with BTBT derivatives. **S85-S90**

   Table S1. Summary of transistor parameters. **S91**

   Figure S11. Atomic force microscopy topography images of thin films. **S92**

6. **General experimental procedures** **S92**

   Synthesis **S92**

   Cyclic Voltammetry **S92**

   DFT Calculation **S93**

   DSC **S93**

   POM **S93**

   AFM **S94**

   OFET fabrication and characterization **S94-S95**

1. General synthesis procedures for BTBT derivatives

General Experimental

All solvents and reagents were purchased from commercial sources and used as supplied. ^{1}H NMR spectra were recorded on NMR spectrometers at 400 MHz and 500 MHz and ^{13}C NMR at 101 MHz and 126 MHz, using the residual solvent peak of $CDCl_3$ (^{1}H: $\delta = 7.26$; ^{13}C: $\delta = 77.0$). ^{1}H NMR chemical shifts (δ_H) and ^{13}C NMR chemical shifts (δ_C) are quoted in parts per million (ppm) and coupling constants (J) are quoted in Hertz (Hz). Abbreviations for NMR data are s (singlet), d (doublet), t (triplet), m (multiplet). Infrared (IR) spectra were recorded on a FTIR spectrometer and mass spectra were obtained using positive or negative electrospray ionization (ESI), atmospheric pressure chemical ionization (APCI) techniques. Column chromatography was carried out using silica gel 60 Å, 240-400 mesh. Thin layer chromatography (TLC) was performed on aluminum sheets pre-coated with silica gel, 0.20 mm (Merck TLC Silica gel 60 F_{254}). Melting points were measured on solids as obtained after recrystallization from EtOAc.

Benzothiophene *S*-oxides were prepared as previously reported.[1] The following compounds were prepared and characterized as previously reported: **3aa, 3eb** and **3eb`**.[1]

General Procedure A: C-H Arylation of 3-Methoxybenzo[*b*]thiophene *S*-oxides

Under a nitrogen atmosphere, 3-methoxybenzo[*b*]thiophene-*S*-oxides **1** (1.0 equiv) and phenols **2** (1.5 equiv) were dissolved in THF (0.1 M) in an oven-dried tube flushed with N_2. TFAA (1.5 equiv) was then added at −40 ºC. After 15 min at −40 ºC, the mixture was warmed to room temperature and stirred overnight. After removal of THF *in vacuo*, the residue was dissolved in CH_2Cl_2 (0.1 M). $BF_3{\cdot}OEt_2$ (0.20 equiv) was then added at room temperature, and the mixture was stirred at the same temperature for 30 min. The reaction was quenched with H_2O, and the aqueous layer was extracted with CH_2Cl_2 three times. The combined organic layers were dried over $MgSO_4$ and concentrated *in vacuo*. The crude product was purified by column chromatography on silica gel eluting with *n*-hexane and Et_2O to obtain the 2-aryl benzothiophene products of cross-coupling **3**.

General Procedure B: Synthesis of *S*-thiocarbamates

Under a nitrogen atmosphere, NaH (3.0 equiv) was added to a solution of the 2-aryl benzothiophene products of cross-coupling **3** in DMF (0.1 M) at room temperature. After 15 min at the same temperature, dimethylthiocarbamoyl chloride (2.0 equiv) was added to the reaction mixture. After the consumption of **3** (average reaction time = 16 h), the reaction was quenched with saturated aqueous NH_4Cl and the aqueous layer was extracted with Et_2O three times. The combined organic layers were dried over $MgSO_4$ and concentrated *in vacuo*. The *O*-thiocarbamate product was used in the next step without further purification.

The *O*-thiocarbamate was dissolved in Ph_2O (0.1 M) and the solution heated at reflux. After completion of the reaction (average reaction time = 1 h), the crude product was purified by column chromatography on silica gel eluting with *n*-hexane in Et_2O to obtain the *S*-thiocarbamate **5**.

General Procedure C: Hydrolysis and Cyclization to give BTBTs

KOMe (2.0 equiv) was added to a solution of *S*-thiocarbamate **5** in methanol/toluene (1:1 ratio, 0.05 M) in a 10 mL microwave vial. The vial was capped and heated to 120 ºC. After the consumption of **5** (average reaction time = 1 h), the reaction mixture was cooled to room temperature. TsOH·H_2O (8.0 equiv) was added to the crude mixture and then the solution was heated at 120 ºC. After the completion of the reaction (average reaction time = 1 h), the reaction mixture was cooled to room temperature. Water was added to the reaction mixture, and then the aqueous layer was extracted with Et_2O three times. The combined organic layers were dried over $MgSO_4$ and concentrated *in vacuo*. The crude product was purified by column chromatography on silica gel eluting with *n*-hexane in Et_2O or recrystallized to afford BTBT **4**.

2. Experimental details for the synthesis of BTBT derivatives

Compounds **3aa**, **3eb** and **3eb'** have previously been synthesized and reported by our group.[1]

4-(3-Methoxybenzo[*b*]thiophen-2-yl)-[1,1'-biphenyl]-3-ol (3ab)

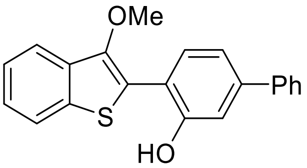

1a (72 mg, 0.40 mmol), TFAA (83 μL, 0.60 mmol), THF (4.0 mL), 3-phenylphenol (**2b**; 113 mg, 0.60 mmol), $BF_3 \cdot OEt_2$ (10 μL, 0.08 mmol), CH_2Cl_2 (4.0 mL), gave **3ab** as a white solid (61 mg, 0.18 mmol, 46%); **M.p.**: 127-129 ºC; **IR** (ATR): 3272, 1348, 1060, 759 cm^{-1}; **^{1}H-NMR** (400 MHz, $CDCl_3$) δ: 8.15 (1H, s, O*H*), 7.83-7.76 (2H, m, Ar*H*), 7.71-7.64 (2H, m, Ar*H*), 7.57 (1H, d, *J* = 8.1 Hz, Ar*H*), 7.51-7.44 (3H, m, Ar*H*), 7.44-7.35 (3H, m, Ar*H*), 7.27 (1H, dd, *J* = 8.1, 1.7 Hz, Ar*H*), 3.94 (3H, s, OC*H*3); **^{13}C-NMR** (126 MHz, $CDCl_3$) δ: 154.4 (Ar*C*(OH)), 144.6 (Ar*C*), 143.4 (Ar*C*), 140.1 (Ar*C*), 137.1 (Ar*C*), 133.4 (Ar*C*), 131.0 (Ar*C*H), 128.8 (Ar*C*H), 127.7 (Ar*C*H), 127.0 (Ar*C*H), 125.8 (Ar*C*), 125.3 (Ar*C*H), 124.6 (Ar*C*H), 122.8 (Ar*C*H), 120.6 (Ar*C*H), 119.6 (Ar*C*H), 118.1 (Ar*C*), 116.9 (Ar*C*H), 62.4 (O*C*H3); **HRMS** (APCI): Calcd. for $C_{21}H_{17}O_2S$ $[M+H]^+$: 333.0944; found: 333.0940.

5-Bromo-2-(3-methoxybenzo[*b*]thiophen-2-yl)phenol (3ac)

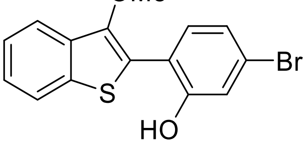

1a (72 mg, 0.40 mmol), TFAA (83 μL, 0.60 mmol), THF (4.0 mL), 3-bromophenol (**2c**; 104 mg, 0.60 mmol), $BF_3 \cdot OEt_2$ (10 μL, 0.08 mmol), CH_2Cl_2 (4.0 mL), gave **3ac** as a colorless oil (109 mg,

[1] Z. He, H. J. Shrives, J. A. Fernández-Salas, A. Abengózar, J. Neufeld, K. Yang, A. P. Pulis and D. J. Procter, *Angew. Chem. Int. Ed.* 2018, **57**, 5759–5764.

0.33 mmol; 81%); **IR** (ATR): 3245, 1478, 1349, 800 cm^{-1}; **^{1}H-NMR** (500 MHz, $CDCl_3$) δ: 8.13 (1H, s, O*H*), 7.80-7.69 (2H, m, Ar*H*), 7.45-7.34 (2H, m, Ar*H*), 7.32 (1H, d, *J* = 8.4 Hz, Ar*H*), 7.26 (1H, s, Ar*H*), 7.11 (1H, d, *J* = 8.4 Hz, Ar*H*), 3.87 (3H, s, OC*H*$_3$); **^{13}C-NMR** (126 MHz, $CDCl_3$) δ: 154.8 (Ar*C*(OH)), 144.8 (Ar*C*), 137.0 (Ar*C*), 133.2 (Ar*C*), 131.5 (Ar*C*H), 125.5 (Ar*C*H), 124.8 (Ar*C*), 124.7 (Ar*C*H), 124.0 (Ar*C*H), 123.8 (Ar*C*), 122.8 (Ar*C*H), 121.7 (Ar*C*H), 120.7 (Ar*C*H), 118.3 (Ar*C*), 62.4 (O*C*H$_3$); **HRMS** (ESI): Calcd. for $C_{15}H_{10}O_2BrS$ [M-H]$^-$: 332.9590; found: 332.9586.

5-(*tert*-Butyl)-2-(3-methoxybenzo[*b*]thiophen-2-yl)phenol (3ad)

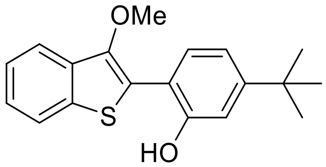

1a (72 mg, 0.40 mmol), TFAA (83 μL, 0.60 mmol), THF (4.0 mL), 3-*tert*-butylphenol (**2d**; 90 mg, 0.60 mmol), $BF_3 \cdot OEt_2$ (10 μL, 0.08 mmol), CH_2Cl_2 (4.0 mL), gave **3ad** as a colorless oil (84 mg, 0.27 mmol; 67%); **IR** (ATR): 3293, 1347, 1069, 809 cm^{-1}; **^{1}H-NMR** (500 MHz, $CDCl_3$) δ: 8.00 (1H, s, O*H*), 7.77 (1H, d, *J* = 8.0 Hz, Ar*H*), 7.75 (1H, d, *J* = 8.0 Hz, Ar*H*), 7.46-7.34 (3H, m, Ar*H*), 7.12 (1H, d, *J* = 2.0 Hz, Ar*H*), 7.04 (1H, dd, *J* = 8.0, 2.0 Hz, Ar*H*), 3.90 (3H, s, OC*H*$_3$), 1.36 (9H, s, (C*H*$_3$)$_3$); **^{13}C-NMR** (126 MHz, $CDCl_3$) δ: 154.2 (Ar*C*(OH)), 153.7 (Ar*C*), 144.2 (Ar*C*), 136.9 (Ar*C*), 133.5 (Ar*C*), 130.1 (Ar*C*H), 126.2 (Ar*C*), 125.0 (Ar*C*H), 124.5 (Ar*C*H), 122.7 (Ar*C*H), 120.5 (Ar*C*H), 118.1 (Ar*C*H), 116.1 (Ar*C*), 115.5 (Ar*C*H), 62.3 (O*C*H$_3$), 34.7 (*C*(CH$_3$)$_3$), 31.1(C(*C*H$_3$)$_3$); **HRMS** (APCI): Calcd. for $C_{19}H_{20}BrO_2S$ [M]$^+$: 391.0362; found: 391.0354.

3-(3-Methoxybenzo[*b*]thiophen-2-yl)-[1,1'-biphenyl]-4-ol (3ae)

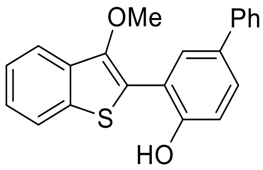

1a (72 mg, 0.40 mmol), TFAA (83 μL, 0.60 mmol), THF (4.0 mL), 4-phenylphenol (**3e**; 102 mg, 0.60 mmol), $BF_3 \cdot OEt_2$ (10 μL, 0.08 mmol), CH_2Cl_2 (4.0 mL), gave **3ae** as a colorless amorphous solid (98 mg, 0.29 mmol, 74%); **IR** (ATR): 3327, 1347, 1260, 1092, 762 cm^{-1}; ^{1}**H-NMR** (400 MHz, $CDCl_3$) δ: 8.04 (1H, s, O*H*), 7.84-7.77 (2H, m, Ar*H*), 7.75 (1H, s, Ar*H*), 7.67-7.61 (2H, m, Ar*H*), 7.59 (1H, dd, *J* = 8.6, 2.0 Hz, Ar*H*), 7.51-7.39 (4H, m, Ar*H*), 7.36 (1H, t, *J* = 7.3 Hz, Ar*H*), 7.19 (1H, d, *J* = 8.6 Hz, Ar*H*), 3.94 (3H, s, OCH_3); ^{13}**C-NMR** (126 MHz, $CDCl_3$) δ: 153.7 (Ar*C*(OH)), 144.9 (Ar*C*), 140.3 (Ar*C*), 137.1 (Ar*C*), 134.0 (Ar*C*), 133.4 (Ar*C*), 129.2 (Ar*C*H), 129.1 (Ar*C*H), 128.8 (Ar*C*H), 126.9 (Ar*C*H), 126.8 (Ar*C*H), 125.6 (Ar*C*), 125.3 (Ar*C*H), 124.6 (Ar*C*H), 122.8 (Ar*C*H), 120.7 (Ar*C*H), 119.4 (Ar*C*), 118.9 (Ar*C*H), 62.4 (O*C*H_3); **HRMS** (APCI): Calcd. for $C_{21}H_{17}O_2S$ $[M+H]^+$: 333.0930; found: 333.0932.

4-(6-Bromo-3-methoxybenzo[*b*]thiophen-2-yl)-[1,1'-biphenyl]-3-ol (3bb)

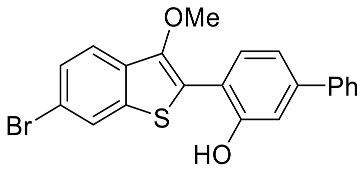

1b (78 mg, 0.30 mmol), TFAA (63 μL, 0.45 mmol), THF (3.0 mL), 3-phenylphenol (**2b**; 68 mg, 0.45 mmol), $BF_3 \cdot OEt_2$ (7 μL, 0.06 mmol), CH_2Cl_2 (3.0 mL), gave **3bb** as a white solid (76 mg, 0.18 mmol, 61%); **M.p.**: 135-136 ºC; **IR** (ATR): 3280, 1258, 1015, 790 cm^{-1}; ^{1}**H-NMR** (400 MHz, $CDCl_3$) δ: 7.96 (1H, s, O*H*), 7.93 (1H, d, *J* = 1.5 Hz, Ar*H*), 7.68-7.64 (2H, m, Ar*H*), 7.62 (1H, d, *J* = 8.6 Hz, Ar*H*), 7.56-7.51 (2H, m, Ar*H*), 7.51-7.43 (2H, m, Ar*H*), 7.38 (1H, t, *J* = 8.6 Hz, Ar*H*), 7.35 (1H, d, *J* = 1.5 Hz, Ar*H*), 7.26 (1H, dd, *J* = 8.6, 1.5 Hz, Ar*H*), 3.91 (3H, s, OCH_3); ^{13}**C-NMR** (126 MHz, $CDCl_3$) δ: 154.3 (Ar*C*), 144.4 (Ar*C*), 143.6 (Ar*C*), 139.9 (Ar*C*), 138.4 (Ar*C*), 132.3

(Ar*C*), 130.9 (Ar*C*H), 128.8 (Ar*C*H), 128.1 (Ar*C*H), 127.8 (Ar*C*H), 127.0 (Ar*C*H), 126.2 (Ar*C*), 125.3 (Ar*C*H), 121.7 (Ar*C*H), 119.7 (Ar*C*H), 119.2 (Ar*C*), 117.6 (Ar*C*), 116.9 (Ar*C*H), 62.5 (O*C*H$_3$); **HRMS** (ESI): Calcd. for $C_{21}H_{14}BrO_2S$ [M-H]$^-$: 408.9903; found: 408.9903.

2-(6-Bromo-3-methoxybenzo[b]thiophen-2-yl)-5-(tert-butyl)phenol (3bd)

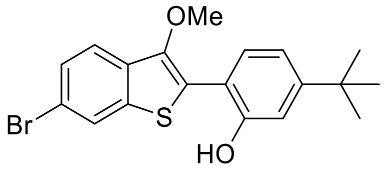

1b (0.11 g, 0.42 mmol), TFAA (88 μL, 0.63 mmol), THF (4.2 mL), 3-*tert*-butylphenol (**2d**; 95 mg, 0.63 mmol), BF_3·OEt_2 (10 μL, 0.08 mmol), CH_2Cl_2 (4.2 mL), gave **3bd** as a white solid (133 mg, 0.34 mmol, 81%); **M.p.**: 120-121 ºC; **IR** (ATR): 3288, 1067, 1020, 791 cm^{-1}; ^{1}**H-NMR** (500 MHz, $CDCl_3$) δ: 7.91 (1H, s, O*H*), 7.83 (1H, s, Ar*H*), 7.59 (1H, d, *J* = 8.4 Hz, Ar*H*), 7.51 (1H, d, *J* = 8.4 Hz, Ar*H*), 7.39 (1H, d, *J* = 8.4 Hz, Ar*H*), 7.12 (1H, s, Ar*H*), 7.03 (1H, d, *J* = 8.4 Hz, Ar*H*), 3.87 (3H, s, OC*H$_3$*), 1.35 (9H, s, C(C*H$_3$*)$_3$); ^{13}**C-NMR** (126 MHz, $CDCl_3$) δ: 154.6 (Ar*C*), 153.7 (Ar*C*), 143.9 (Ar*C*), 138.2 (Ar*C*), 132.3 (Ar*C*), 130.0 (Ar*C*H), 128.0 (Ar*C*H), 126.6 (Ar*C*), 125.2 (Ar*C*H), 121.6 (Ar*C*H), 118.9 (Ar*C*), 118.2 (Ar*C*H), 115.6 (Ar*C*), 115.5 (Ar*C*H), 62.4 (O*C*H$_3$), 34.7 (*C*(CH$_3$)$_3$), 31.1 (C(*C*H$_3$)$_3$); **HRMS** (ESI): Calcd. for $C_{19}H_{18}BrO_2S$ [M-H]$^-$: 389.0216; found: 389.0216.

2-(6-Bromo-3-methoxybenzo[b]thiophen-2-yl)-5-methoxyphenol (3bf)

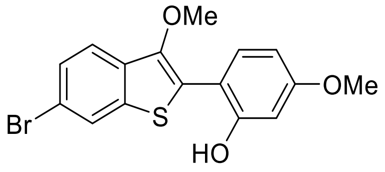

1b (0.10 g, 0.39 mmol), TFAA (82 μL, 0.59 mmol), THF (3.9 mL), 3-methoxyphenol (**2f**; 64 μL, 0.59 mmol), BF_3·OEt_2 (10 μL, 0.08 mmol), CH_2Cl_2 (3.9 mL), gave **3bf** as a white solid (115 mg,

0.31, 82%); **M.p.**: 100-101 °C; IR (ATR): 3276, 1617, 1070, 795 cm^{-1}; ^{1}**H-NMR** (400 MHz, $CDCl_3$) δ: 7.96 (1H, s, O*H*), 7.90 (1H, d, *J* = 1.5 Hz, Ar*H*), 7.58 (1H, d, *J* = 8.3 Hz, Ar*H*), 7.51 (1H, dd, *J* = 8.6, 1.5 Hz, Ar*H*), 7.36 (1H, d, *J* = 8.8 Hz, Ar*H*), 6.63 (1H, d, *J* = 2.4 Hz, Ar*H*), 6.59 (1H, dd, *J* = 8.6, 2.7 Hz, Ar*H*), 3.85 (3H, s, OCH_3), 3.84 (3H, s, OCH_3); ^{13}**C-NMR** (101 MHz, $CDCl_3$) δ: 161.9 (Ar*C*), 155.3 (Ar*C*), 143.3 (Ar*C*), 137.9 (Ar*C*), 132.3 (Ar*C*), 131.2 (Ar*C*H), 127.9 (Ar*C*H), 126.4 (Ar*C*), 125.1 (Ar*C*H), 121.4 (Ar*C*H), 118.7 (Ar*C*), 111.2 (Ar*C*), 108.0 (Ar*C*H), 102.9 (Ar*C*H), 62.1 (O*C*H_3), 55.4 (O*C*H_3); **HRMS** (APCI): Calcd. for $C_{16}H_{14}BrO_3S$ $[M+H]^+$: 364.9842; found: 364.9837.

2-(6-Bromo-3-methoxybenzo[*b*]thiophen-2-yl)-5-(trifluoromethyl)phenol (3bg)

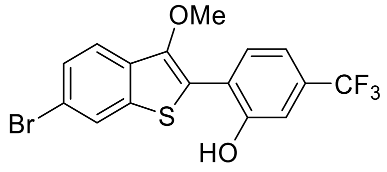

1b (0.10 g, 0.39 mmol), TFAA (82 μL, 0.58 mmol), THF (3.9 mL), 3-(trifluoromethyl)phenol (**2g**; 70 μL, 0.58 mmol), $BF_3·OEt_2$ (10 μL, 0.08 mmol), CH_2Cl_2 (3.9 mL), gave **3bg** as a white solid (128 mg, 0.32, 82%); **M.p.**: 98-100 °C; **IR** (ATR): 3245, 1334, 1115, 1065, 811 cm^{-1}; ^{1}**H-NMR** (400 MHz, $CDCl_3$) δ: 8.09 (1H, s, O*H*), 7.93 (1H, d, *J* = 1.5 Hz, Ar*H*), 7.63 (1H, d, *J* = 8.5 Hz, Ar*H*), 7.60-7.49 (2H, m, Ar*H*), 7.36 (1H, s, Ar*H*), 7.25 (1H, dd, *J* = 8.5, 1.5 Hz, Ar*H*), 3.89 (3H, s, OCH_3); ^{13}**C-NMR** (101 MHz, $CDCl_3$) δ: 154.2 (Ar*C*), 145.3 (Ar*C*), 138.6 (Ar*C*), 132.4 (q, *J* = 32.9 Hz, Ar*C*CF_3), 131.9 (Ar*C*), 131.0 (Ar*C*H), 128.4 (Ar*C*H), 125.4 (Ar*C*H), 124.7 (Ar*C*), 123.7 (q, *J* = 270.8 Hz, C*C*F_3), 122.1 (Ar*C*), 122.0 (Ar*C*H), 119.8 (Ar*C*), 117.4 (q, *J* = 3.7 Hz, Ar*C*H), 115.8 (q, *J* = 3.7 Hz, Ar*C*H), 62.7 (O*C*H_3); **HRMS** (APCI): Calcd. for $C_{16}H_{11}BrF_3O_2S$ $[M+H]^+$: 402.9610; found: 402.9613.

5-(*tert*-Butyl)-2-(3-methoxy-6-phenylbenzo[*b*]thiophen-2-yl)phenol (3cd)

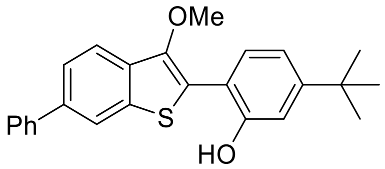

1c (77 mg, 0.30 mmol), TFAA (63 μL, 0.45 mmol), THF (3.0 mL), 3-*tert*-butylphenol (68 mg, 0.45 mmol), $BF_3 \cdot OEt_2$ (7 μL, 0.06 mmol), CH_2Cl_2 (3.0 mL), gave **3cd** as a white solid (105 mg, 0.27 mmol, 90%); **M.p.**: 119-120 ºC; **IR** (ATR): 3279, 1352, 1060, 764 cm^{-1}; **^{1}H-NMR** (400 MHz, $CDCl_3$) δ: 8.07 (1H, s, O*H*), 7.98 (1H, d, *J* = 1.0 Hz, Ar*H*), 7.81 (1H, d, *J* = 8.0 Hz, Ar*H*), 7.73-7.62 (3H, m, Ar*H*), 7.54-7.46 (2H, m, Ar*H*), 7.45 (1H, d, *J* = 8.0 Hz, Ar*H*), 7.39 (1H, t, *J* = 8.0 Hz, Ar*H*), 7.15 (1H, d, *J* = 2.0 Hz, Ar*H*), 7.06 (1H, dd, *J* = 8.0, 2.0 Hz, Ar*H*), 3.93 (3H, s, OCH_3), 1.37 (9H, s, C(CH_3)$_3$); **^{13}C-NMR** (101 MHz, $CDCl_3$) δ: 154.3 (Ar*C*), 153.7 (Ar*C*), 144.0 (Ar*C*), 140.8 (Ar*C*), 138.4 (Ar*C*), 137.6 (Ar*C*), 132.6 (Ar*C*), 130.1 (Ar*C*H), 128.9 (Ar*C*H), 127.4 (Ar*C*H), 127.3 (Ar*C*H), 126.6 (Ar*C*), 124.2 (Ar*C*H), 121.1 (Ar*C*H), 120.7 (Ar*C*H), 118.1 (Ar*C*H), 116.1 (Ar*C*), 115.5 (Ar*C*H), 62.4 (O*C*H$_3$), 34.7(*C*(CH$_3$)$_3$), 31.1(C(*C*H$_3$)$_3$); **HRMS** (APCI): Calcd. for $C_{25}H_{23}O_2S$ $[M-H]^-$: 387.1424; found: 387.1429.

3-(3-Methoxy-6-phenylbenzo[*b*]thiophen-2-yl)-[1,1'-biphenyl]-4-ol (3ce)

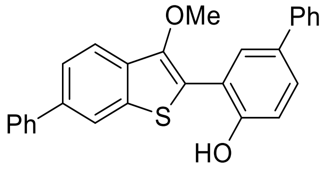

1c (71 mg, 0.28 mmol), TFAA (58 μL, 0.42 mmol), THF (2.8 mL), 4-phenylphenol (**3e**; 73 mg, 0.42 mmol), $BF_3 \cdot OEt_2$ (7 μL, 0.06 mmol), CH_2Cl_2 (2.8 mL), gave **3ce** as a white powder (87 mg, 0.21 mmol, 77%); **M.p.**: 196-198 ºC; **IR** (ATR): 3232, 1257, 1011, 787 cm^{-1}; **^{1}H-NMR** (400 MHz, $CDCl_3$) δ: 8.04 (1H, s, O*H*), 8.00 (1H, d, *J* = 1.0 Hz, Ar*H*), 7.84 (1H, d, *J* = 8.3 Hz, Ar*H*), 7.73 (1H, d, *J* = 2.2 Hz, Ar*H*), 7.71-7.65 (3H, m, Ar*H*), 7.64-7.60 (2H, m, Ar*H*), 7.58 (1H, dd, *J* = 8.3,

2.2 Hz, Ar*H*), 7.53-7.43 (4H, m, Ar*H*), 7.39 (1H, t, *J* = 7.3 Hz, Ar*H*), 7.35 (1H, t, *J* = 7.3 Hz, Ar*H*), 7.18 (1H, d, *J* = 8.3 Hz, Ar*H*), 3.96 (3H, s, OC*H*$_3$); **^{13}C-NMR** (101 MHz, CDCl$_3$) δ: 153.6 (Ar*C*), 144.7 (Ar*C*), 140.7 (Ar*C*), 140.3 (Ar*C*), 138.7 (Ar*C*), 137.8 (Ar*C*), 134.0 (Ar*C*), 132.5 (Ar*C*), 129.19 (Ar*C*H), 129.16 (Ar*C*H), 128.9 (Ar*C*H), 128.8 (Ar*C*H), 127.5 (Ar*C*H), 127.4 (Ar*C*H), 127.0 (Ar*C*H), 126.8 (Ar*C*H), 126.0 (Ar*C*), 124.4 (Ar*C*H), 121.1 (Ar*C*H), 120.9 (Ar*C*H), 119.4 (Ar*C*), 118.9 (Ar*C*H), 62.5 (O*C*H$_3$); **HRMS** (ESI): Calcd. for $C_{27}H_{21}O_2S$ [M+H]$^+$: 409.1257; found: 409.1255.

2-(6-Decyl-3-methoxybenzo[*b*]thiophen-2-yl)-5-methoxyphenol (3ef)

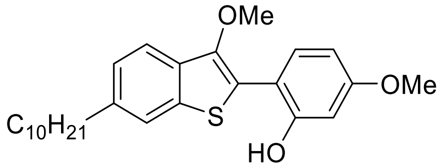

1e (94 mg, 0.29 mmol), TFAA (61 *μ*L, 0.44 mmol), THF (2.9 mL), 3-methoxyphenol (**2f**; 48 *μ*L, 0.44 mmol), BF$_3$·OEt$_2$ (7 *μ*L, 0.06 mmol), CH$_2$Cl$_2$ (2.9 mL), gave **3ef** as a colorless oil (91 mg, 0.21 mmol, 73%); **IR** (ATR): 3231, 1346, 1326, 1090 cm^{-1}; **^{1}H-NMR** (500 MHz, CDCl$_3$) δ: 8.11 (1H, s, O*H*), 7.64 (1H, d, *J* = 8.1 Hz, Ar*H*), 7.56 (1H, s, Ar*H*), 7.39 (1H, d, *J* = 8.6 Hz, Ar*H*), 7.24 (1H, d, *J* = 8.1 Hz, Ar*H*), 6.64 (1H, d, *J* = 2.4 Hz, Ar*H*), 6.59 (1H, dd, *J* = 8.6, 2.4 Hz, Ar*H*), 3.87 (3H, s, OC*H*$_3$), 3.84 (3H, s, OC*H*$_3$), 2.73 (2H, t, *J* = 7.0 Hz, ArC*H*$_2$), 1.68 (2H, m, C*H*$_2$), 1.45-1.16 (14H, m, C*H*$_2$), 0.90 (3H, t, *J* = 7.0 Hz, C*H*$_3$); **^{13}C-NMR** (126 MHz, CDCl$_3$) δ: 161.6 (Ar*C*(OH)), 155.3 (Ar*C*), 143.6 (Ar*C*), 140.3 (Ar*C*), 137.0 (Ar*C*), 131.5 (Ar*C*), 131.2 (Ar*C*H), 125.6 (Ar*C*H), 124.6 (Ar*C*), 122.0 (Ar*C*H), 120.1 (Ar*C*H), 111.9 (Ar*C*), 107.8 (Ar*C*H), 102.9 (Ar*C*H), 62.0 (O*C*H$_3$), 55.4 (O*C*H$_3$), 36.1 (*C*H$_2$), 31.9 (*C*H$_2$), 31.7 (*C*H$_2$), 29.6 (*C*H$_2$), 29.6 (*C*H$_2$), 29.5 (*C*H$_2$), 29.3 (*C*H$_2$), 29.3 (*C*H$_2$), 22.7 (*C*H$_2$), 14.1 (*C*H$_3$); **HRMS** (APCI): Calcd. for $C_{26}H_{35}O_3S$ [M+H]$^+$: 427.2301; found: 427.2300.

2-(6-Decyl-3-methoxybenzo[*b*]thiophen-2-yl)-5-(trifluoromethyl)phenol (3eg)

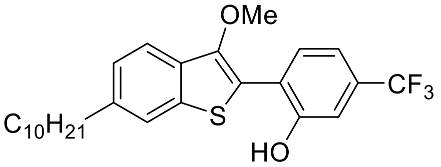

1e (69 mg, 0.22 mmol), TFAA (45 μL, 0.32 mmol), THF (2.0 mL), 3-(trifluoromethyl)phenol (**2g**; 39 μL, 0.32 mmol), $BF_3 \cdot OEt_2$ (5 μL, 0.04 mmol), CH_2Cl_2 (2.0 mL), gave **3eg** as a colorless oil (75 mg, 0.16 mmol, 75%); **IR** (ATR): 3252, 1332, 1125, 800 cm^{-1}; **^{1}H-NMR** (500 MHz, $CDCl_3$) δ: 8.27 (1H, s, O*H*), 7.68 (1H, d, *J* = 8.0 Hz, Ar*H*), 7.59 (1H, s, Ar*H*), 7.57 (1H, d, *J* = 8.0 Hz, Ar*H*), 7.34 (1H, s, Ar*H*), 7.27 (1H, d, *J* = 8.0 Hz, Ar*H*), 7.23 (1H, d, *J* = 8.0 Hz, Ar*H*), 3.91 (3H, s, OCH_3), 2.74 (2H, t, *J* = 7.5 Hz, ArCH_2), 1.68 (2H, m, CH_2), 1.45-1.14 (14H, m, CH_2), 0.88 (3H, t, *J* = 7.5 Hz, CH_3); **^{13}C-NMR** (126 MHz, $CDCl_3$) δ: 154.2 (Ar*C*(OH)), 145.5 (Ar*C*), 141.3 (Ar*C*), 137.7 (Ar*C*), 131.9 (q, *J* = 32.1 Hz, Ar*C*CF_3), 131.1 (Ar*C*), 131.0 (Ar*C*H), 126.0 (Ar*C*H), 123.8 (q, *J* = 271.3 Hz, *C*F_3), 123.2 (Ar*C*), 122.8, (Ar*C*) 122.1 (Ar*C*H), 120.6 (Ar*C*H), 117.2 (q, *J* = 3.6 Hz, Ar*C*H), 115.7 (q, *J* = 3.6 Hz, Ar*C*H), 62.6 (O*C*H_3), 36.1 (*C*H_2), 31.9 (*C*H_2), 31.6 (*C*H_2), 29.6 (*C*H_2), 29.6 (*C*H_2), 29.5 (*C*H_2), 29.3 (*C*H_2), 29.3 (*C*H_2), 22.7 (*C*H_2), 14.1 (*C*H_3); **HRMS** (APCI): Calcd. for $C_{26}H_{32}F_3O_2S$ $[M+H]^+$: 465.2070; found: 465.2073.

2-(6-Decyl-3-methoxybenzo[*b*]thiophen-2-yl)-5-(dimethylamino)phenol (3eh)

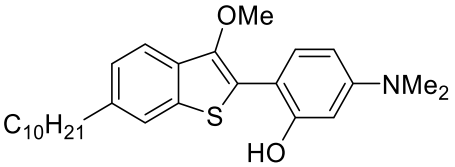

1e (0.10 g, 0.33 mmol), TFAA (68 μL, 0.49 mmol), THF (3.3 mL), 3-(dimethylamino)phenol (**2h**; 67 mg, 0.49 mmol), $BF_3 \cdot OEt_2$ (8 μL, 0.07 mmol), CH_2Cl_2 (3.3 mL), gave **3eh** as a white solid (72 mg, 0.16 mmol, 50%); **M.p.**:78-79 ºC; **IR** (ATR): 3284, 1258, 1015 cm^{-1}; **^{1}H-NMR** (400 MHz, $CDCl_3$) δ: 8.13 (1H, s, O*H*), 7.60 (1H, d, *J* = 8.5 Hz, Ar*H*), 7.54 (1H, s, Ar*H*), 7.33 (1H, d, *J* = 8.5

Hz, Ar*H*), 7.21 (1H, d, *J* = 8.5 Hz, Ar*H*), 6.42-6.38 (1H, m, Ar*H*), 6.41 (1H, s, Ar*H*), 3.86 (3H, s, OCH_3), 3.00 (6H, s, N(CH_3)$_2$), 2.72 (2H, t, *J* = 7.5 Hz, ArCH_2), 1.74-1.60 (2H, m, CH_2), 1.41-1.16 (14H, m, CH_2), 0.89 (3H, t, *J* = 7.5 Hz, CH_3); **^{13}C-NMR** (126 MHz, $CDCl_3$) δ: 155.0 (Ar*C*(OH)), 152.3 (Ar*C*), 142.7 (Ar*C*), 139.8 (Ar*C*), 136.6 (Ar*C*), 131.7 (Ar*C*), 131.1 (Ar*C*H), 125.9 (Ar*C*), 125.5 (Ar*C*H), 121.9 (Ar*C*H), 119.8 (Ar*C*H), 107.5 (Ar*C*), 105.6 (Ar*C*H), 100.9 (Ar*C*H), 61.8 (O*C*H_3), 40.3 (N(*C*H_3)$_2$), 36.1 (*C*H_2), 31.9 (*C*H_2), 31.7 (*C*H_2), 29.6 (*C*H_2), 29.6 (*C*H_2), 29.5 (*C*H_2), 29.3 (*C*H_2), 29.3 (*C*H_2), 22.7 (*C*H_2), 14.1 (*C*H_3); **HRMS** (APCI): Calcd. for $C_{27}H_{38}NO_2S$ $[M+H]^+$: 440.2618; found: 440.2607.

_S_-(2-(3-Methoxybenzo[_b_]thiophen-2-yl)phenyl) dimethylcarbamothioate (5aa)

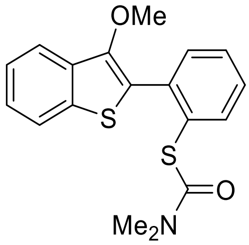

3aa (57 mg, 0.20 mmol), NaH (24 mg, 0.60 mmol), dimethylthiocarbamoyl chloride (43 mg, 0.40 mmol), DMF (2.0 mL), Ph_2O (4.0 mL), gave **5aa** as a colorless oil (61 mg, 0.18 mmol, 89%); **IR** (ATR): 1262, 1088, 1017, 800, 734 cm^{-1}; **^{1}H-NMR** (500 MHz, $CDCl_3$) δ: 7.80 (1H, d, *J* = 7.8 Hz, Ar*H*), 7.74 (1H, d, *J* = 7.8 Hz, Ar*H*), 7.68 (1H, dd, *J* = 5.8, 3.5 Hz, Ar*H*), 7.60 (1H, dd, *J* = 5.8, 3.5 Hz, Ar*H*), 7.48-7.42 (2H, m, Ar*H*), 7.42-7.33 (2H, m, Ar*H*), 3.67 (3H, s, OCH_3), 2.96 (6H, s, N(CH_3)$_2$); **^{13}C-NMR** (126 MHz, $CDCl_3$) δ: (1 × Ar*C*H missing), 166.3 (*C*O), 147.6 (Ar*C*), 137.7 (Ar*C*H), 137.5 (Ar*C*), 137.0 (Ar*C*), 133.6 (Ar*C*), 132.3 (Ar*C*H), 130.3 (Ar*C*), 129.0 (Ar*C*H), 124.9 (Ar*C*H), 123.9 (Ar*C*H), 122.3 (Ar*C*H), 121.3 (Ar*C*), 121.2 (Ar*C*H), 60.9 (O*C*H_3), 37.0 (N(*C*H_3)$_2$); **HRMS** (APCI): Calcd. for $C_{18}H_{18}NO_2S_2$ $[M+H]^+$: 344.0773; found: 344.0772.

_S_-(4-(3-Methoxybenzo[b]thiophen-2-yl)-[1,1'-biphenyl]-3-yl) dimethylcarbamothioate (5ab)

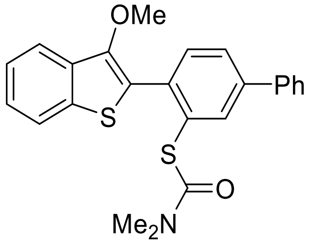

3ab (53 mg, 0.16 mmol), NaH (60%, 19 mg, 0.48 mmol), dimethylthiocarbamoyl chloride (34 mg, 0.32 mmol), DMF (1.6 mL), Ph_2O (1.6 mL), gave **5ab** as a white solid (49 mg, 0.12 mmol, 73%); **M.p.**: 106-108 °C; **IR** (ATR): 1664, 1349, 1095, 1061 cm^{-1}; **^{1}H-NMR** (500 MHz, $CDCl_3$) δ: 7.92 (1H, s, Ar*H*), 7.81 (1H, d, *J* = 7.6 Hz, Ar*H*), 7.75 (1H, d, *J* = 7.6 Hz, Ar*H*), 7.70-7.63 (4H,

m, Ar*H*), 7.49-7.43 (2H, m, Ar*H*), 7.43-7.33 (3H, m, Ar*H*), 3.73 (3H, s, OCH_3), 2.98 (6H, s, N(CH_3)$_2$); **^{13}C-NMR** (126 MHz, $CDCl_3$) δ: 166.1 (*C*O), 147.8 (Ar*C*), 141.9 (Ar*C*), 139.7 (Ar*C*), 137.0 (Ar*C*), 136.3 (Ar*C*H), 136.2 (Ar*C*), 133.6 (Ar*C*), 132.6 (Ar*C*H), 130.6 (Ar*C*), 128.8 (Ar*C*H), 127.73 (Ar*C*H), 127.65 (Ar*C*H), 127.3 (Ar*C*H), 124.9 (Ar*C*H), 123.9 (Ar*C*H), 122.4 (Ar*CH*), 121.3 (Ar*C*), 121.2 (Ar*C*H), 61.0 (O*C*H_3), 37.0 (N(*C*H_3)$_2$); **HRMS** (APCI): Calcd. for $C_{24}H_{22}NO_2S_2$ $[M+H]^+$: 420.1086; found: 420.1085.

***S*-(5-Bromo-2-(3-methoxybenzo[*b*]thiophen-2-yl)phenyl) dimethylcarbamothioate (5ac)**

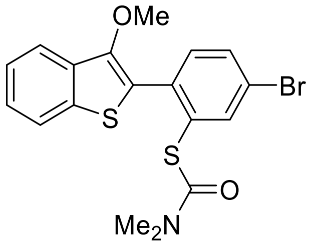

3ac (72 mg, 0.21 mmol), NaH (60%, 25 mg, 0.63 mmol), dimethylthiocarbamoyl chloride (45 mg, 0.42 mmol), DMF (2.1 mL), Ph_2O (2.1 mL), gave **5ac** as a white amorphous solid (75 mg, 0.18 mmol, 83%); **IR** (ATR): 1667, 1348, 1093 cm^{-1}; **^{1}H-NMR** (400 MHz, $CDCl_3$) δ: 7.85 (1H, d, *J* = 2.0 Hz, Ar*H*), 7.80 (1H, d, *J* = 8.0 Hz, Ar*H*), 7.74 (1H, d, *J* = 8.0 Hz, Ar*H*), 7.57 (1H, dd, *J* = 8.0, 2.0 Hz, Ar*H*), 7.47 (1H, d, *J* = 8.0 Hz, Ar*H*), 7.44-7.30 (2H, m, Ar*H*), 3.68 (3H, s, OCH_3), 2.96 (6H, s, N(CH_3)$_2$); **^{13}C-NMR** (101 MHz, $CDCl_3$) δ: 165.4 (*C*O), 147.9 (Ar*C*), 139.9 (Ar*C*H), 137.0 (Ar*C*), 136.3 (Ar*C*), 133.4 (Ar*C*), 133.3 (Ar*C*H), 132.14 (Ar*C*), 132.11 (Ar*C*H), 125.1 (Ar*C*H), 124.0 (Ar*C*H), 122.41 (Ar*C*), 122.35 (Ar*C*H), 121.3 (Ar*C*H), 120.3 (Ar*C*), 61.0 (O*C*H_3), 37.0 (N(*C*H_3)$_2$); **HRMS** (APCI): Calcd. for $C_{18}H_{17}NO_2BrS_2$ $[M+H]^+$: 421.9879; found: 421.9871.

***S*-(5-(*tert*-Butyl)-2-(3-methoxybenzo[*b*]thiophen-2-yl)phenyl) dimethylcarbamothioate (5ad)**

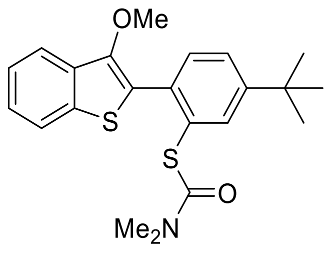

3ad (71 mg, 0.23 mmol), NaH (60%, 27 mg, 0.68 mmol), dimethylthiocarbamoyl chloride (49 mg, 0.45 mmol), DMF (2.3 mL), Ph_2O (2.3 mL), gave **5ad** as a white solid (65 mg, 0.16 mmol, 71%); **M.p.**: 165-166 °C; **IR** (ATR): 1672, 1258, 1090, 1015, 792 cm^{-1}; **^{1}H-NMR** (500 MHz, $CDCl_3$) δ: 7.70 (1H, d, *J* = 8.0 Hz, Ar*H*), 7.64 (1H, d, *J* = 8.0 Hz, Ar*H*), 7.59 (1H, s, Ar*H*), 7.45 (1H, d, *J* = 8.0 Hz, Ar*H*), 7.38 (1H, d, *J* = 8.0 Hz, Ar*H*), 7.29 (1H, t, *J* = 7.0 Hz, Ar*H*), 7.26 (1H, t, *J* = 7.0 Hz, Ar*H*), 3.60 (3H, s, OCH_3), 2.87 (6H, s, $N(CH_3)_2$), 1.30 (9H, s, $(CH_3)_3$); **^{13}C-NMR** (126 MHz, $CDCl_3$) δ: 166.3 (*C*O), 152.0 (Ar*C*), 147.5 (Ar*C*), 136.9 (Ar*C*), 134.8 (Ar*C*H), 134.4 (Ar*C*), 133.6 (Ar*C*), 131.9 (Ar*C*H), 129.4 (Ar*C*), 126.3 (Ar*C*H), 124.7 (Ar*C*H), 123.8 (Ar*C*H), 122.3 (Ar*C*H), 121.6 (Ar*C*), 121.1 (Ar*C*H), 60.9 (OCH_3), 37.0 ($N(CH_3)_2$), 34.7 ($C(CH_3)_3$), 31.2 ($C(CH_3)_3$); **HRMS** (APCI): Calcd. for $C_{22}H_{26}NO_2S_2$ $[M+H]^+$: 400.1399; found: 400.1391.

***S*-(3-(3-Methoxybenzo[*b*]thiophen-2-yl)-[1,1'-biphenyl]-4-yl) dimethylcarbamothioate (5ae)**

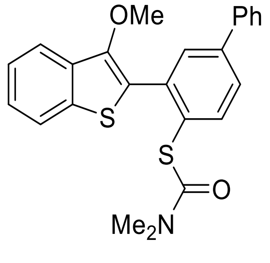

3ae (76 mg, 0.23 mmol), NaH (60%, 28 mg, 0.69 mmol), dimethylthiocarbamoyl chloride (49 mg, 0.46 mmol), DMF (2.3 mL), Ph_2O (2.0 mL), gave **5ae** as a colorless amorphous solid (96 mg, 0.23 mmol, quant.); **IR** (ATR): 1666, 1348, 1094, 762 cm^{-1}; **^{1}H-NMR** (500 MHz, $CDCl_3$) δ: 7.85

(1H, d, J = 3.0 Hz, ArH), 7.81 (1H, d, J = 8.0 Hz, ArH), 7.75 (1H, d, J = 8.0 Hz, ArH), 7.74 (1H, d, J = 8.0 Hz, ArH), 7.67 (1H, dd, J = 8.0, 3.0 Hz, ArH), 7.66-7.61 (2H, m, ArH), 7.48-7.43 (2H, m, ArH), 7.43-7.34 (3H, m, ArH), 3.72 (3H, s, OCH_3), 2.99 (6H, br s, N(CH_3)$_2$); **^{13}C-NMR** (126 MHz, $CDCl_3$) δ: 166.3 (*C*O), 147.7 (Ar*C*), 141.9 (Ar*C*), 139.7 (Ar*C*), 138.1 (Ar*C*H), 137.8 (Ar*C*), 137.0 (Ar*C*), 133.6 (Ar*C*), 130.8 (Ar*C*H), 129.1 (Ar*C*), 128.9 (Ar*C*H), 127.9 (Ar*C*H), 127.6 (Ar*C*H), 127.2 (Ar*C*H), 124.9 (Ar*C*H), 124.0 (Ar*C*H), 122.4 (Ar*C*H), 121.3 (Ar*C*H), 121.2 (Ar*C*), 61.0 (O*C*H$_3$), 37.0 (N(*C*H$_3$)$_2$); **HRMS** (APCI): Calcd. for $C_{24}H_{22}NO_2S_2$ [M+H]$^+$: 420.1086; found: 420.1070.

***S*-(4-(6-Bromo-3-methoxybenzo[*b*]thiophen-2-yl)-[1,1'-biphenyl]-3-yl) dimethylcarbamothioate (5bb)**

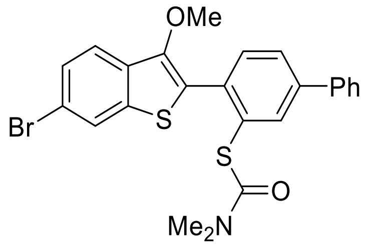

3bb (70 mg, 0.17 mmol), NaH (60%, 20 mg, 0.51 mmol), dimethylthiocarbamoyl chloride (37 mg, 0.34 mmol), DMF (1.7 mL), Ph_2O (1.7 mL), gave **5bb** as a colorless amorphous solid (55 mg, 0.11 mmol, 65%); **IR** (ATR): 1667, 1258, 1017, 791 cm^{-1}; **^{1}H-NMR** (400 MHz, $CDCl_3$) δ: 7.91 (1H, s, ArH), 7.88 (1H, s, ArH), 7.72-7.60 (5H, m, ArH), 7.53-7.42 (3H, m, ArH), 7.38 (1H, d, J = 7.3 Hz, ArH), 3.70 (3H, s, OCH_3), 2.98 (6H, s, N(CH_3)$_2$); **^{13}C-NMR** (126 MHz, $CDCl_3$) δ: 165.9 (*C*O), 147.5 (Ar*C*), 142.1 (Ar*C*), 139.6 (Ar*C*), 138.4 (Ar*C*), 136.4 (Ar*C*H), 135.7 (Ar*C*), 132.52 (Ar*C*H), 132.45 (Ar*C*), 130.6 (Ar*C*), 128.8 (Ar*C*H), 127.8 (Ar*C*H), 127.7 (Ar*C*H), 127.4 (Ar*C*H), 127.2 (Ar*C*H), 124.8 (Ar*C*H), 122.5 (Ar*C*H), 121.3 (Ar*C*), 118.8 (Ar*C*), 61.0 (O*C*H$_3$), 37.0 (N(*C*H$_3$)$_2$); **HRMS** (ESI): Calcd. for $C_{24}H_{20}BrNO_2S_2$ [M+Na]$^+$: 520.0011; found: 520.0002.

***S*-(2-(6-Bromo-3-methoxybenzo[*b*]thiophen-2-yl)-5-(*tert*-butyl)phenyl) dimethylcarbamothioate (5bd)**

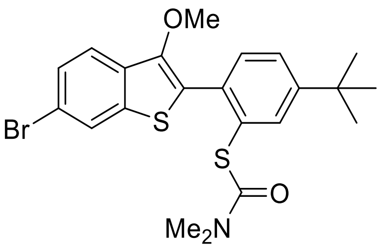

3bd (0.13 g, 0.32 mmol), NaH (60%, 39 mg, 0.97 mmol), dimethylthiocarbamoyl chloride (70 mg, 0.65 mmol), DMF (3.2 mL), Ph_2O (3.2 mL), gave **5bd** as an off-yellow amorphous solid (110 mg, 0.23 mmol, 71%); **IR** (ATR): 1667, 1347, 1080, 780 cm^{-1}; **^{1}H-NMR** (500 MHz, $CDCl_3$) δ: 7.86 (1H, s, Ar*H*), 7.66 (1H, s, Ar*H*), 7.63 (1H, d, *J* = 8.4 Hz, Ar*H*), 7.52 (1H, d, *J* = 8.4 Hz, Ar*H*), 7.49-7.42 (2H, m, Ar*H*), 3.65 (3H, s, OCH_3), 2.96 (6H, s, $N(CH_3)_2$), 1.37 (9H, s, $C(CH_3)_3$); **^{13}C-NMR** (126 MHz, $CDCl_3$) δ: 166.0 (*C*O), 152.3 (Ar*C*), 147.1 (Ar*C*), 138.2 (Ar*C*), 134.8 (Ar*C*H), 134.0 (Ar*C*), 132.4 (Ar*C*), 131.8 (Ar*C*H), 129.4 (Ar*C*), 127.2 (Ar*C*H), 126.3 (Ar*C*H), 124.7 (Ar*C*H), 122.3 (Ar*C*H), 121.6 (Ar*C*), 118.5 (Ar*C*), 60.8 (OCH_3), 36.9 ($N(CH_3)_2$), 34.7 ($C(CH_3)_3$), 31.1 ($C(CH_3)_3$); **HRMS** (ESI): Calcd. for $C_{22}H_{25}BrNO_2S_2$ $[M+H]^+$: 478.0505; found: 478.0507.

***S*-(2-(6-Bromo-3-methoxybenzo[*b*]thiophen-2-yl)-5-methoxyphenyl) dimethylcarbamothioate (5bf)**

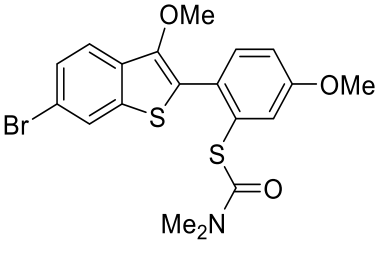

3bf (0.11 g, 0.30 mmol), NaH (60%, 36 mg, 0.90 mmol), dimethylthiocarbamoyl chloride (65 mg, 0.60 mmol), DMF (3.0 mL), Ph_2O (3.0 mL), gave **5bf** as a colorless amorphous solid (77 mg, 0.17 mmol, 57%); **IR** (ATR): 1670, 1257, 1036, 794 cm^{-1}; **^{1}H-NMR** (400 MHz, $CDCl_3$) δ: 7.84

(1H, s, Ar*H*), 7.62 (1H, d, *J* = 8.6 Hz, Ar*H*), 7.49 (1H, d, *J* = 8.8 Hz, Ar*H*), 7.47 (1H, d, *J* = 8.8 Hz, Ar*H*), 7.22 (1H, d, *J* = 2.2 Hz, Ar*H*), 6.99 (1H, dd, *J* = 8.8, 2.2 Hz, Ar*H*), 3.86 (3H, s, OCH_3), 3.64 (3H, s, OCH_3), 2.96 (6H, s, $N(CH_3)_2$); **^{13}C-NMR** (126 MHz, $CDCl_3$) δ: 165.9 (*C*O), 159.8 (Ar*C*), 147.2 (Ar*C*), 138.3 (Ar*C*), 133.0 (Ar*C*H), 132.5 (Ar*C*H), 131.3 (Ar*C*), 129.0 (Ar*C*), 127.3 (Ar*C*H), 124.8 (Ar*C*), 122.4 (Ar*C*H), 122.2 (Ar*C*H), 121.4 (Ar*C*), 118.6 (Ar*C*), 115.7 (Ar*C*H), 60.8 (OCH_3), 55.5 (OCH_3), 37.1 ($N(CH_3)_2$); **HRMS** (APCI): Calcd. for $C_{19}H_{19}BrNO_3S_2$ $[M+H]^+$: 451.9984; found: 451.9976.

S-(2-(6-Bromo-3-methoxybenzo[*b*]thiophen-2-yl)-5-(trifluoromethyl)phenyl) dimethylcarbamothioate (5bg)

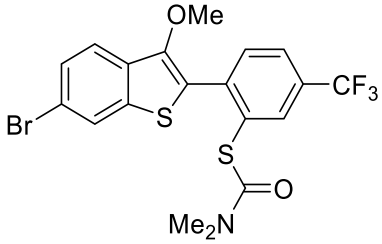

3bg (0.12 g, 0.30 mmol), NaH (60%, 36 mg, 0.90 mmol), dimethylthiocarbamoyl chloride (64 mg, 0.60 mmol), DMF (3.0 mL), Ph_2O (3.0 mL), gave **5bg** as yellow crystals (101 mg, 0.21 mmol, 69%); **M.p.**: 136-138 ºC; **IR** (ATR): 1675, 1317, 1122, 1075 cm^{-1}; **^{1}H-NMR** (400 MHz, $CDCl_3$) δ: 7.96 (1H, s, Ar*H*), 7.88 (1H, s, Ar*H*), 7.80-7.59 (3H, m, Ar*H*), 7.50 (1H, dd, *J* = 8.6, 1.2 Hz, Ar*H*), 3.66 (3H, s, OCH_3), 2.97 (6H, s, $N(CH_3)_2$); **^{13}C-NMR** (101 MHz, $CDCl_3$) δ: 165.0 (*C*O), 148.0 (Ar*C*), 140.7 (Ar*C*), 138.4 (Ar*C*), 134.5 (q, *J* = 3.9 Hz, Ar*C*H), 132.5 (Ar*C*H), 132.1 (Ar*C*), 131.5 (Ar*C*), 131.1 (q, *J* = 32.0 Hz, *C*CF_3), 127.6 (Ar*C*H), 125.7 (q, *J* = 3.6 Hz, Ar*C*H), 124.9 (Ar*C*H), 123.5 (q, *J* = 273.2 Hz, CCF_3), 122.6 (Ar*C*H), 120.2 (Ar*C*), 119.2 (Ar*C*), 61.2 (OCH_3), 37.0 ($N(CH_3)_2$); **HRMS** (ESI): Calcd. for $C_{19}H_{15}BrF_3NO_2S_2Na$ $[M+Na]^+$: 511.9572; found: 511.9560.

***S*-(5-(*tert*-Butyl)-2-(3-methoxy-6-phenylbenzo[*b*]thiophen-2-yl)phenyl) dimethylcarbamothioate (5cd)**

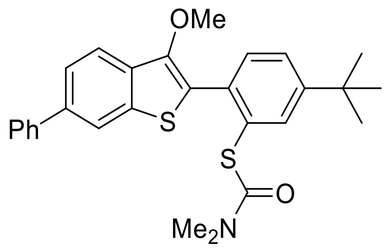

3cd (0.10 g, 0.26 mmol), NaH (60%, 31 mg, 0.77 mmol), dimethylthiocarbamoyl chloride (55 mg, 0.51 mmol), DMF (2.6 mL), Ph_2O (2.6 mL), gave **5cd** as a colorless amorphous solid (82 mg, 0.17 mmol, 67%); IR (ATR): 1660, 1258, 1016, 791 cm^{-1}; **^{1}H-NMR** (400 MHz, $CDCl_3$) δ: 7.95 (1H, s, Ar*H*), 7.85 (1H, d, *J* = 8.3 Hz, Ar*H*), 7.73-7.60 (4H, m, Ar*H*), 7.56 (1H, d, *J* = 8.1 Hz, Ar*H*), 7.52-7.43 (3H, m, Ar*H*), 7.37 (1H, t, *J* = 7.3 Hz, Ar*H*), 3.71 (3H, s, OCH_3), 2.98 (6H, s, $N(CH_3)_2$), 1.39 (9H, s, $C(CH_3)_3$); **^{13}C-NMR** (101 MHz, $CDCl_3$) δ: 166.3 (*C*O), 152.1 (Ar*C*), 147.4 (Ar*C*), 141.1 (Ar*C*), 138.1 (Ar*C*), 137.6 (Ar*C*), 134.9 (Ar*C*H), 134.4 (Ar*C*), 132.8 (Ar*C*), 131.9 (Ar*C*H), 129.4 (Ar*C*), 128.8 (Ar*C*H), 127.3 (Ar*C*H), 127.2 (Ar*C*H), 126.3 (Ar*C*H), 123.5 (Ar*C*H), 122.1 (Ar*C*), 121.4 (Ar*C*H), 120.7 (Ar*C*H), 60.9 (O*C*H_3), 37.0 (N(*C*H_3)$_2$), 34.8 (*C*(CH_3)$_3$), 31.2 (C(*C*H_3)$_3$); **HRMS** (APCI): Calcd. for $C_{28}H_{30}NO_2S_2$ $[M+H]^+$: 476.1712; found: 476.1713.

***S*-(3-(3-Methoxy-6-phenylbenzo[*b*]thiophen-2-yl)-[1,1'-biphenyl]-4-yl) dimethylcarbamothioate (5ce)**

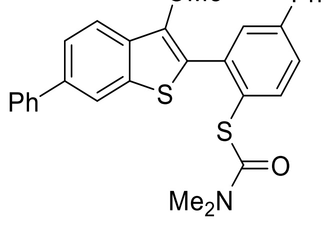

3ce (72 mg, 0.18 mmol), NaH (60%, 21 mg, 0.53 mmol), dimethylthiocarbamoyl chloride (38 mg, 0.35 mmol), DMF (1.8 mL), Ph_2O (1.8 mL), gave **5ce** as an off-yellow amorphous solid (63 mg,

0.13 mmol, 72%); **IR** (ATR): 1711, 1359, 1008, 787 cm^{-1}; **^{1}H-NMR** (400 MHz, $CDCl_3$) δ: 7.96 (1H, d, *J* = 1.0 Hz, Ar*H*), 7.87 (1H, d, *J* = 8.1 Hz, Ar*H*), 7.86 (1H, s, Ar*H*), 7.75 (1H, d, *J* = 8.3 Hz, Ar*H*), 7.71-7.61 (6H, m, Ar*H*), 7.52-7.42 (4H, m, Ar*H*), 7.41-7.33 (2H, m, Ar*H*), 3.74 (3H, s, OCH_3), 3.00 (6H, s, $N(CH_3)_2$); **^{13}C-NMR** (126 MHz, $CDCl_3$) δ: 166.4 (*C*O), 147.7 (Ar*C*), 142.0 (Ar*C*), 141.1 (Ar*C*), 139.8 (Ar*C*), 138.4 (Ar*C*), 138.1 (Ar*C*H), 137.9 (Ar*C*), 137.7 (Ar*C*), 132.8 (Ar*C*), 130.8 (Ar*C*H), 129.1 (Ar*C*), 128.92 (Ar*C*H), 128.89 (Ar*C*H), 127.9 (Ar*C*H), 127.7 (Ar*C*H), 127.4 (Ar*C*H), 127.34 (Ar*C*H), 127.26 (Ar*C*H), 123.7 (Ar*C*H), 121.7 (Ar*C*), 121.5 (Ar*C*H), 120.8 (Ar*C*H), 61.1 (OCH_3), 37.1 ($N(CH_3)_2$); **HRMS** (ESI): Calcd. for $C_{30}H_{25}NO_2S_2Na$ $[M+Na]^+$: 518.1230; found: 518.1211.

_S_-(4-(6-Decyl-3-methoxybenzo[_b_]thiophen-2-yl)-[1,1'-biphenyl]-3-yl) dimethylcarbamothioate (5eb)

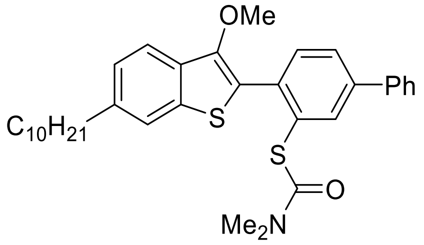

3eb (91 mg, 0.19 mmol), NaH (60%, 23 mg, 0.57 mmol), dimethylthiocarbamoyl chloride (41 mg, 0.38 mmol), DMF (1.9 mL), Ph_2O (1.9 mL), gave **5eb** as a colorless amorphous solid (69 mg, 0.12 mmol, 64%); **IR** (ATR): 1668, 1466, 1351, 1094 cm^{-1}; **^{1}H-NMR** (400 MHz, $CDCl_3$) δ: 7.92 (1H, s, Ar*H*), 7.71 (1H, d, *J* = 8.0 Hz, Ar*H*), 7.70-7.64 (4H, m, Ar*H*), 7.55 (1H, s, Ar*H*), 7.50-7.42 (2H, m, Ar*H*), 7.38 (1H, d, *J* = 8.0 Hz, Ar*H*), 7.23 (1H, dd, *J* = 8.0, 1.2 Hz, Ar*H*), 3.72 (3H, s, OCH_3), 2.98 (6H, s, $N(CH_3)_2$), 2.74 (2H, t, *J* = 7.7 Hz, $ArCH_2$), 1.81-1.62 (2H, m, CH_2), 1.47-1.15 (14H, m, CH_2), 0.89 (3H, t, *J* = 6.8 Hz, CH_2CH_3); **^{13}C-NMR** (101 MHz, $CDCl_3$) δ: (1 × CH_2 missing), 166.2 (*C*O), 147.7 (Ar*C*), 141.7 (Ar*C*), 140.2 (Ar*C*), 139.7 (Ar*C*), 137.3 (Ar*C*), 136.33 (Ar*C*), 136.28 (Ar*C*H), 132.6 (Ar*C*H), 131.6 (Ar*C*), 130.5 (Ar*C*), 128.8 (Ar*C*H), 127.7 (Ar*C*H),

127.6 (Ar*C*H), 127.2 (Ar*C*H), 125.1 (Ar*C*H), 121.6 (Ar*C*H), 120.9 (Ar*C*H), 120.2 (Ar*C*), 61.0 (O*C*H_3), 37.0 (N(*C*H_3)$_2$), 36.1 (*C*H_2), 31.9 (*C*H_2), 31.8 (*C*H_2), 29.6 (*C*H_2), 29.6 (*C*H_2), 29.5 (*C*H_2), 29.3 (*C*H_2), 22.7 (*C*H_2), 14.1 (*C*H_3); **HRMS** (APCI): Calcd. for $C_{34}H_{42}NO_2S_2$ $[M+H]^+$: 560.2651; found: 560.2640.

***S*-(5-Decyl-2-(3-methoxy-6-phenylbenzo[*b*]thiophen-2-yl)phenyl) dimethylcarbamothioate (5eb')**

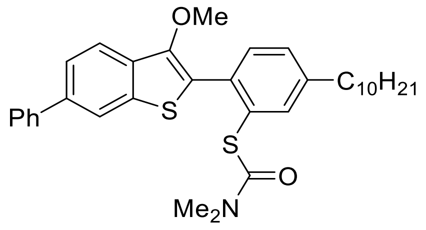

3eb' (142 mg, 0.30 mmol), NaH (60%, 36 mg, 0.90 mmol), dimethylthiocarbamoyl chloride (74 mg, 0.60 mmol), DMF (3.0 mL), Ph_2O (3.0 mL), gave **5eb'** as a colorless solid (117 mg, 0.21 mmol, 70%); **IR** (ATR): 1671, 1464, 1353, 1062 cm^{-1}; **^{1}H-NMR** (500 MHz, $CDCl_3$) δ: 7.93 (1H, dd, J = 1.6, 0.6 Hz, Ar*H*), 7.84 (1H, dd, J = 8.3, 0.6 Hz, Ar*H*), 7.70-7.64 (2H, m, Ar*H*), 7.62 (1H, dd, J = 8.3, 1.6 Hz, Ar*H*), 7.52 (1H, d, J = 8.2 Hz, Ar*H*), 7.50 (1H, d, J = 1.5 Hz, Ar*H*), 7.49 – 7.44 (2H, m, Ar*H*), 7.40-7.33 (1H, m, Ar*H*), 7.27 (1H, dd, J = 8.0, 1.9 Hz, Ar*H*), 3.69 (3H, s, OCH_3), 2.97 (6H, s, N(CH_3)$_2$), 2.67 (2H, t, J = 7.8 Hz, ArCH_2), 1.68 (2H, tt, J = 7.8, 7.8 Hz, CH_2), 1.46-1.20 (14H, m, CH_2), 0.89 (3H, t, J = 6.9 Hz, CH_2CH_3); **^{13}C-NMR** (126 MHz, $CDCl_3$) δ: 166.4 (*C*O), 147.4 (Ar*C*), 144.4 (Ar*C*), 141.1 (Ar*C*), 138.1 (Ar*C*), 137.63 (Ar*C*H), 137.60 (Ar*C*), 134.6 (Ar*C*), 132.8 (Ar*C*), 132.1 (Ar*C*H), 129.7 (Ar*C*), 129.3 (Ar*C*H), 128.8 (Ar*C*H), 127.35 (Ar*C*H), 127.26 (Ar*C*H), 123.6 (Ar*C*H), 122.0 (Ar*C*), 121.4 (Ar*C*H), 120.7 (Ar*C*H), 60.9 (O*C*H_3), 37.0 (N(*C*H_3)$_2$), 35.6 (*C*H_2), 31.9 (*C*H_2), 31.1 (*C*H_2), 29.62 (*C*H_2), 29.58 (*C*H_2), 29.5 (*C*H_2), 29.4 (*C*H_2), 29.3 (*C*H_2), 22.7 (*C*H_2), 14.1 (*C*H_3); **HRMS** (ESI): Calcd. for $C_{34}H_{41}NO_2S_2$ $[M+Na]^+$: 582.2471; found: 582.2445.

***S*-(2-(6-Decyl-3-methoxybenzo[*b*]thiophen-2-yl)-5-methoxyphenyl) dimethylcarbamothioate (5ef)**

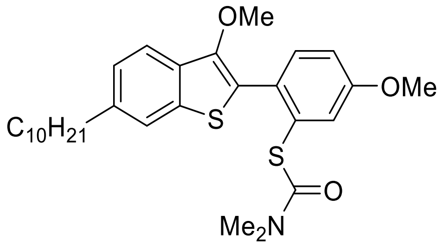

3ef (82 mg, 0.19 mmol), NaH (60%, 23 mg, 0.58 mmol), dimethylthiocarbamoyl chloride (41 mg, 0.38 mmol), DMF (1.9 mL), Ph_2O (1.9 mL), gave **5ef** as a colorless oil (74 mg, 0.14 mmol, 75%); **IR** (ATR): 1661, 1259, 1097, 1051, 1033 cm^{-1}; **^{1}H-NMR** (500 MHz, $CDCl_3$) δ: 7.68 (1H, d, *J* = 8.2 Hz, Ar*H*), 7.53 (1H, s, Ar*H*), 7.50 (1H, d, *J* = 8.2 Hz, Ar*H*), 7.23 (1H, d, *J* = 2.1 Hz, Ar*H*), 7.21 (1H, d, *J* = 8.2 Hz, Ar*H*), 6.99 (1H, dd, *J* = 8.2, 2.1 Hz, Ar*H*), 3.86 (3H, s, OCH_3), 3.67 (3H, s, OCH_3), 2.96 (6H, s, N(CH_3)$_2$), 2.73 (2H, t, *J* = 7.6 Hz, ArCH_2), 1.68 (2H, m, CH_2), 1.50-1.15 (14H, m, CH_2), 0.89 (3H, t, *J* = 6.7 Hz, CH_3); **^{13}C-NMR** (126 MHz, $CDCl_3$) δ: (1 × *C*H_2 missing), 166.1 (*C*O), 159.5 (Ar*C*), 147.4 (Ar*C*), 139.9 (Ar*C*), 137.1 (Ar*C*), 133.0 (Ar*C*H), 131.6 (Ar*C*), 131.2 (Ar*C*), 129.6 (Ar*C*), 125.0 (Ar*C*H), 122.0 (Ar*C*H), 121.5 (Ar*C*H), 120.8 (Ar*C*H), 120.1 (Ar*C*), 115.5 (Ar*C*H), 60.7 (O*C*H_3), 55.5 (O*C*H_3), 37.0 (N(*C*H_3)$_2$), 36.1 (*C*H_2), 31.9 (*C*H_2), 31.8 (*C*H_2), 29.6 (*C*H_2), 29. 6 (*C*H_2), 29.5 (*C*H_2), 29.3 (*C*H_2), 22.6 (*C*H_2), 14.1 (*C*H_3); **HRMS** (APCI): Calcd. for $C_{29}H_{40}NO_3S_2$ $[M+H]^+$: 514.2444; found: 514.2445.

***S*-(2-(6-Decyl-3-methoxybenzo[b]thiophen-2-yl)-5-(trifluoromethyl)phenyl) dimethylcarbamothioate (5eg)**

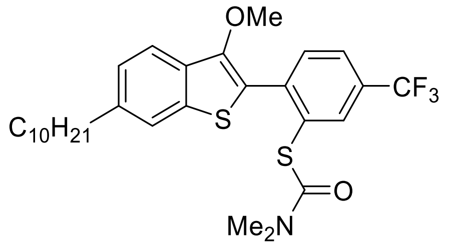

3eg (70 mg, 0.15 mmol), NaH (60%, 18 mg, 0.45 mmol), dimethylthiocarbamoyl chloride (32 mg, 0.30 mmol), DMF (1.5 mL), Ph_2O (1.5 mL), gave **5eg** as a colorless oil (66 mg, 0.12 mmol, 79%); **IR** (ATR): 1673, 1321, 1258 cm^{-1}: **^{1}H-NMR** (400 MHz, $CDCl_3$) δ: 7.96 (1H, s, Ar*H*), 7.76-7.69 (2H, m, Ar*H*), 7.67 (1H, d, *J* = 8.1 Hz, Ar*H*), 7.55 (1H, s, Ar*H*), 7.24 (1H, d, *J* = 8.1 Hz, Ar*H*), 3.68 (3H, s, OCH_3), 2.98 (6H, s, $N(CH_3)_2$), 2.73 (2H, t, *J* = 7.5 Hz, $ArCH_2$), 1.68 (2H, m, CH_2), 1.42-1.14 (14H, m, CH_2), 0.89 (3H, t, *J* = 7.6 Hz, CH_3); **^{13}C-NMR** (101 MHz, $CDCl_3$) δ: (1 × CH_2 missing), 165.3 (*C*O), 148.4 (Ar*C*), 141.3 (Ar*C*), 140.7 (Ar*C*), 137.4 (Ar*C*), 134.4 (q, *J* = 2.9 Hz, Ar*C*H), 132.6 (Ar*C*H), 131.5 (Ar*C*), 131.3 (Ar*C*), 130.7 (q, *J* = 33.1 Hz, CCF_3), 125.6 (q, *J* = 3.6 Hz, Ar*C*H), 125.3 (Ar*C*H), 123.6 (q, *J* = 271.3 Hz, CF_3), 121.7 (Ar*C*H), 121.1 (Ar*C*H), 119.2 (Ar*C*), 61.1 (OCH_3), 37.0 ($N(CH_3)_2$), 36.1 (CH_2), 31.9 (CH_2), 31.7 (CH_2), 29.62 (CH_2), 29.58 (CH_2), 29.5 (CH_2), 29.3 (CH_2), 22.7 (CH_2), 14.1 (CH_3); **HRMS** (APCI): Calcd. for $C_{29}H_{37}F_3NO_2S_2$ $[M+H]^+$: 522.2232; found: 522.2209.

***S*-(2-(6-Decyl-3-methoxybenzo[*b*]thiophen-2-yl)-5-(dimethylamino)phenyl) dimethylcarbamothioate (5eh)**

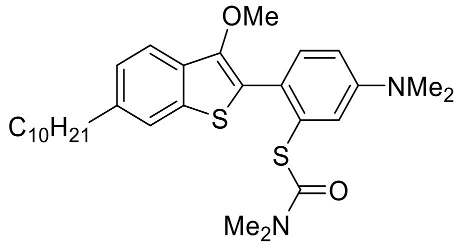

3eh (45 mg, 0.10 mmol), NaH (60%, 12 mg, 0.31 mmol), dimethylthiocarbamoyl chloride (22 mg, 0.20 mmol), DMF (1.0 mL), Ph_2O (1.0 mL), gave **5eh** as a colorless oil (15 mg, 0.028 mmol,

28%); **IR** (ATR): 1668, 1466, 1351, 1094 cm^{-1}; **^{1}H-NMR** (400 MHz, $CDCl_3$) δ: 7.65 (1H, d, *J* = 8.5 Hz, Ar*H*), 7.50 (1H, s, Ar*H*), 7.42 (1H, d, *J* = 8.5 Hz, Ar*H*), 7.18 (1H, d, *J* = 8.5 Hz, Ar*H*), 6.98 (1H, d, *J* = 2.0 Hz, Ar*H*), 6.77 (1H, dd, *J* = 8.5, 2.0 Hz, Ar*H*), 3.67 (3H, s, OCH_3), 3.01 (6H, s, $N(CH_3)_2$), 2.95 (6H, s, $N(CH_3)_2$), 2.71 (2H, t, *J* = 7.7 Hz, $ArCH_2$), 1.75-1.60 (2H, m, CH_2), 1.40-1.13 (14H, m, CH_2), 0.88 (3H, t, *J* = 6.6 Hz, CH_3); **^{13}C-NMR** (126 MHz, $CDCl_3$) δ: (1 × Ar*C*H missing) 166.7 (*C*O), 150.4 (Ar*C*), 147.1 (Ar*C*), 139.6 (Ar*C*), 137.0 (Ar*C*), 132.8 (Ar*C*H), 131.9 (Ar*C*), 130.2 (Ar*C*), 124.8 (Ar*C*H), 124.5 (Ar*C*), 121.5 (Ar*C*H), 121.2 (Ar*C*), 120.6 (Ar*C*H), 113.0 (Ar*C*H), 60.6 (OCH_3), 40.3 ($N(CH_3)_2$), 37.0 ($N(CH_3)_2$), 36.1 (CH_2), 31.9 (CH_2), 31.8 (CH_2), 29.62 (CH_2), 29.59 (CH_2), 29.5 (CH_2), 29.34 (CH_2), 29.33 (CH_2), 22.7 (CH_2), 14.1 (CH_3); **HRMS** (APCI): Calcd. for $C_{30}H_{43}N_2O_2S_2$ $[M+H]^+$: 527.2760; found: 527.2757.

[1]Benzothieno[3,2-*b*][1]benzothiophene (4aa)[2]

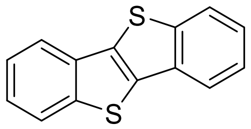

5aa (18 mg, 0.052 mmol), KOMe (90%, 8 mg, 0.10 mmol), MeOH (1.0 mL), toluene (1.0 mL), TsOH·H_2O (79 mg, 0.42 mmol, 8.0 equiv), gave **4aa** as a white solid (8 mg, 0.033 mmol, 70%); **M.p.**: 217-218 °C; **IR** (ATR): 1436, 1335, 739 cm^{-1}; **^{1}H-NMR** (500 MHz, $CDCl_3$) δ: 7.93 (2H, d, J = 7.8 Hz, Ar*H*), 7.90 (2H, d, J = 7.8 Hz, Ar*H*), 7.47 (2H, t, J = 7.8 Hz, Ar*H*), 7.41 (2H, t, J = 7.8 Hz, Ar*H*); **^{13}C-NMR** (126 MHz, $CDCl_3$) δ: 142.2 (Ar*C*), 133.4 (Ar*C*), 133.1 (Ar*C*), 125.0 (Ar*C*H), 124.9 (Ar*C*H), 124.0 (Ar*C*H), 121.6 (Ar*C*H); **HRMS** (APCI): Calcd. for $C_{14}H_9S_2$ $[M+H]^+$: 241.0140; found: 241.0140.

2-Phenyl[1]benzothieno[3,2-*b*][1]benzothiophene (4ab)[3]

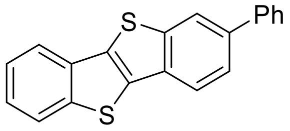

5ab (42 mg, 0.10 mmol), KOMe (90%, 16 mg, 0.20 mmol), MeOH (1.0 mL), toluene (1.0 mL), TsOH·H_2O (0.15 g, 0.80 mmol, 8.0 equiv), gave **4ab** as a white solid (25 mg, 0.079 mmol, 77%); **M.p.**: 242 °C; IR (ATR): 1258, 1016, 790 cm^{-1}; **^{1}H-NMR** (400 MHz, $CDCl_3$) δ: 8.14 (1H, s, Ar*H*), 7.99-7.92 (2H, m, Ar*H*), 7.90 (1H, d, J = 7.8 Hz, Ar*H*), 7.78-7.63 (3H, m, Ar*H*), 7.55-7.45 (3H, m, Ar*H*), 7.45-7.35 (2H, m, Ar*H*); **^{13}C-NMR** (126 MHz, $CDCl_3$) δ: 143.1 (Ar*C*), 142.3 (Ar*C*), 140.7 (Ar*C*), 138.4 (Ar*C*), 133.7 (Ar*C*), 133.2 (Ar*C*), 133.1 (Ar*C*), 132.2 (Ar*C*), 128.9 (Ar*C*H), 127.5 (Ar*C*H), 127.3 (Ar*C*H), 125.0 (Ar*C*H), 124.9 (Ar*C*H), 124.5 (Ar*C*H), 124.1 (Ar*C*H), 122.4 (Ar*C*H), 121.8 (Ar*C*H), 121.6 (Ar*C*H); **HRMS** (APCI): Calcd. for $C_{20}H_{13}S_2$ $[M+H]^+$: 317.0453;

---

[2] T. Kitamura, K. Morita, H. Nakamori and J. Oyamada, *J. Org. Chem.* 2019, **84**, 4191–4199.

[3] A. Sanzone, S. Mattiello, G. M. Garavaglia, A. M. Calascibetta, C. Ceriani, M. Sassi and L. Beverina, *Green Chem.* 2019, **21**, 4400–4405.

found: 317.0450.

2-Bromo[1]benzothieno[3,2-*b*][1]benzothiophene (4ac)[2]

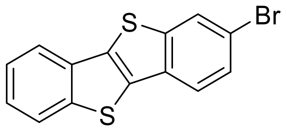

5ac (75 mg, 0.18 mmol), KOMe (90%, 28 mg, 0.36 mmol), MeOH (1.8 mL), toluene (1.8 mL), TsOH·H_2O (0.27 g, 1.4 mmol, 8.0 equiv), gave **4ac** as a white solid (35 mg, 0.11 mmol, 62%); **M.p.**: 214-216 ºC; **IR** (ATR): 1258, 1085, 1029, 808 cm^{-1}; **^{1}H-NMR** (400 MHz, $CDCl_3$) δ: 8.05 (1H, s, Ar*H*), 7.92 (1H, d, *J* = 7.8 Hz, Ar*H*), 7.87 (1H, d, *J* = 7.8 Hz, Ar*H*), 7.73 (1H, d, *J* = 8.6 Hz, Ar*H*), 7.56 (1H, d, *J* = 8.6 Hz, Ar*H*), 7.47 (1H, t, *J* = 7.8 Hz, Ar*H*), 7.42 (1H, t, *J* = 7.8 Hz, Ar*H*); **^{13}C-NMR** (101 MHz, $CDCl_3$) δ: 143.6 (Ar*C*), 142.3 (Ar*C*), 133.7 (Ar*C*), 123.9 (Ar*C*), 132.8 (Ar*C*), 131.9 (Ar*C*), 128.3 (Ar*C*H), 126.5 (Ar*C*H), 125.3 (Ar*C*H), 125.0 (Ar*C*H), 124.1 (Ar*C*H), 122.5 (Ar*C*H), 121.6 (Ar*C*H), 118.5 (Ar*C*); **HRMS** (APCI): Calcd. for $C_{14}H_7BrS_2$ $[M]^+$: 317.9167; found: 317.9170.

2- *tert*-Butyl[1]benzothieno[3,2-*b*][1]benzothiophene (4ad)[4]

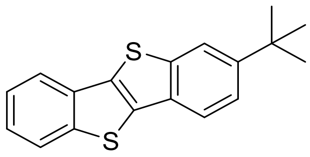

5ad (61 mg, 0.15 mmol), KOMe (90%, 24 mg, 0.31 mmol), MeOH (1.5 mL), toluene (1.5 mL), TsOH·H_2O (0.23 g, 1.2 mmol, 8.0 equiv), gave **4ad** as a white solid (40 mg, 0.13 mmol, 88%); **M.p.**: 175-177 ºC; **IR** (ATR): 1253, 816, 745 cm^{-1}; **^{1}H-NMR** (400 MHz, $CDCl_3$) δ: 7.95-7.89

---

[4] G. Schweicher, V. Lemaur, C. Niebel, C. Ruzié, Y. Diao, O. Goto, W. Lee, Y. Kim, J. Arlin, J. Karpinska, A. R. Kennedy, S. R. Parkin, Y. Olivier, S. C. B. Mannsfeld, J. Cornil, Y. H. Geerts and Z. Bao, *Adv. Mater.*, 2015, **27**, 3066–3072.

(2H, m, Ar*H*), 7.88 (1H, d, *J* = 8.0 Hz, Ar*H*), 7.82 (1H, d, *J* = 8.0 Hz, Ar*H*), 7.52 (1H, d, *J* = 8.0 Hz, Ar*H*), 7.45 (1H, t, *J* = 8.0 Hz, Ar*H*), 7.39 (1H, t, *J* = 8.0 Hz, Ar*H*), 1.42 (9H, s, (CH_3)$_3$); **^{13}C-NMR** (126 MHz, $CDCl_3$) δ: 148.6 (Ar*C*), 142.6 (Ar*C*), 142.1 (Ar*C*), 133.3 (Ar*C*), 133.2 (Ar*C*), 132.8 (Ar*C*), 130.7 (Ar*C*), 124.8 (Ar*C*H), 124.7 (Ar*C*H), 124.0 (Ar*C*H), 123.8 (Ar*C*H), 121.4 (Ar*C*H), 121.1 (Ar*C*H), 120.3 (Ar*C*H), 35.1 (*C*(CH_3)$_3$), 31.5 (C(*C*H_3)$_3$); **HRMS** (APCI): Calcd. for $C_{18}H_{17}S_2$ [M+H]$^+$: 297.0766; found: 297.0763.

3-Phenyl[1]benzothieno[3,2-*b*][1]benzothiophene (4ae)

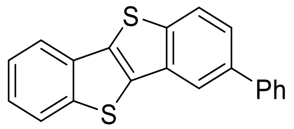

5ae (60 mg, 0.14 mmol), KOMe (90%, 22 mg, 0.29 mmol), MeOH (1.4 mL), toluene (1.4 mL), TsOH·H_2O (0.22 g, 1.1 mmol, 8.0 equiv), gave **4ae** as a white solid (33 mg, 0.10 mmol, 77%); **M.p.**: 168-170 °C; **IR** (ATR): 1437, 1260, 1016, 751 cm^{-1}; **^{1}H-NMR** (400 MHz, $CDCl_3$) δ: 8.08 (1H, d, *J* = 1.5 Hz, Ar*H*), 7.98 (1H, d, *J* = 8.3 Hz, Ar*H*), 7.94 (1H, d, *J* = 7.8 Hz, Ar*H*), 7.91 (1H, d, *J* = 7.8 Hz, Ar*H*), 7.76-7.68 (2H, m, Ar*H*), 7.66 (1H, dd, *J* = 8.3, 1.7 Hz, Ar*H*), 7.55-7.45 (2H, m, Ar*H*), 7.45 (1H, dd, *J* = 7.1, 1.2 Hz, Ar*H*), 7.45-7.36 (2H, m, Ar*H*). **^{13}C-NMR** (126 MHz, $CDCl_3$) δ: (1 × Ar*C*H missing), 142.2 (Ar*C*), 141.2 (Ar*C*), 140.9 (Ar*C*), 138.5 (Ar*C*), 134.0 (Ar*C*), 133.6 (Ar*C*), 133.5 (Ar*C*), 133.1 (Ar*C*), 128.9 (Ar*C*H), 127.4 (Ar*C*H), 125.1 (Ar*C*H), 124.9 (Ar*C*H), 124.5 (Ar*C*H), 124.2 (Ar*C*H), 124.1 (Ar*C*H), 121.6 (Ar*C*H), 120.0 (Ar*C*H); **HRMS** (APCI): Calcd. for $C_{20}H_{13}S_2$ [M+H]$^+$: 317.0453; found: 317.0450.

2-Bromo-7-phenyl[1]benzothieno[3,2-*b*][1]benzothiophene (4bb)

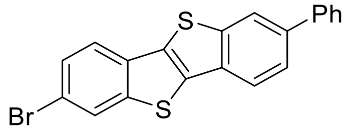

5bb (87 mg, 0.17 mmol), KOMe (90%, 27 mg, 0.35 mmol), MeOH (1.7 mL), toluene (1.7 mL),

$TsOH \cdot H_2O$ (0.24 g, 1.4 mmol, 8.0 equiv), gave **4bb** as a white solid (45 mg, 0.11 mmol, 65%); **M.p.**: 294-296 ℃; **IR** (ATR): 810, 759, 690 cm^{-1}; **^{1}H-NMR** (400 MHz, $CDCl_3$) δ: 8.13 (1H, s, Ar*H*), 8.07 (1H, s, Ar*H*), 7.93 (1H, d, *J* = 8.3 Hz, Ar*H*), 7.78-7.71 (2H, m, Ar*H*), 7.71-7.66 (2H, m, Ar*H*), 7.57 (1H, d, *J* = 8.3 Hz, Ar*H*), 7.53-7.45 (2H, m, Ar*H*), 7.41 (1H, d, *J* = 8.3 Hz, Ar*H*); **^{13}C-NMR** (126 MHz, $CDCl_3$) δ: 143.7 (Ar*C*), 153.1 (Ar*C*), 140.6 (Ar*C*), 138.7 (Ar*C*), 133.6 (Ar*C*), 133.2 (Ar*C*), 131.94 (Ar*C*), 131.90 (Ar*C*), 129.0 (Ar*C*H), 128.3 (Ar*C*H), 127.6 (Ar*C*H), 127.3 (Ar*C*H), 126.5 (Ar*C*H), 124.7 (Ar*C*H), 122.5 (Ar*C*H), 122.4 (Ar*C*H), 121.8 (Ar*C*H), 118.6 (Ar*C*); **HRMS** (APCI): Calcd. for $C_{20}H_{12}BrS_2$ $[M+H]^+$: 394.9558; found: 394.9546.

2-Bromo-7-*tert*-butyl[1]benzothieno[3,2-*b*][1]benzothiophene (4bd)

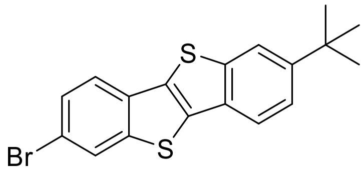

5bd (105 mg, 0.22 mmol), KOMe (90%, 34 mg, 0.44 mmol), MeOH (1.1 mL), toluene (1.1 mL), $TsOH \cdot H_2O$ (0.33 g, 1.8 mmol, 8.0 equiv), gave **4bd** as a white solid (40 mg, 0.11 mmol, 49%); **M.p.**: 278-280 ℃; **IR** (ATR): 1257, 1012, 787 cm^{-1}; **^{1}H-NMR** (500 MHz, $CDCl_3$) δ: 8.05 (1H, s, Ar*H*), 7.91 (1H, s, Ar*H*), 7.80 (1H, d, *J* = 8.4 Hz, Ar*H*), 7.73 (1H, d, *J* = 8.4 Hz, Ar*H*), 7.55 (1H, d, *J* = 8.4 Hz, Ar*H*), 7.54 (1H, d, *J* = 8.4 Hz, Ar*H*), 1.42 (9H, s, $C(CH_3)_3$); **^{13}C-NMR** (126 MHz, $CDCl_3$) δ: 149.0 (Ar*C*), 143.5 (Ar*C*), 142.6 (Ar*C*), 133.6 (Ar*C*), 132.4 (Ar*C*), 132.1 (Ar*C*), 130.5 (Ar*C*), 128.2 (Ar*C*H), 126.5 (Ar*C*H), 123.3 (Ar*C*H), 122.4 (Ar*C*H), 121.1 (Ar*C*H), 120.3 (Ar*C*H), 118.2 (Ar*C*), 35.2 ($C(CH_3)_3$), 31.5 ($C(CH_3)_3$); **HRMS** (ESI): Calcd. for $C_{18}H_{16}BrS_2$ $[M+H]^+$: 374.9871; found: 374.9872.

2-Bromo-7-methoxy[1]benzothieno[3,2-*b*][1]benzothiophene (4bf)

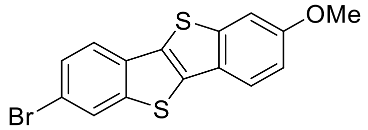

5bf (70 mg, 0.16 mmol), KOMe (90%, 24 mg, 0.31 mmol), MeOH (1.5 mL), toluene (1.5 mL), TsOH·H_2O (0.19 g, 1.1 mmol, 7.0 equiv), gave **4bf** as a white solid (40 mg, 0.11 mmol, 74%); **M.p.**: 200-202 ºC; **IR** (ATR): 1259, 1021, 795 cm^{-1}; **^{1}H-NMR** (400 MHz, $CDCl_3$) δ: 8.03 (1H, d, *J* = 2.0 Hz, Ar*H*), 7.75 (1H, d, *J* = 8.6 Hz, Ar*H*), 7.68 (1H, d, *J* = 8.6 Hz, Ar*H*), 7.54 (1H, dd, *J* = 8.6, 2.0 Hz, Ar*H*), 7.39 (1H, d, *J* = 2.0 Hz, Ar*H*), 7.07 (1H, dd, *J* = 8.6, 2.0 Hz, Ar*H*), 3.92 (3H, s, OC*H*$_3$); **^{13}C-NMR** (126 MHz, $CDCl_3$) δ: 158.1 (Ar*C*), 144.1 (Ar*C*), 143.2 (Ar*C*), 133.7 (Ar*C*), 132.2 (Ar*C*), 130.8 (Ar*C*), 128.2 (Ar*C*H), 126.8 (Ar*C*), 126.4 (Ar*C*H), 122.3 (Ar*C*H), 122.1 (Ar*C*H), 117.9 (Ar*C*), 114.6 (Ar*C*H), 107.0 (Ar*C*H), 55.7 (O*C*H$_3$); **HRMS** (APCI): Calcd. for $C_{15}H_{10}BrOS_2$ $[M+H]^+$: 348.9351; found: 348.9344.

2-Bromo-7-trifluoromethyl[1]benzothieno[3,2-*b*][1]benzothiophene (4bg)

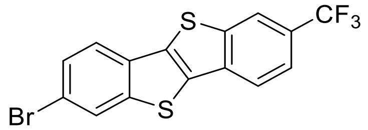

5bg (97 mg, 0.20 mmol), KOMe (90%, 31 mg, 0.40 mmol), MeOH (2.0 mL), toluene (2.0 mL), TsOH·H_2O (0.26 g, 1.6 mmol, 8.0 equiv), gave **4bg** as a white solid (36 mg, 0.093 mmol, 47%); **M.p.**: 255-257 ºC; **IR** (ATR): 1317, 1168, 1109, 806 cm^{-1}; **^{1}H-NMR** (400 MHz, $CDCl_3$) δ: 8.21 (1H, s, Ar*H*), 8.09 (1H, d, *J* = 1.2 Hz, Ar*H*), 7.97 (1H, d, *J* = 8.6 Hz, Ar*H*), 7.79 (1H, d, *J* = 8.6 Hz, Ar*H*), 7.70 (1H, d, *J* = 8.6 Hz, Ar*H*), 7.60 (1H, dd, *J* = 8.6, 1.2 Hz, Ar*H*); **^{13}C-NMR** (126 MHz, $CDCl_3$) δ: 144.1 (Ar*C*), 142.1 (Ar*C*), 135.6 (Ar*C*), 135.2 (Ar*C*), 133.1 (Ar*C*), 131.5 (Ar*C*), 128.6 (Ar*C*H), 127.3 (q, *J* = 32.0 Hz, *C*CF$_3$), 126.7 (Ar*C*H), 124.2 (q, *J* = 272.8 Hz, C*C*F$_3$), 123.0 (Ar*C*H), 122.0 (q, *J* = 4.2 Hz, Ar*C*H), 121.9 (Ar*C*), 121.5 (q, *J* = 3.9 Hz, Ar*C*H), 119.6 (Ar*C*H); **HRMS** (APCI): Calcd. for $C_{15}H_6BrF_3S_2$ $[M]^+$: 385.9041; found: 385.9036.

2- *tert*-Butyl-7-phenyl[1]benzothieno[3,2-*b*][1]benzothiophene (4cd)

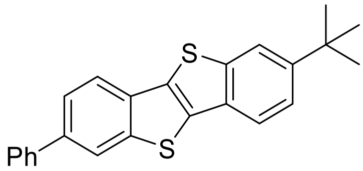

5cd (44 mg, 0.093 mmol), KOMe (90%, 14 mg, 0.19 mmol), MeOH (1.0 mL), toluene (1.0 mL), TsOH·H_2O (0.11 g, 0.65 mmol; 7.0 equiv), gave **4cd** as a white solid (21 mg, 0.056 mmol, 61%); **M.p.**: 268-270 ºC; **IR** (ATR): 818, 760, 690 cm^{-1}; **^{1}H-NMR** (500 MHz, $CDCl_3$) δ: 8.14 (1H, d, *J* = 1.5 Hz, Ar*H*), 7.99-7.91 (2H, m, Ar*H*), 7.85 (1H, d, *J* = 8.4 Hz, Ar*H*), 7.75-7.66 (3H, m, Ar*H*), 7.55 (1H, dd, *J* = 8.4, 1.5 Hz, Ar*H*), 7.53-7.47 (2H, m, Ar*H*), 7.40 (1H, t, *J* = 8.4 Hz, Ar*H*), 1.45 (9H, s, C(C*H*$_3$)$_3$); **^{13}C-NMR** (126 MHz, $CDCl_3$) δ: 148.7 (Ar*C*), 142.9 (Ar*C*), 142.6 (Ar*C*), 140.8 (Ar*C*), 138.1 (Ar*C*), 133.5 (Ar*C*), 132.6 (Ar*C*), 132.3 (Ar*C*), 130.8 (Ar*C*), 128.9 (Ar*C*H), 127.4 (Ar*C*H), 127.3 (Ar*C*H), 124.4 (Ar*C*H), 123.1 (Ar*C*H), 122.3 (Ar*C*H), 121.6 (Ar*C*H), 121.1 (Ar*C*H), 120.3 (Ar*C*H), 35.1 (*C*(CH_3)$_3$), 31.5 (C(*C*H$_3$)$_3$); **HRMS** (APCI): Calcd. for $C_{24}H_{21}S_2$ $[M+H]^+$: 373.1079; found: 373.1064.

2,8-Diphenyl[1]benzothieno[3,2-*b*][1]benzothiophene (4ce)

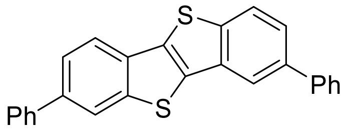

5ce (60 mg, 0.12 mmol), KOMe (90%, 19 mg, 0.24 mmol), MeOH (1.2 mL), toluene (1.2 mL), TsOH·H_2O (0.18 g, 0.96 mmol, 8.0 equiv), gave **4ce** as a white solid (30 mg, 0.076 mmol, 63%); **M.p.**: 222-224 ºC; **IR** (ATR): 1257, 1012, 787 cm^{-1}; **^{1}H-NMR** (500 MHz, $CDCl_3$) δ: 8.15 (1H, d, *J* = 1.5 Hz, Ar*H*), 8.09 (1H, d, *J* = 1.5 Hz, Ar*H*), 7.99 (1H, d, *J* = 8.4 Hz, Ar*H*), 7.96 (1H, d, *J* = 8.4 Hz, Ar*H*), 7.77-7.69 (5H, m, Ar*H*), 7.66 (1H, dd, *J* = 8.4, 1.5 Hz, Ar*H*), 7.54-7.45 (4H, m, Ar*H*), 7.45-7.35 (2H, m, Ar*H*); **^{13}C-NMR** (126 MHz, $CDCl_3$) δ: (2 × ArCH missing), 143.1 (Ar*C*), 141.3 (Ar*C*), 140.9 (Ar*C*), 140.7 (Ar*C*), 138.6 (Ar*C*), 138.5 (Ar*C*), 133.80 (Ar*C*), 133.79 (Ar*C*),

133.7 (Ar*C*), 132.2 (Ar*C*), 128.93 (Ar*C*H), 128.91 (Ar*C*H), 127.5 (Ar*C*H), 127.4 (Ar*C*H), 127.3 (Ar*C*H), 124.6 (Ar*C*H), 124.3 (Ar*C*H), 122.4 (Ar*C*H), 121.8 (Ar*C*H), 120.0 (Ar*C*H); **HRMS** (APCI): Calcd. for $C_{26}H_{17}S_2$ $[M+H]^+$: 393.0766; found: 393.0759.

2-Decyl-7-phenyl[1]benzothieno[3,2-*b*][1]benzothiophene (4eb)[5]

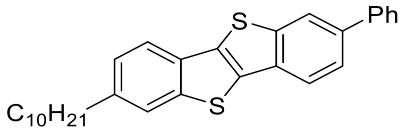

5eb (53 mg, 0.095 mmol), KOMe (90%, 15 mg, 0.19 mmol), MeOH (1.0 mL), toluene (1.0 mL), TsOH·H_2O (0.11 g, 0.59 mmol, 6.0 equiv), gave **4eb** as a white solid (30 mg, 0.066 mmol, 69%); **IR** (ATR): 1258, 1014, 798 cm^{-1}; **^{1}H-NMR** (500 MHz, $CDCl_3$) δ: 8.12 (1H, d, *J* = 1.2 Hz, Ar*H*), 7.92 (1H, d, *J* = 8.2 Hz, Ar*H*), 7.80 (1H, d, *J* = 8.2 Hz, Ar*H*), 7.73 (1H, s, Ar*H*), 7.72-7.66 (3H, m, Ar*H*), 7.45-7.54 (2H, m, Ar*H*), 7.38 (1H, d, *J* = 7.8 Hz, Ar*H*), 7.29 (1H, dd, *J* = 7.8, 1.2 Hz, Ar*H*), 2.77 (2H, t, *J* = 7.8 Hz, ArCH_2), 1.71 (2H, tt, *J* = 7.8, 7.8 Hz, CH_2), 1.44-1.18 (14H, m, CH_2), 0.88 (3H, t, *J* = 6.7 Hz, CH_3); **^{13}C-NMR** (101 MHz, $CDCl_3$) δ: (1 × Ar*C* missing) 142.9 (Ar*C*), 142.6 (Ar*C*), 140.8 (Ar*C*), 140.5 (Ar*C*), 138.1 (Ar*C*), 133.7 (Ar*C*), 132.3 (Ar*C*), 131.0 (Ar*C*), 128.9 (Ar*C*H), 127.4 (Ar*C*H), 127.3 (Ar*C*H), 126.0 (Ar*C*H), 124.4 (Ar*C*H), 123.4 (Ar*C*H), 122.3 (Ar*C*H), 121.6 (Ar*C*H), 121.2 (Ar*C*H), 36.1 (CH_2), 31.9 (CH_2), 31.7 (CH_2), 29.6 (CH_2), 29.6 (CH_2), 29.5 (CH_2), 29.32 (CH_2), 29.30 (CH_2), 22.7 (CH_2), 14.1 (CH_3); **HRMS** (APCI): Calcd. for $C_{30}H_{33}S_2$ $[M+H]^+$: 457.2018; found: 457.2009.

From **5eb'**: **5eb'** (56 mg, 0.1 mmol), KOMe (90%, 16 mg, 0.2 mmol), MeOH (1.0 mL), toluene (1.0 mL), TsOH·H_2O (0.15 g, 0.8 mmol, 8.0 equiv), gave **4eb** as a white solid (41 mg, 0.091 mmol, 91%).

---

[5] H. Iino, T. Usui and J. Hanna, *Nat. Commun.* 2015, **6**, 6828.

2-Decyl-7-methoxy[1]benzothieno[3,2-*b*][1]benzothiophene (4ef)

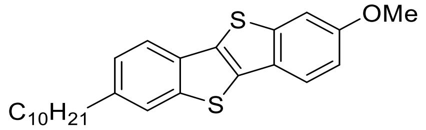

5ef (68 mg, 0.13 mmol), KOMe (90%, 20 mg, 0.26 mmol), MeOH (1.3 mL), toluene (1.3 mL), TsOH·H_2O (0.20 g, 1.1 mmol, 8.0 equiv), gave **4ef** as a white solid (41 mg, 0.10 mmol, 76%); **M.p.**:138-140 ºC; **IR** (ATR): 1259, 1030, 810, 789 cm^{-1}; **^{1}H-NMR** (500 MHz, $CDCl_3$) δ: 7.74 (1H, d, J = 4.0 Hz, Ar*H*), 7.72 (1H, d, J = 4.0 Hz, Ar*H*), 7.69 (1H, s, Ar*H*), 7.39 (1H, s, Ar*H*), 7.26 (1H, d, J = 8.0 Hz, Ar*H*), 7.06 (1H, d, J = 8.0 Hz, Ar*H*), 3.91 (3H, s, OCH_3), 2.75 (2H, t, J = 7.7 Hz, ArCH_2), 1.76-1.63 (2H, m, CH_2), 1.47-1.19 (14H, m, CH_2), 0.88 (3H, t, J = 6.7 Hz, CH_3); **^{13}C-NMR** (126 MHz, $CDCl_3$) δ: 157.7 (Ar*C*OCH_3), 143.7 (Ar*C*), 142.1 (Ar*C*), 139.7 (Ar*C*), 132.4 (Ar*C*), 131.3 (Ar*C*), 131.2 (Ar*C*), 127.2 (Ar*C*), 125.8 (Ar*C*H), 123.3 (Ar*C*H), 122.0 (Ar*C*H), 120.8 (Ar*C*H), 114.2 (Ar*C*H), 107.0 (Ar*C*H), 55.7 (OCH_3), 36.1 (CH_2), 31.9 (CH_2), 31.7 (CH_2), 29.6 (CH_2), 29.6 (CH_2), 29.5 (CH_2), 29.3 (CH_2), 29.3 (CH_2), 22.7 (CH_2), 14.1 (CH_3); **HRMS** (APCI): Calcd. for $C_{25}H_{31}OS_2$ $[M+H]^+$: 411.1811; found: 411.1807.

2-Decyl-7-trifluoromethyl[1]benzothieno[3,2-*b*][1]benzothiophene (4eg)

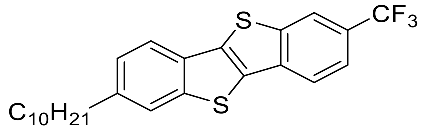

5eg (44 mg, 0.080 mmol), KOMe (90%, 12 mg, 0.16 mmol), MeOH (0.8 mL), toluene (0.8 mL), TsOH·H_2O (0.12 g, 0.64 mmol, 8.0 equiv), gave **4eg** as a white solid (34 mg, 0.076 mmol, 96%); **M.p.**: 210-212; **IR** (ATR): 1319, 1119, 1084, 817 cm^{-1}; **^{1}H-NMR** (500 MHz, $CDCl_3$) δ: 8.18 (1H, s, Ar*H*), 7.93 (1H, d, J = 8.0 Hz, Ar*H*), 7.82 (1H, d, J = 8.0 Hz, Ar*H*), 7.74 (1H, s, Ar*H*), 7.67 (1H, d, J = 8.0 Hz, Ar*H*), 7.31 (1H, d, J = 8.0 Hz, Ar*H*), 2.77 (2H, t, J = 7.0 Hz, ArCH_2), 1.70 (2H, m, CH_2), 1.42-1.17 (14H, m, CH_2), 0.88 (3H, t, J = 7.0 Hz, CH_3); **^{13}C-NMR** (126 MHz, $CDCl_3$) δ: 143.0 (Ar*C*), 141.8 (Ar*C*), 141.4 (Ar*C*), 136.0 (Ar*C*), 135.6 (Ar*C*), 131.9 (Ar*C*), 130.6

(ArC), 126.6 (q, J = 33.0 Hz, CCF$_3$), 126.2 (ArCH), 124.4 (q, J = 270.2 Hz, CF$_3$), 123.4 (ArCH), 121.7 (q, J = 3.6 Hz, ArCH), 121.6 (ArCH), 121.5 (ArCH), 121.1 (J = 4.2 Hz, ArCH), 36.2 (CH$_2$), 31.9 (CH$_2$), 31.6 (CH$_2$), 29.6 (CH$_2$), 29.6 (CH$_2$), 29.5 (CH$_2$), 29.3 (CH$_2$), 29.3 (CH$_2$), 22.7 (CH$_2$), 14.1 (CH$_3$); **HRMS** (APCI): Calcd. for $C_{25}H_{27}F_3S_2$ $[M]^+$: 448.1501; found: 448.1498.

2-Decyl-7-dimethylamino[1]benzothieno[3,2-*b*][1]benzothiophene (4eh)

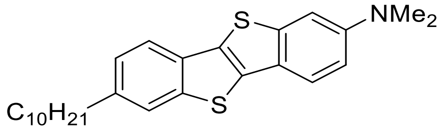

5eh (25 mg, 0.047 mmol), KOMe (90%, 7 mg, 0.095 mmol), MeOH (1.0 mL), toluene (1.0 mL), TsOH·H_2O (72 mg, 0.38 mmol, 8.0 equiv), gave **4eh** as a white solid (5 mg, 0.012 mmol, 25%); **M.p.**: 120-122 ºC; **IR** (ATR): 1258, 1082, 1017, 792 cm^{-1}; ^{1}**H-NMR** (400 MHz, $CDCl_3$) δ: 7.68-7.55 (3H, m, Ar*H*), 7.16 (1H, d, J = 8.5 Hz, Ar*H*), 7.10 (1H, s, Ar*H*), 6.86 (1H, d, J = 8.5 Hz, Ar*H*), 3.05 (6H, s, N(C*H*$_3$)$_2$), 2.73 (2H, t, J = 7.7 Hz, ArC*H*$_2$), 1.76-1.62 (2H, m, C*H*$_2$), 1.41-1.14 (14H, m, C*H*$_2$), 0.87 (3H, t, J = 6.5 Hz, C*H*$_3$); ^{13}**C-NMR** (126 MHz, $CDCl_3$) δ: 148.7 (ArC), 144.5 (ArC), 141.8 (ArC), 139.1 (ArC), 132.7 (ArC), 131.5 (ArC), 129.4 (ArC), 125.7 (ArCH), 124.0 (ArC), 123.2 (ArCH), 121.7 (ArCH), 120.4 (ArCH), 112.1 (ArCH), 106.1 (ArCH), 41.0 (N(CH$_3$)$_2$), 36.1 (CH$_2$), 31.9 (CH$_2$), 31.8 (CH$_2$), 29.6 (CH$_2$), 29.6 (CH$_2$), 29.5 (CH$_2$), 29.3 (CH$_2$), 29.3 (CH$_2$), 22.7 (CH$_2$), 14.1 (CH$_3$); **HRMS** (APCI): Calcd. for $C_{26}H_{34}NS_2$ $[M+H]^+$: 424.2127; found: 424.2116.

3. ^1H and ^{13}C NMR of BTBT derivatives

^1H NMR of 3ab (400 MHz, $CDCl_3$)

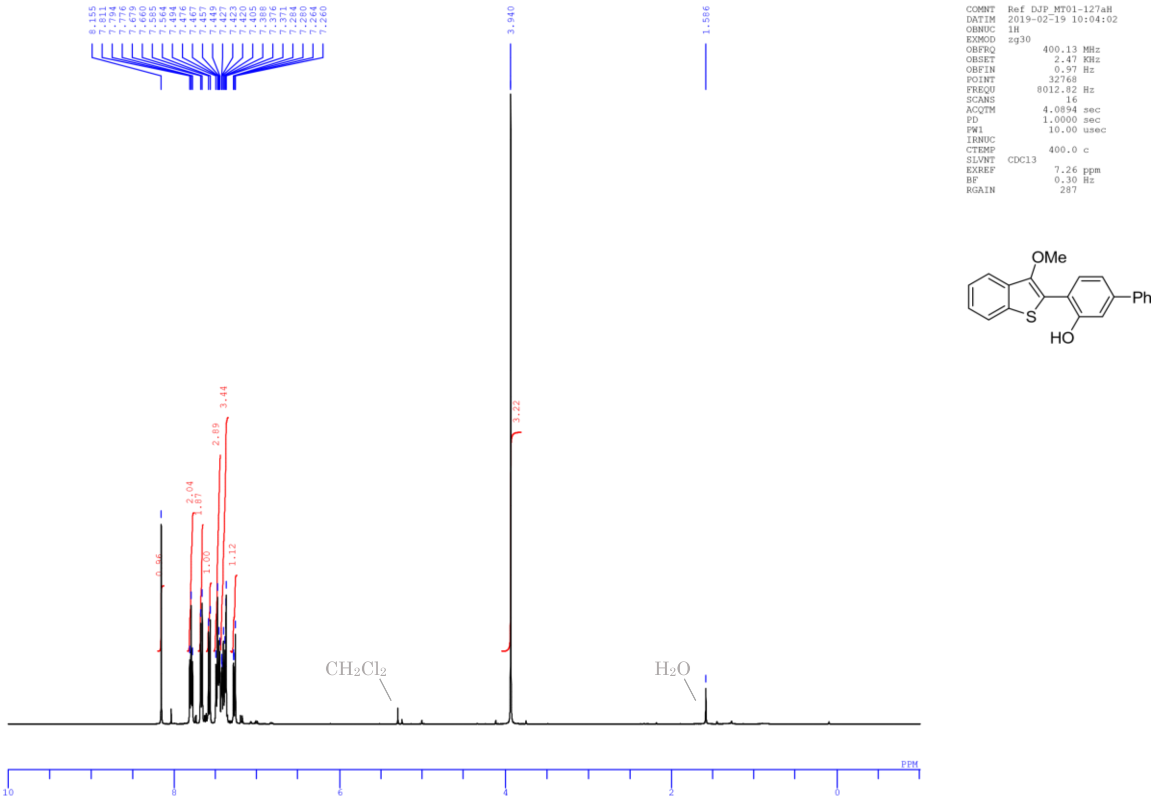

^{13}C NMR of 3ab (126 MHz, $CDCl_3$)

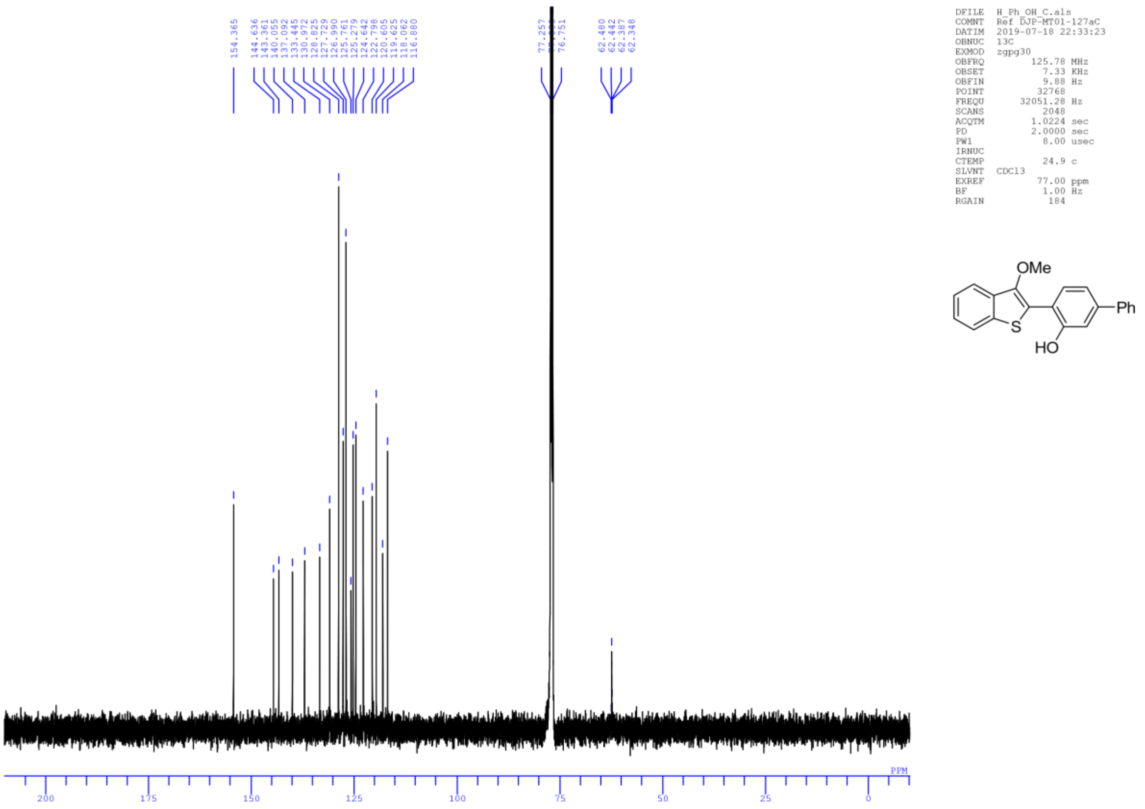

^{1}H NMR of 3ac (500 MHz, $CDCl_3$)

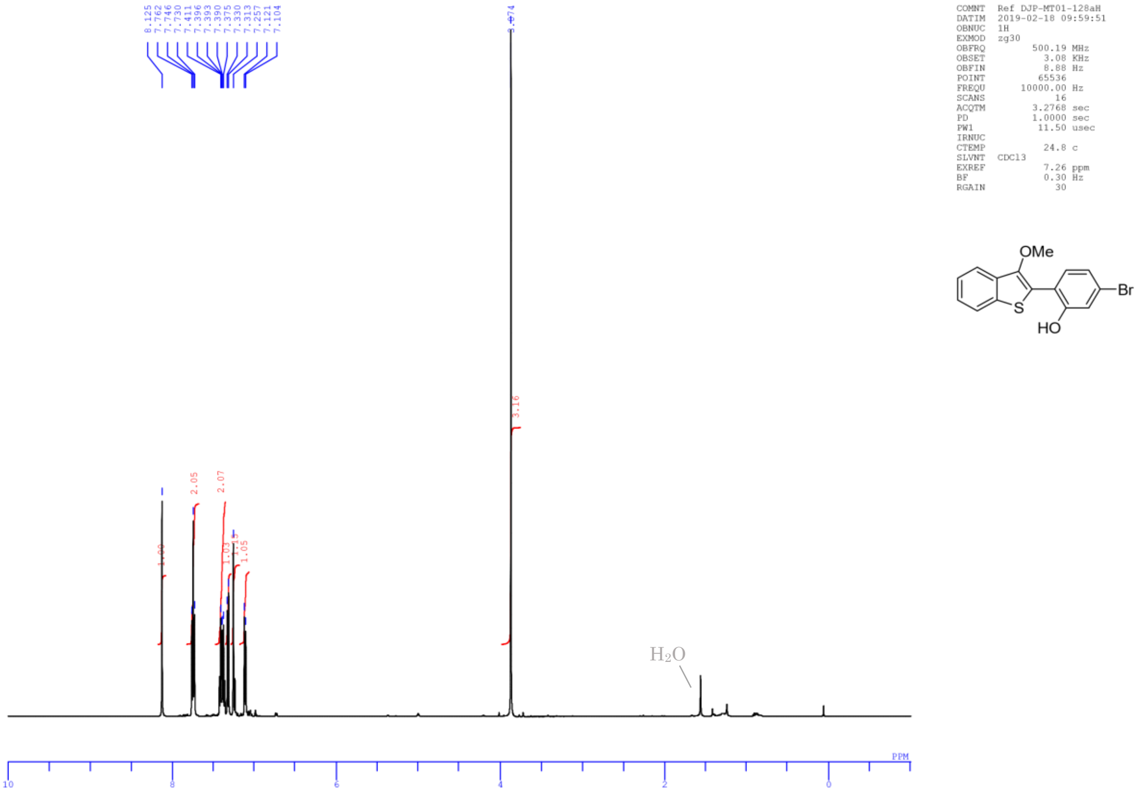

^{13}C NMR of 3ac (126 MHz, $CDCl_3$)

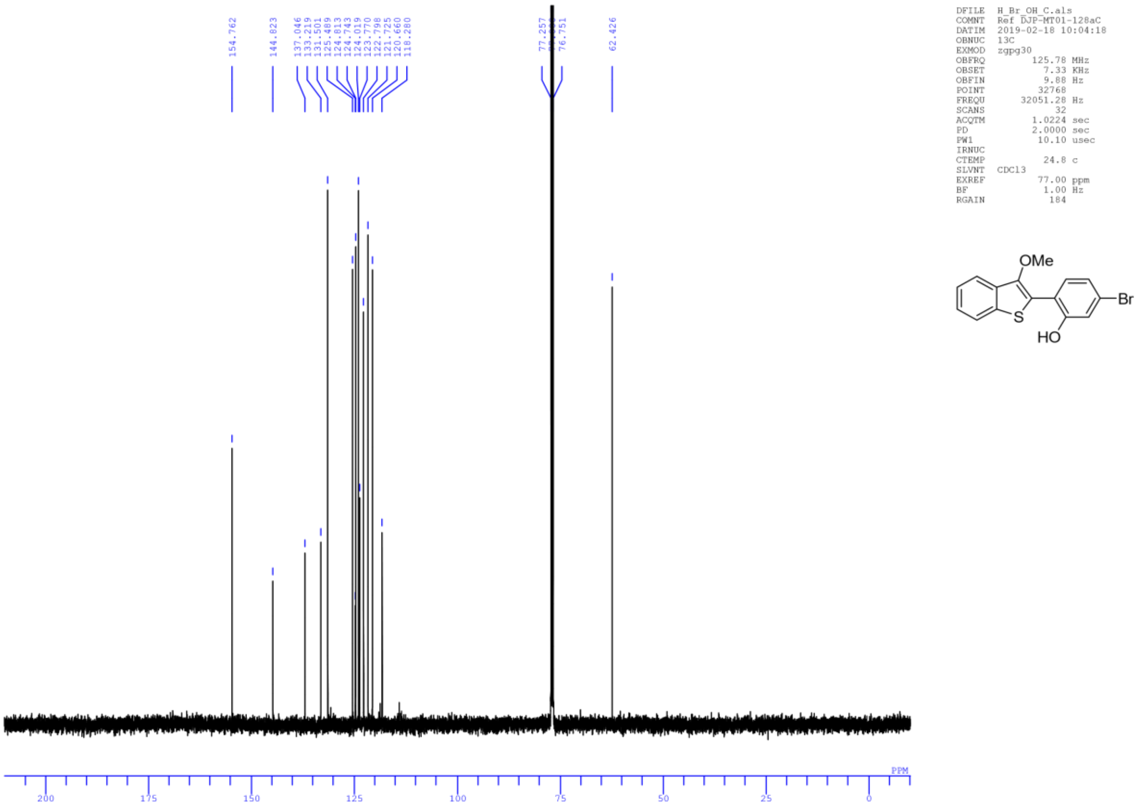

^{1}H NMR of 3ad (500 MHz, $CDCl_3$)

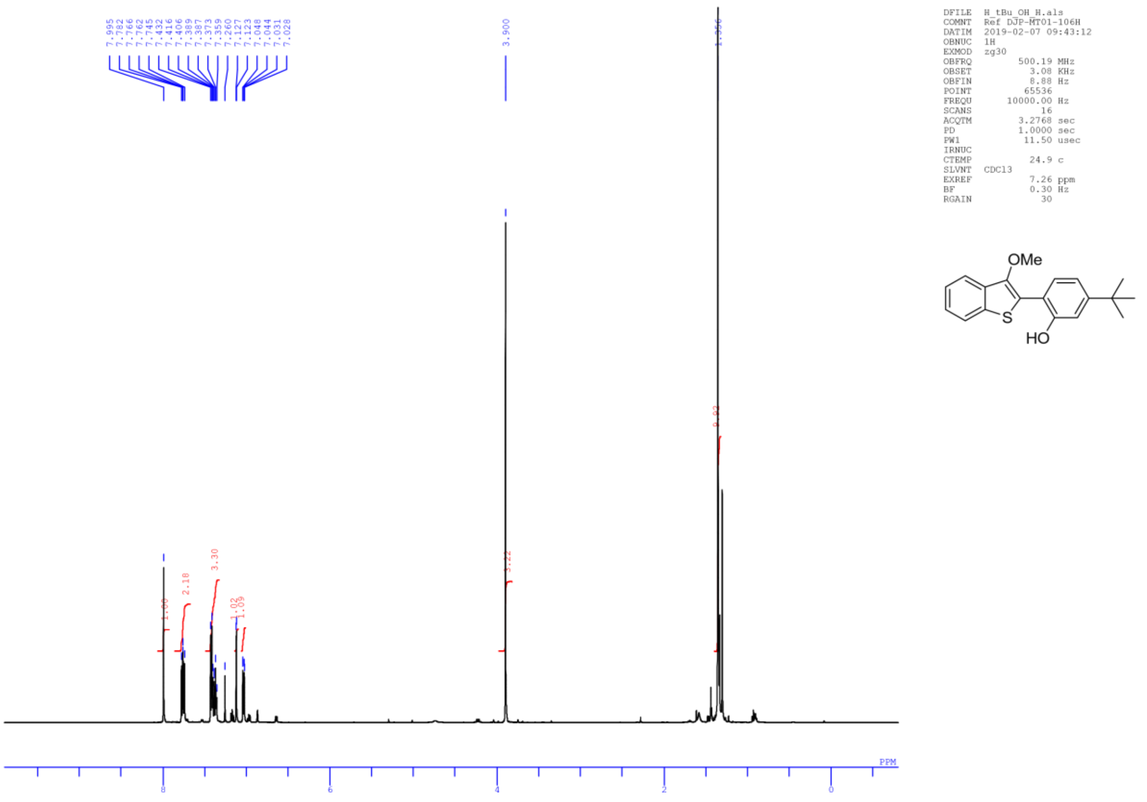

^{13}C NMR of 3ad (126 MHz, $CDCl_3$)

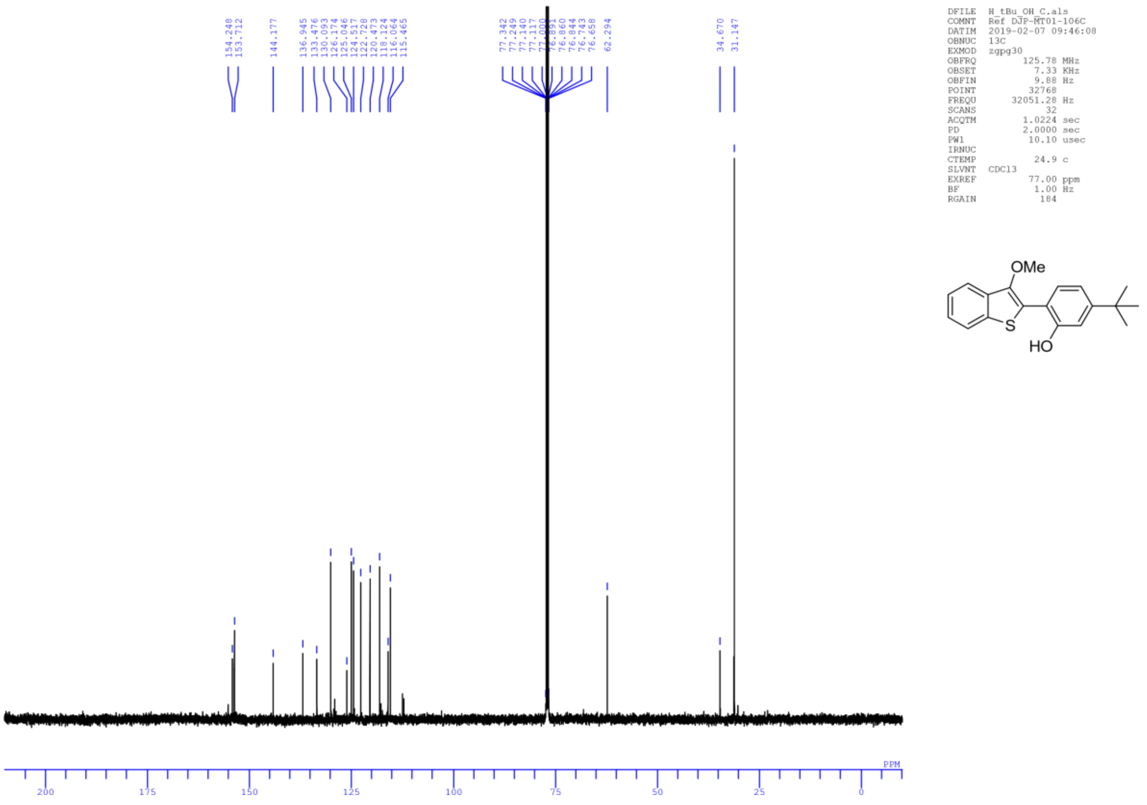

^{1}H NMR of 3ae (400 MHz, $CDCl_3$)

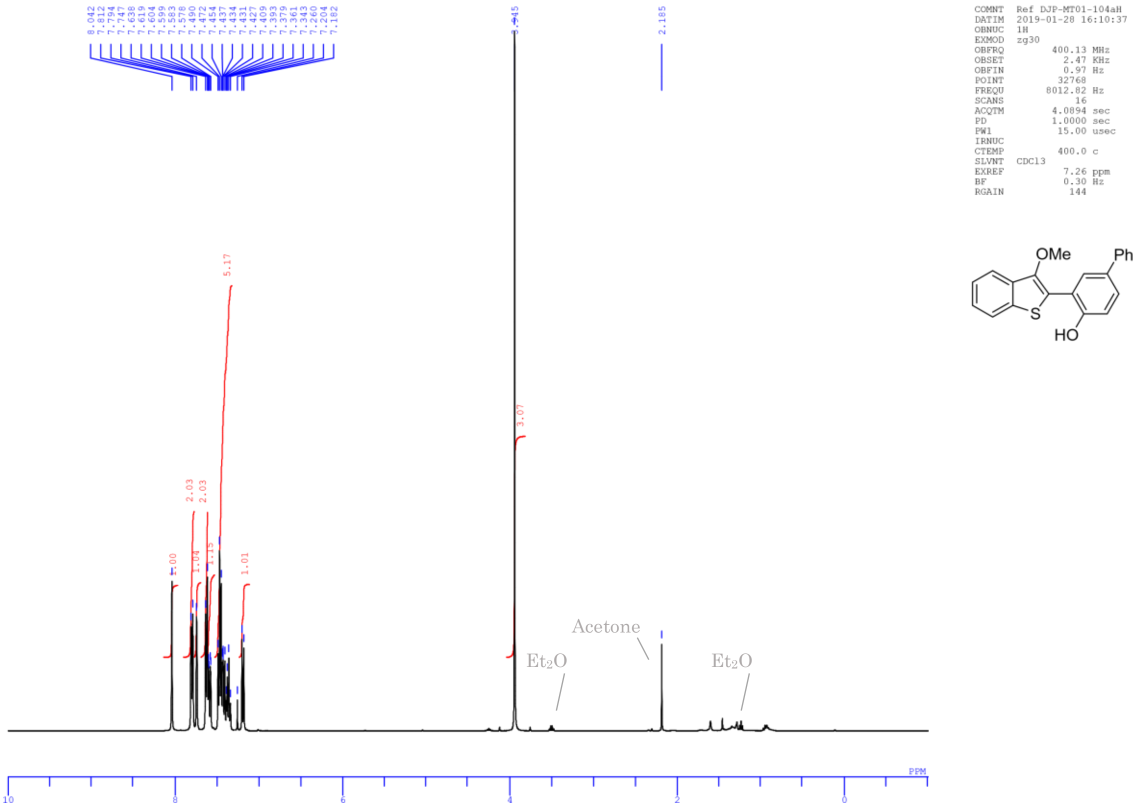

^{13}C NMR of 3ae (126 MHz, $CDCl_3$)

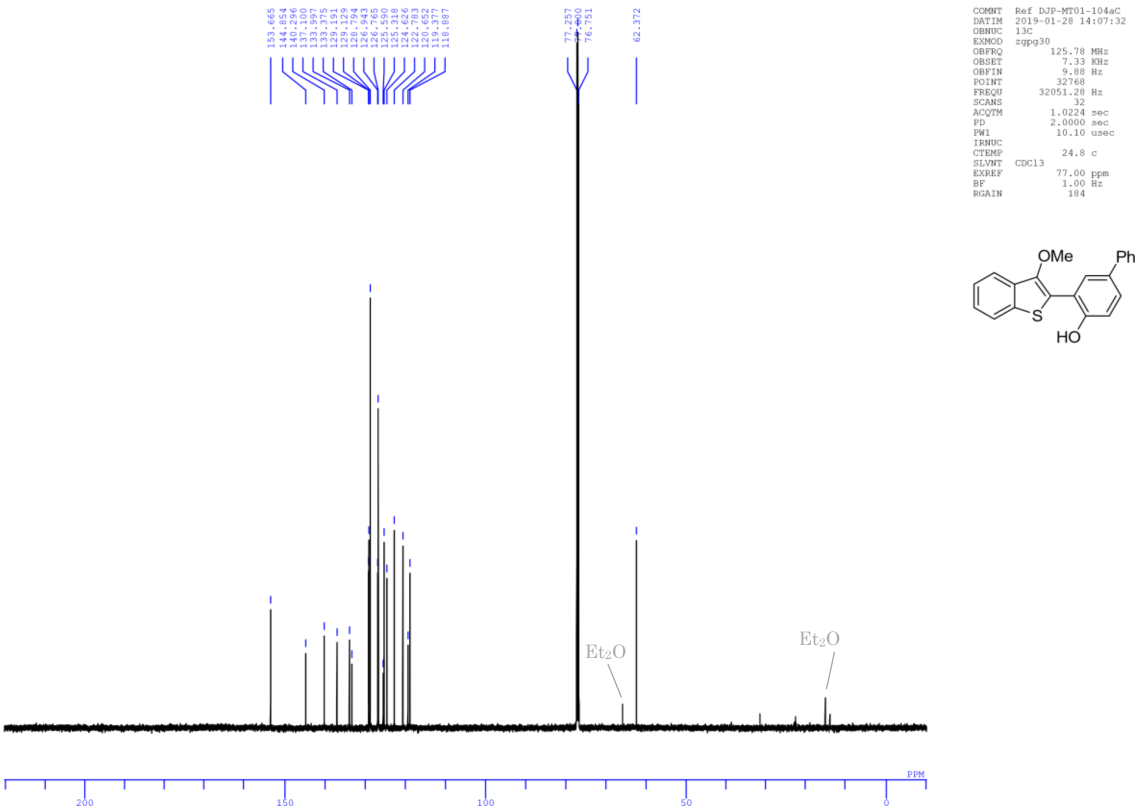

^{1}H NMR of 3bb (400 MHz, $CDCl_3$)

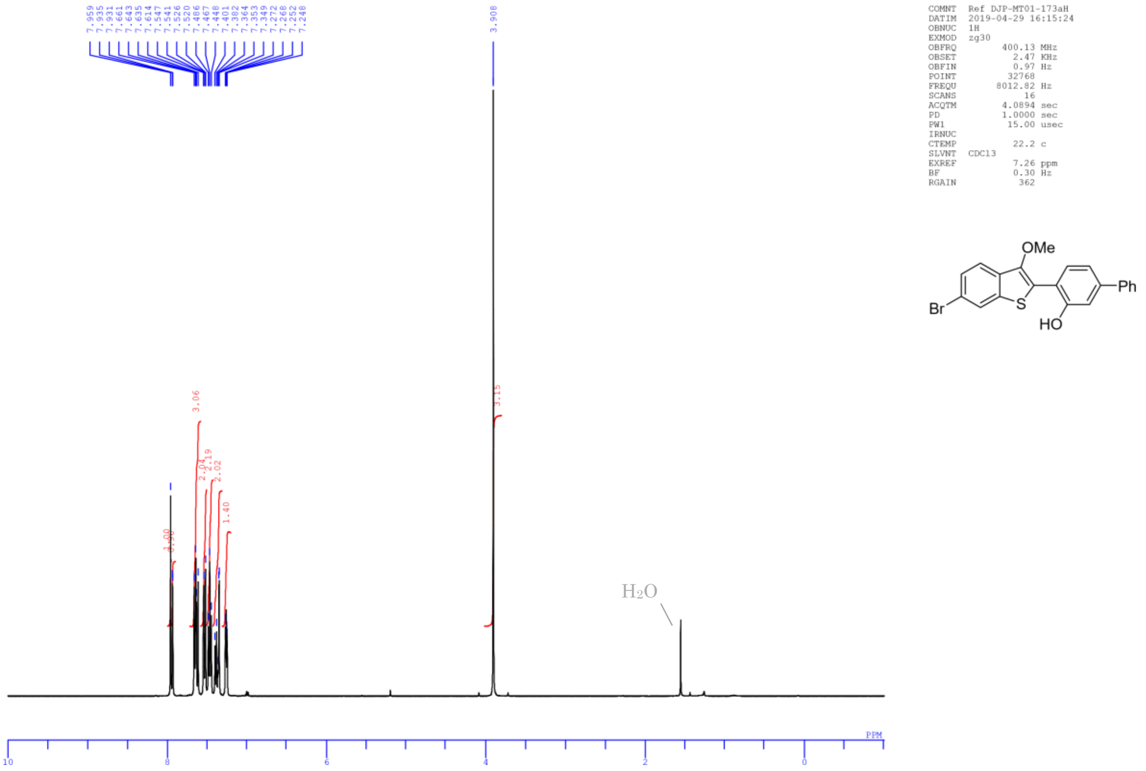

^{13}C NMR of 3bb (126 MHz, $CDCl_3$)

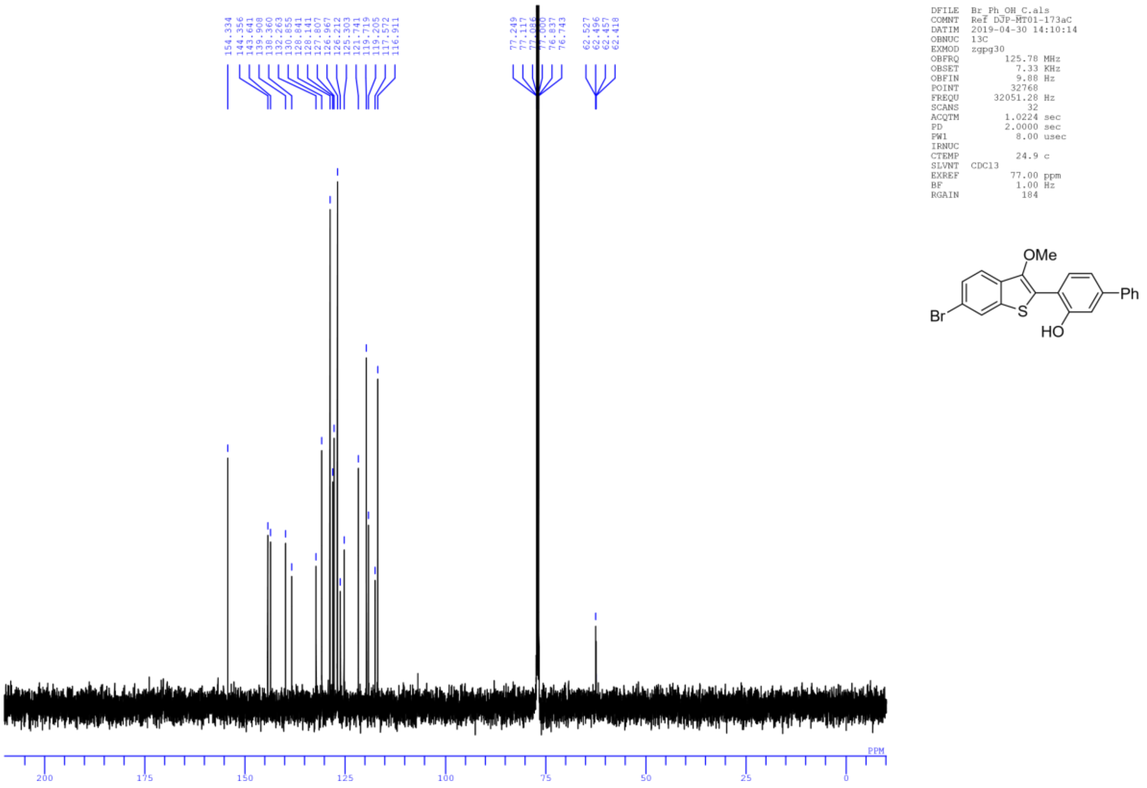

^{1}H NMR of 3bd (500 MHz, $CDCl_3$)

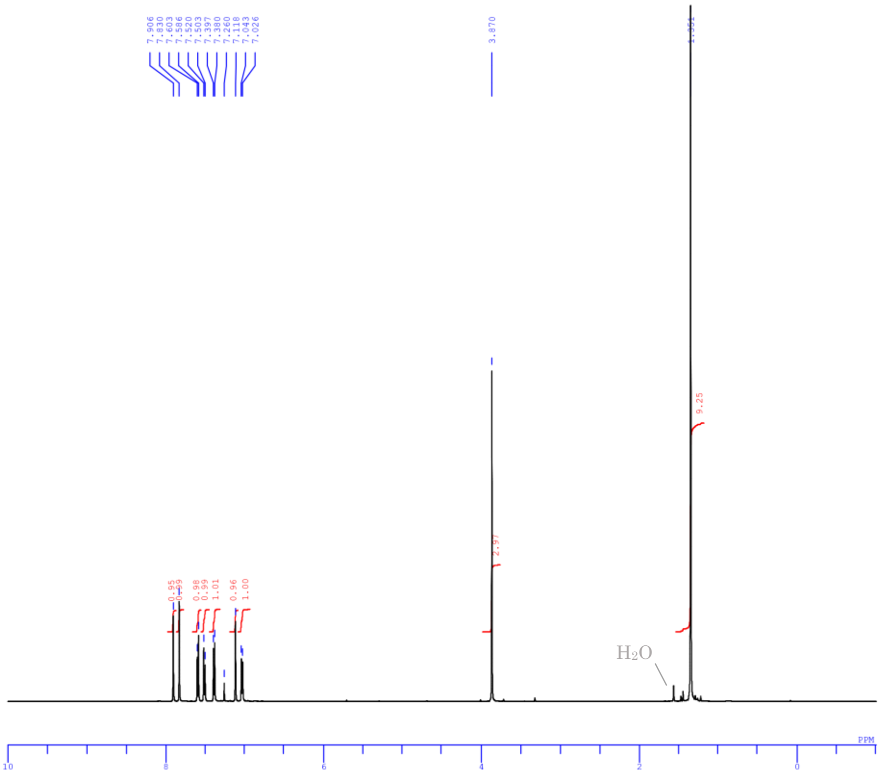

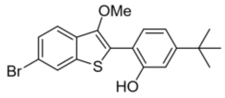

^{13}C NMR of 3bd (126 MHz, $CDCl_3$)

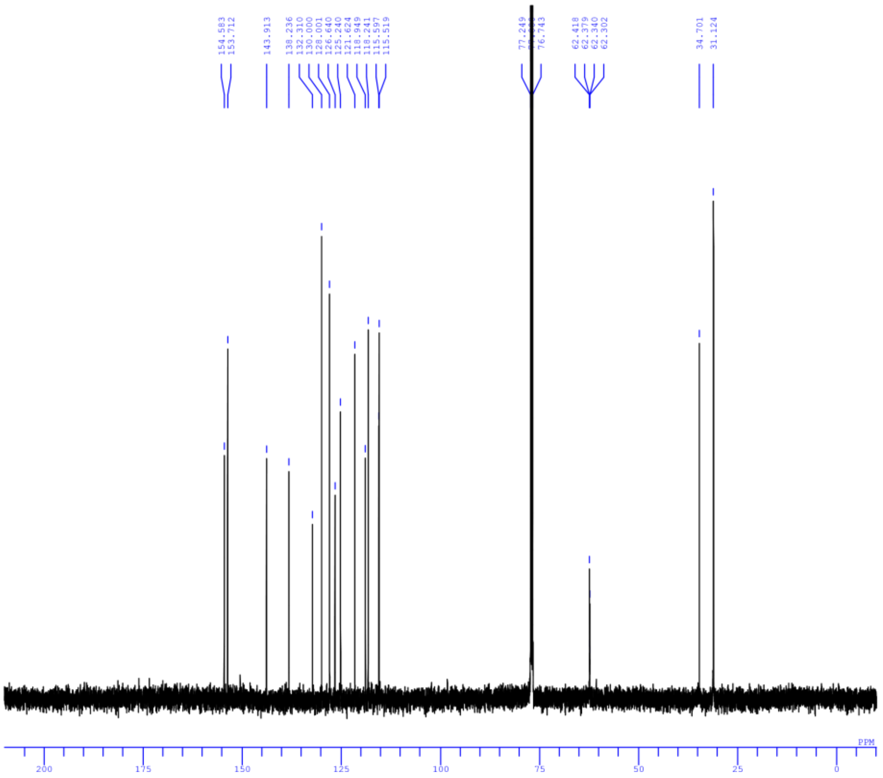

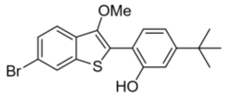

^{1}H NMR of 3bf (400 MHz, $CDCl_3$)

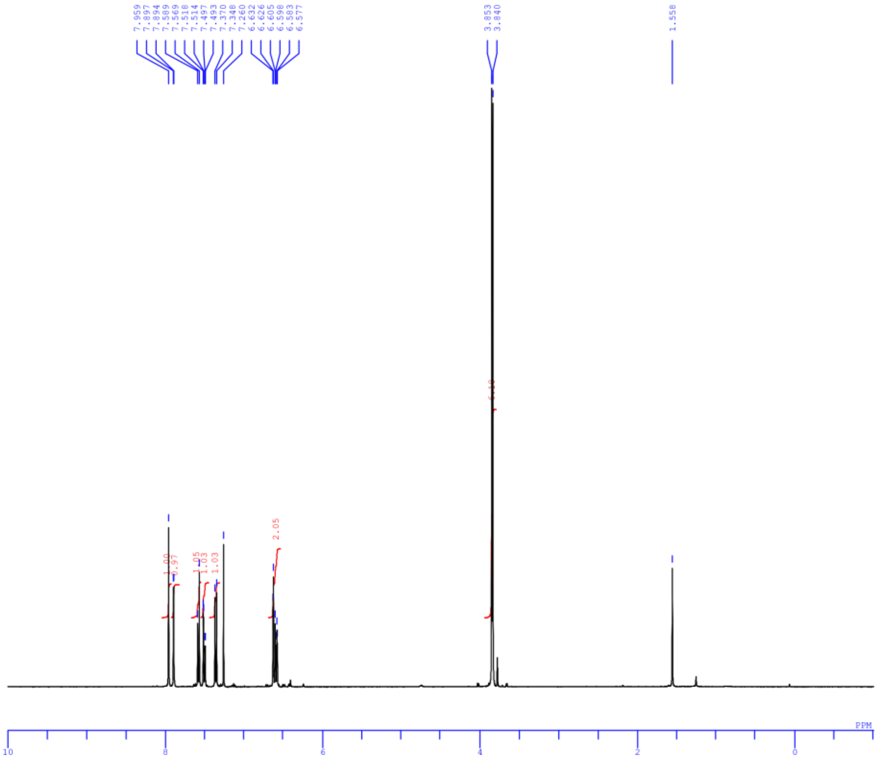

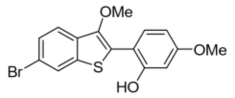

^{13}C NMR of 3bf (101 MHz, $CDCl_3$)

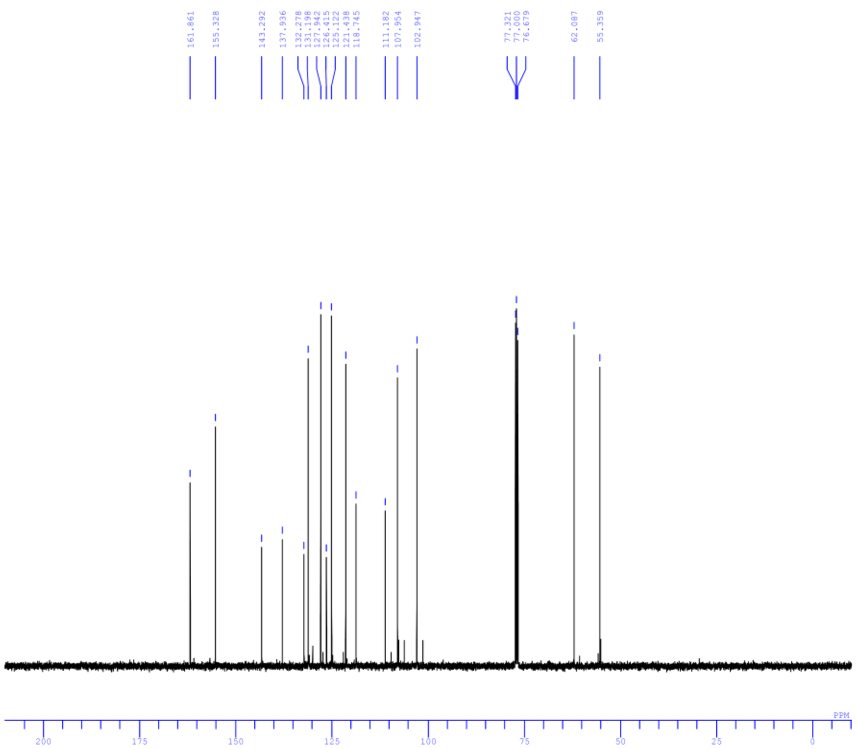

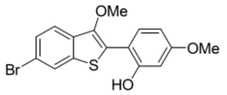

^{1}H NMR of 3bg (400 MHz, CDCl$_3$)

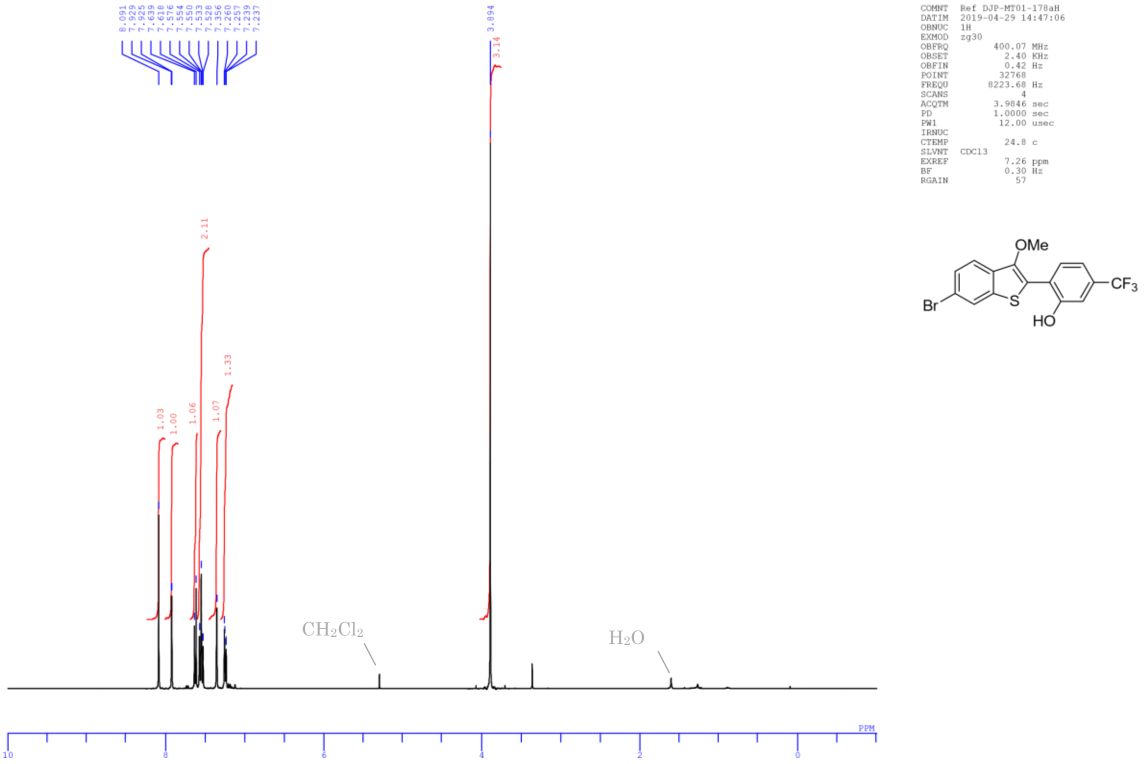

^{13}C NMR of 3bg (101 MHz, CDCl$_3$)

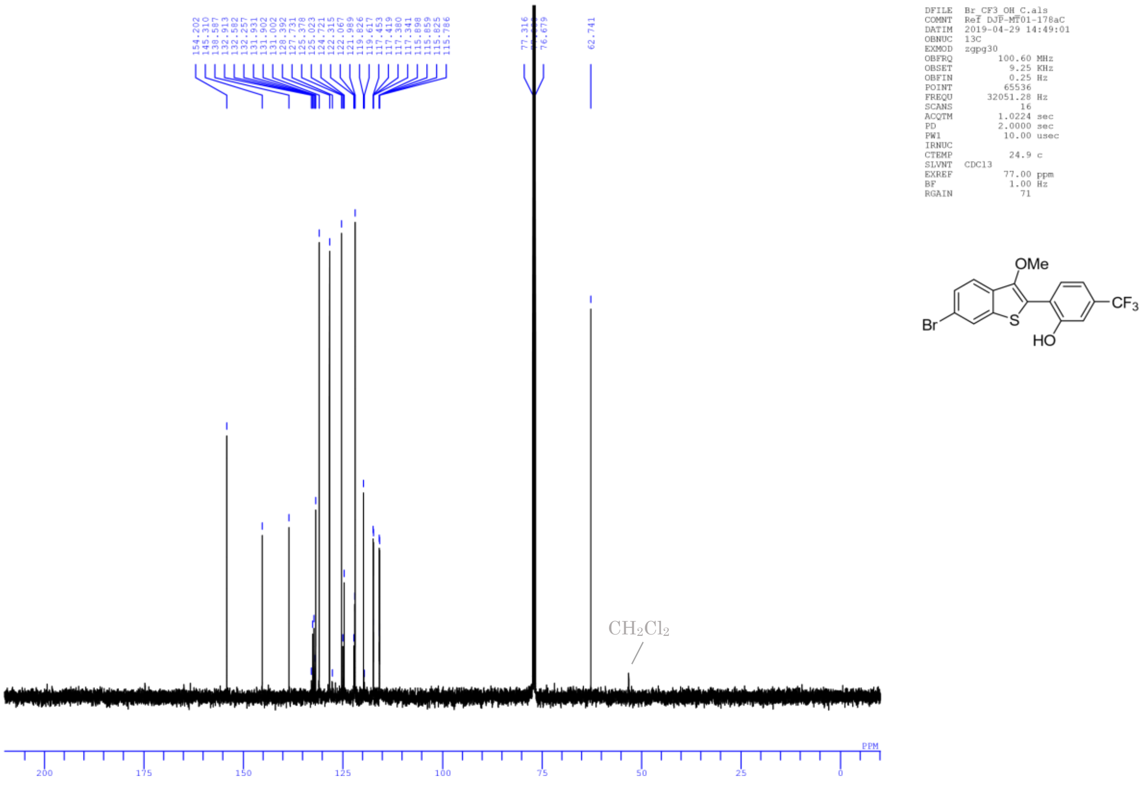

^{1}H NMR of 3cd (400 MHz, $CDCl_3$)

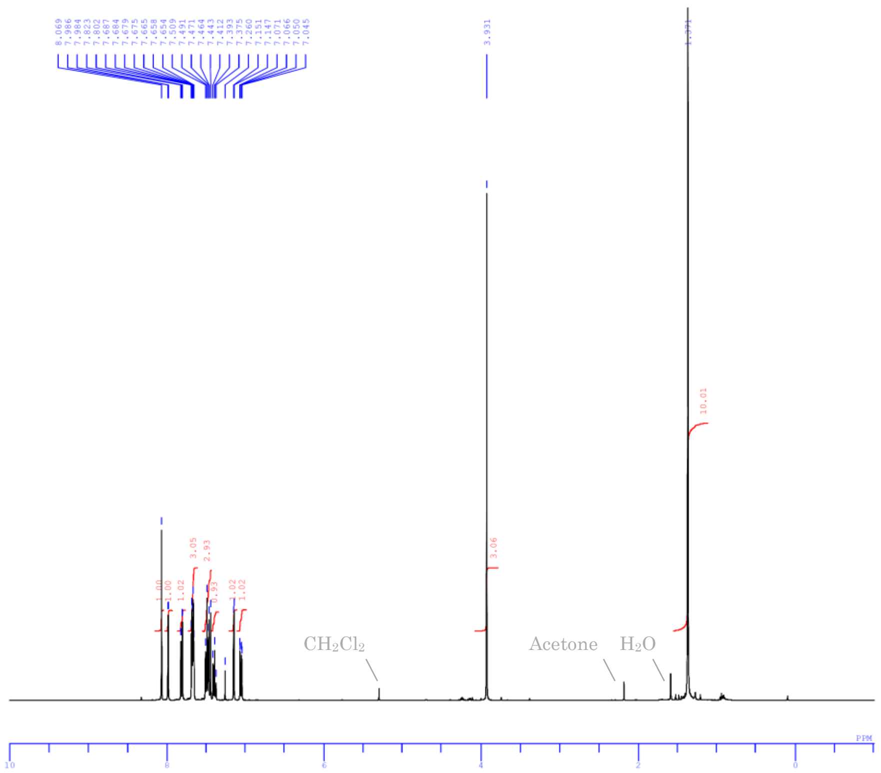

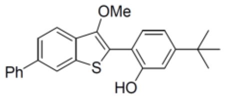

^{13}C NMR of 3cd (101 MHz, $CDCl_3$)

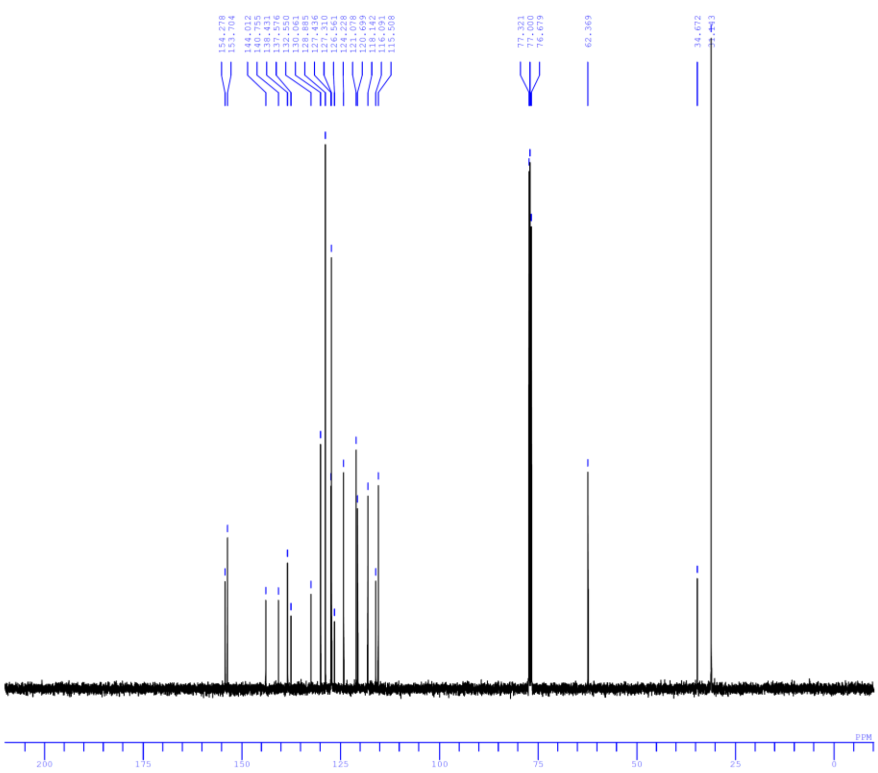

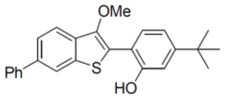

^{1}H NMR of 3ce (400 MHz, $CDCl_3$)

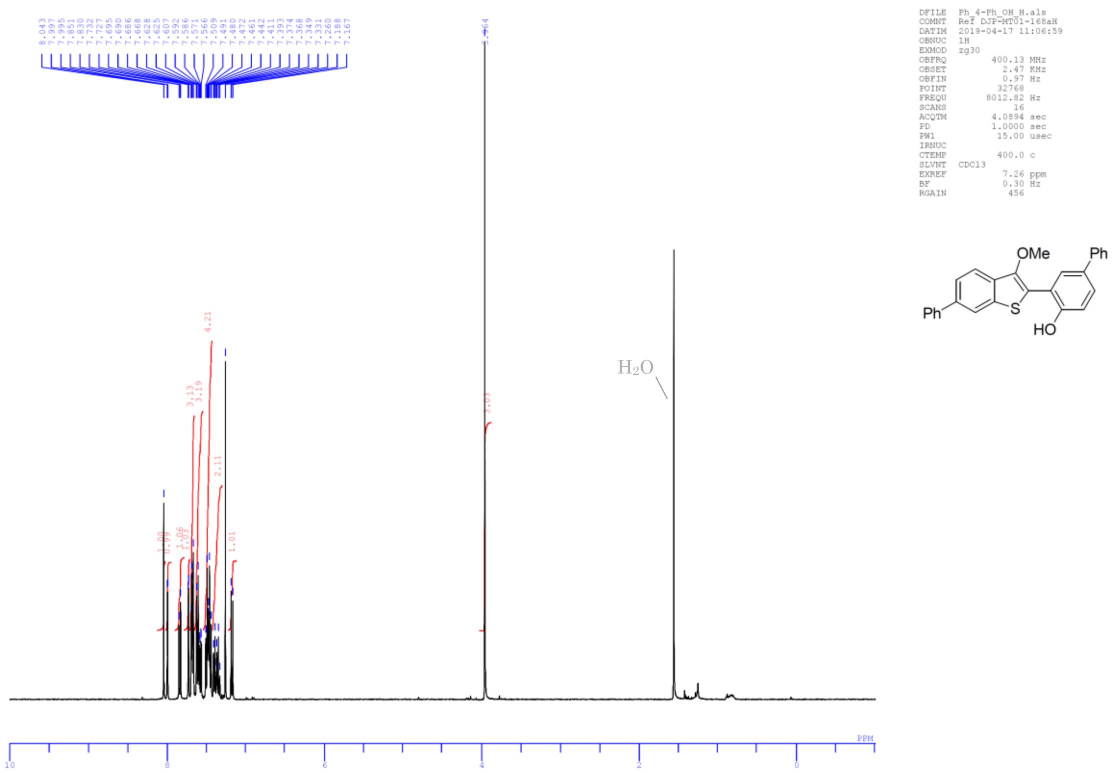

^{13}C NMR of 3ce (101 MHz, $CDCl_3$)

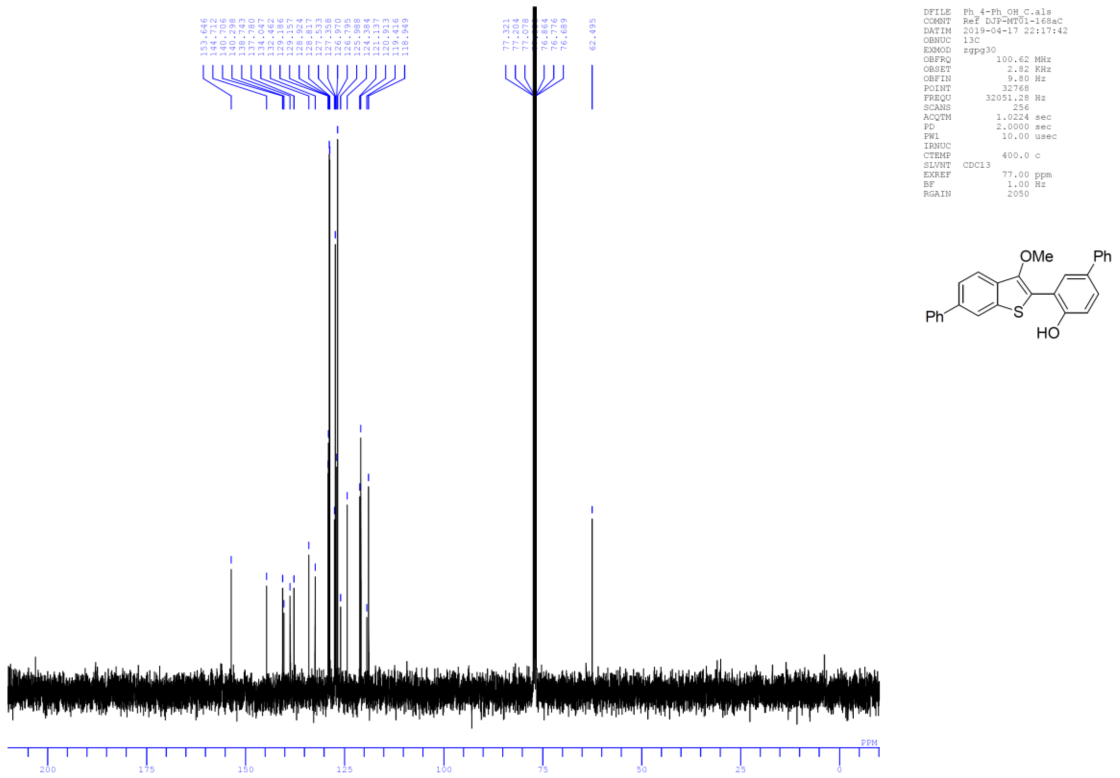

^{1}H NMR of 3ef (500 MHz, CDCl$_3$)

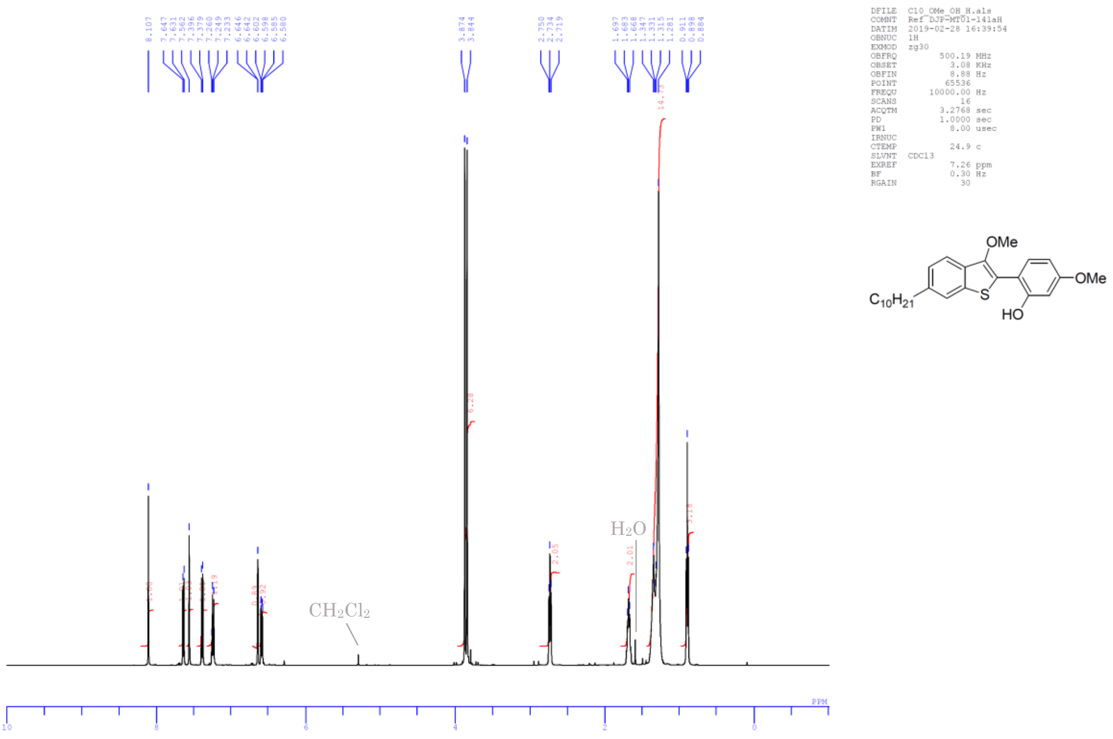

^{13}C NMR of 3ef (126 MHz, CDCl$_3$)

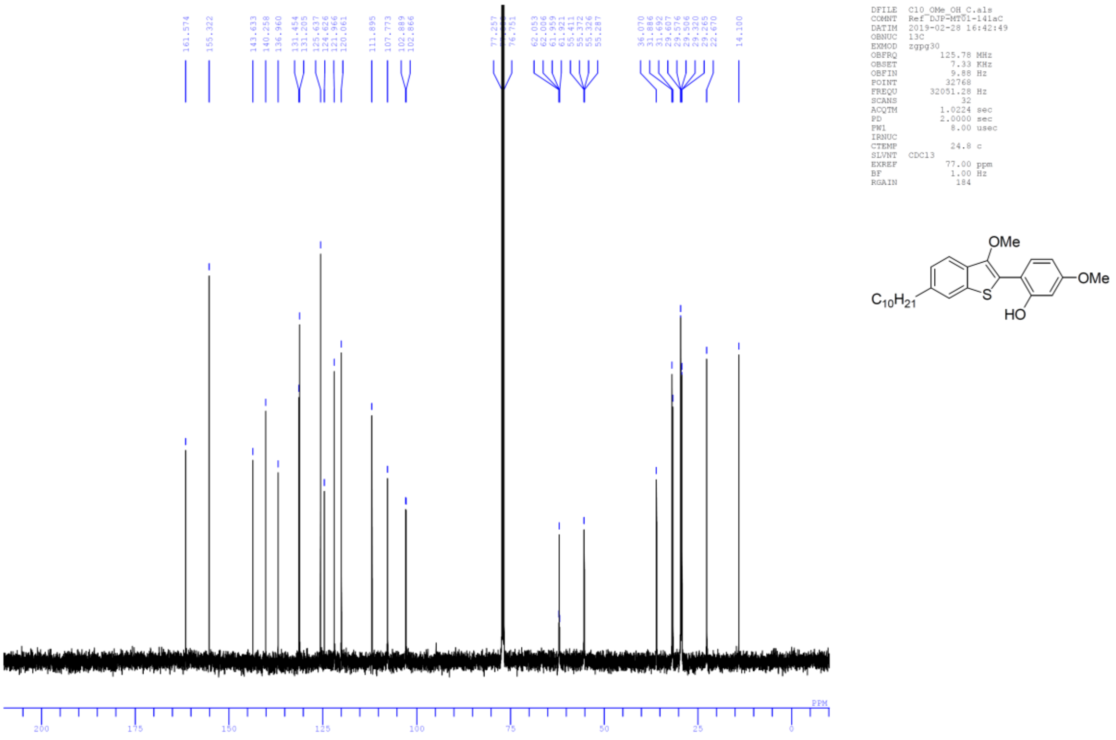

^{1}H NMR of 3eg (500 MHz, $CDCl_3$)

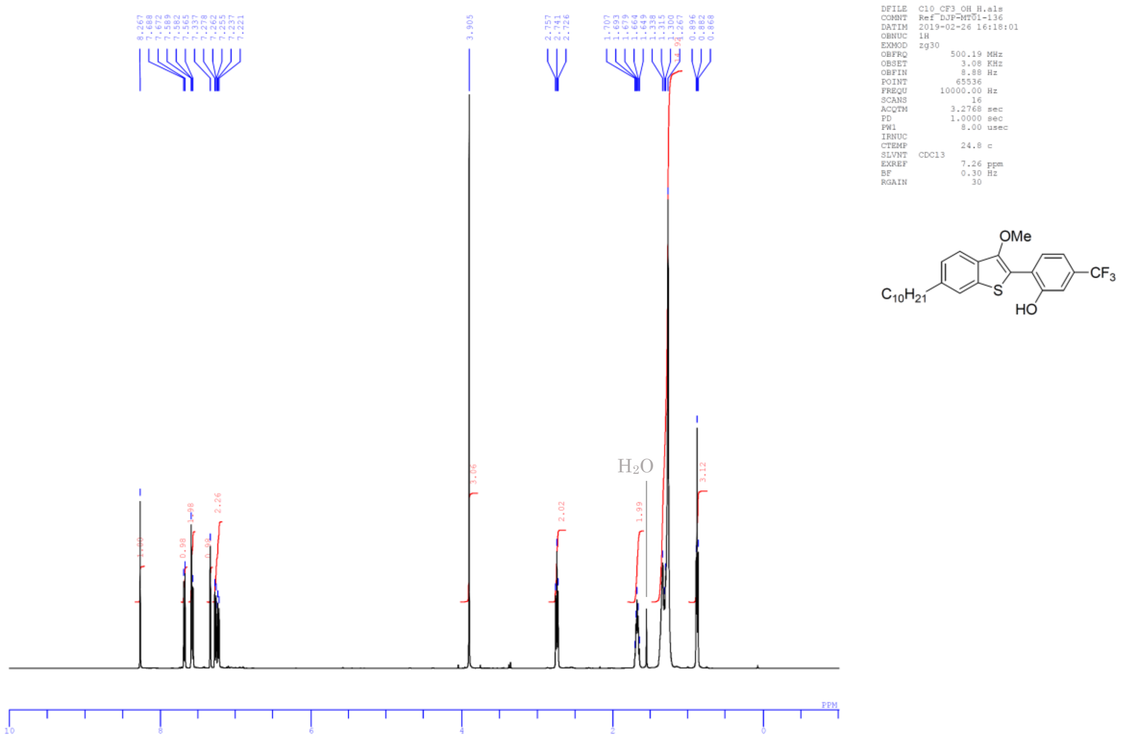

^{13}C NMR of 3eg (126 MHz, $CDCl_3$)

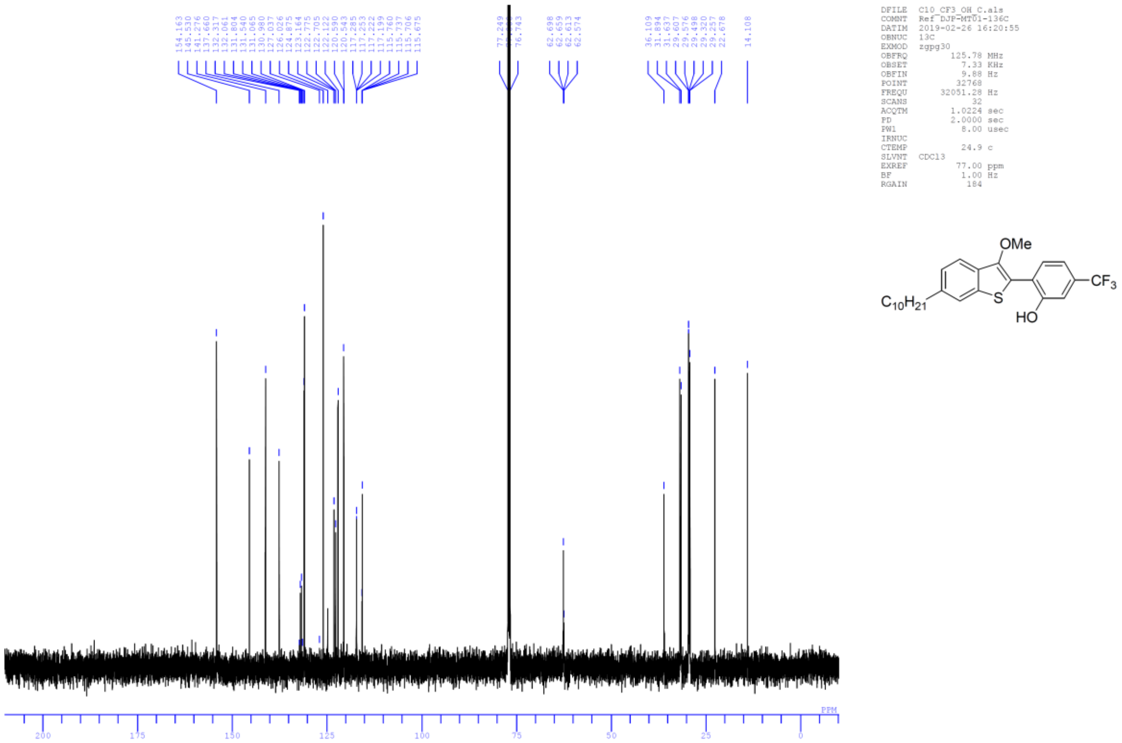

^{1}H NMR of 3eh (400 MHz, $CDCl_3$)

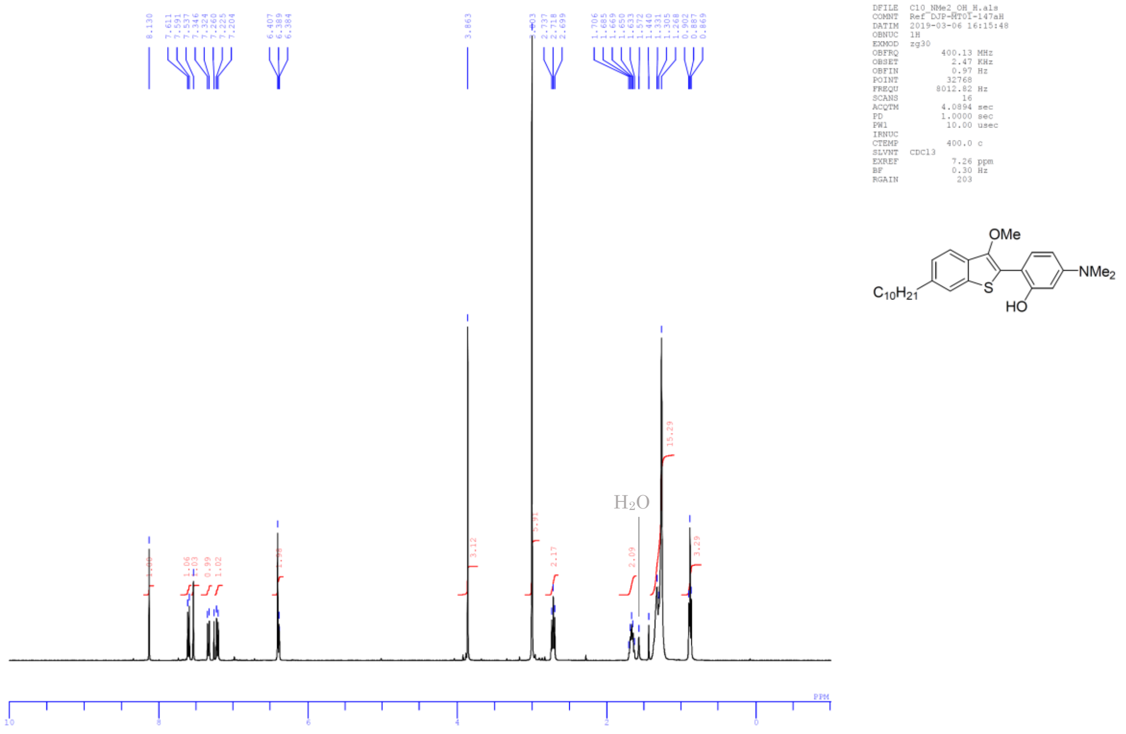

^{13}C NMR of 3eh (126 MHz, $CDCl_3$)

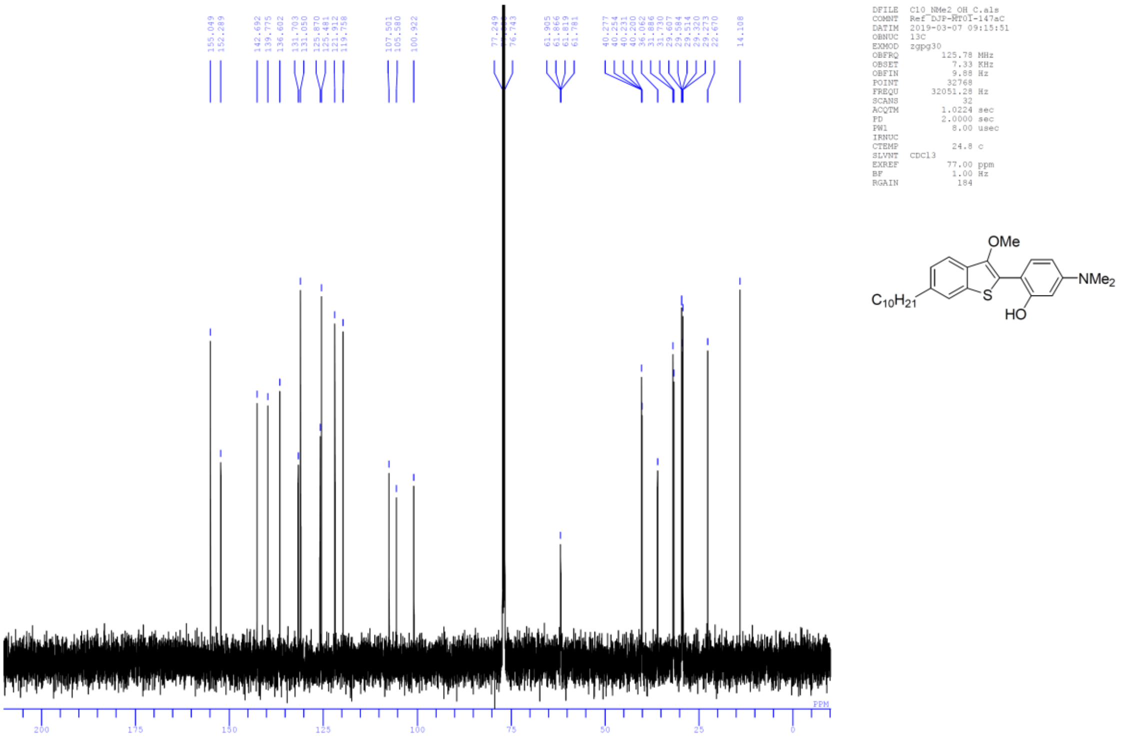

^{1}H NMR of 5aa (500 MHz, $CDCl_3$)

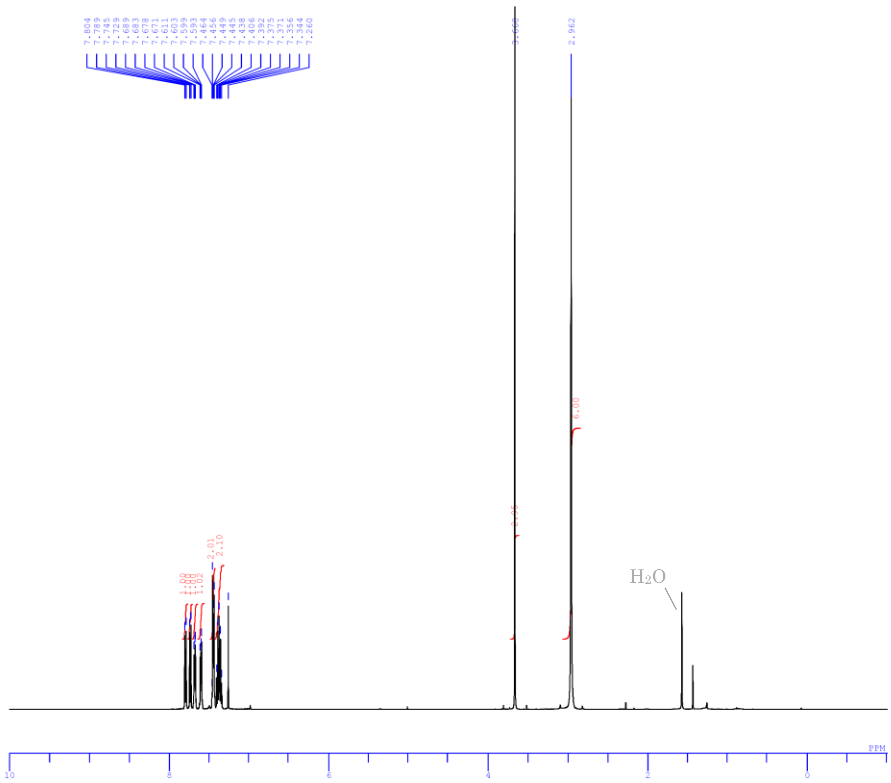


```
DFILE  H_H_S_H.als
COMNT  Ref DJP-MT HHS H
DATIM  2019-07-18 11:07:44
OBNUC  1H
EXMOD  zg30
OBFRQ        500.19 MHz
OBSET          3.08 KHz
OBFIN          8.88 Hz
POINT         65536
FREQU      10000.00 Hz
SCANS            16
ACQTM        3.2768 sec
PD           1.0000 sec
PW1            8.00 usec
IRNUC
CTEMP          24.9 c
SLVNT  CDCl3
EXREF          7.26 ppm
BF             0.30 Hz
RGAIN            68
```

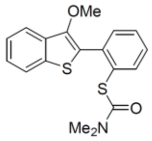

^{13}C NMR of 5aa (126MHz, $CDCl_3$)

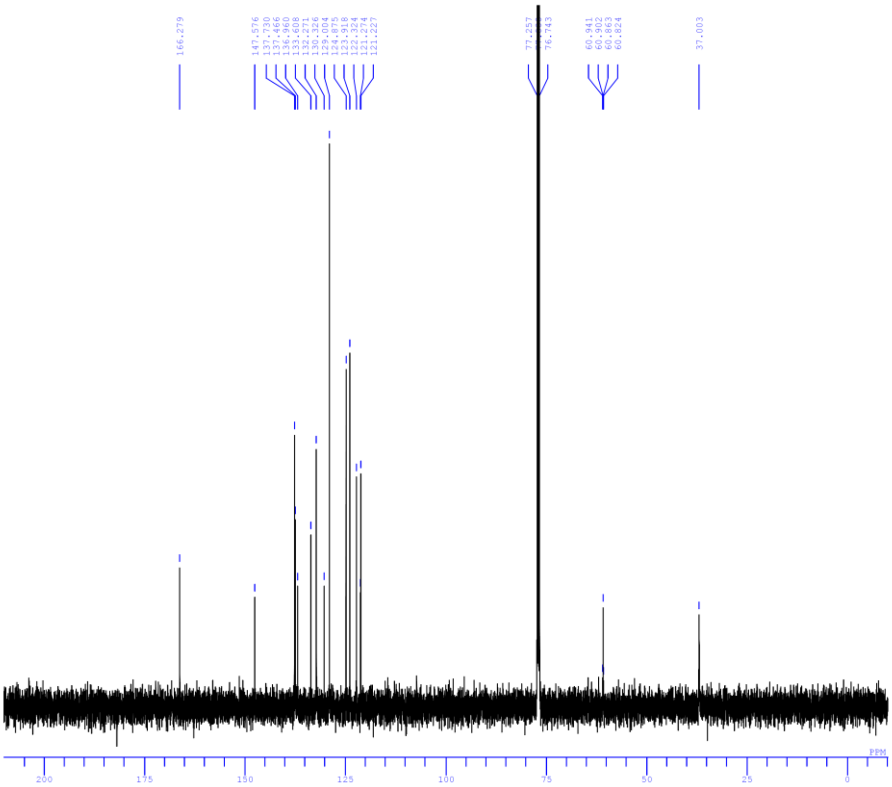


```
DFILE  H_H_S_C.als
COMNT  Ref DJP-MT HHS C
DATIM  2019-07-18 10:35:24
OBNUC  13C
EXMOD  zgpg30
OBFRQ        125.78 MHz
OBSET          7.33 KHz
OBFIN          9.88 Hz
POINT         32768
FREQU      32051.28 Hz
SCANS            32
ACQTM        1.0224 sec
PD           2.0000 sec
PW1            8.00 usec
IRNUC
CTEMP          24.8 c
SLVNT  CDCl3
EXREF         77.00 ppm
BF             1.00 Hz
RGAIN           184
```

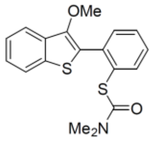

^{1}H NMR of 5ab (500 MHz, $CDCl_3$)

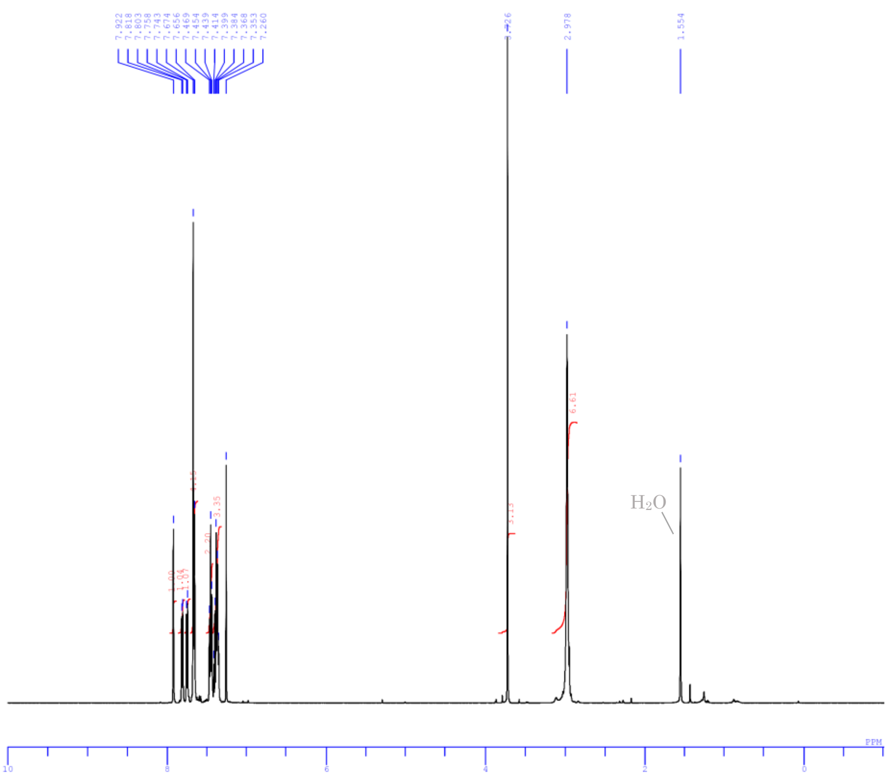

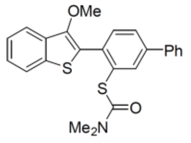

^{13}C NMR of 5ab (126 MHz, $CDCl_3$)

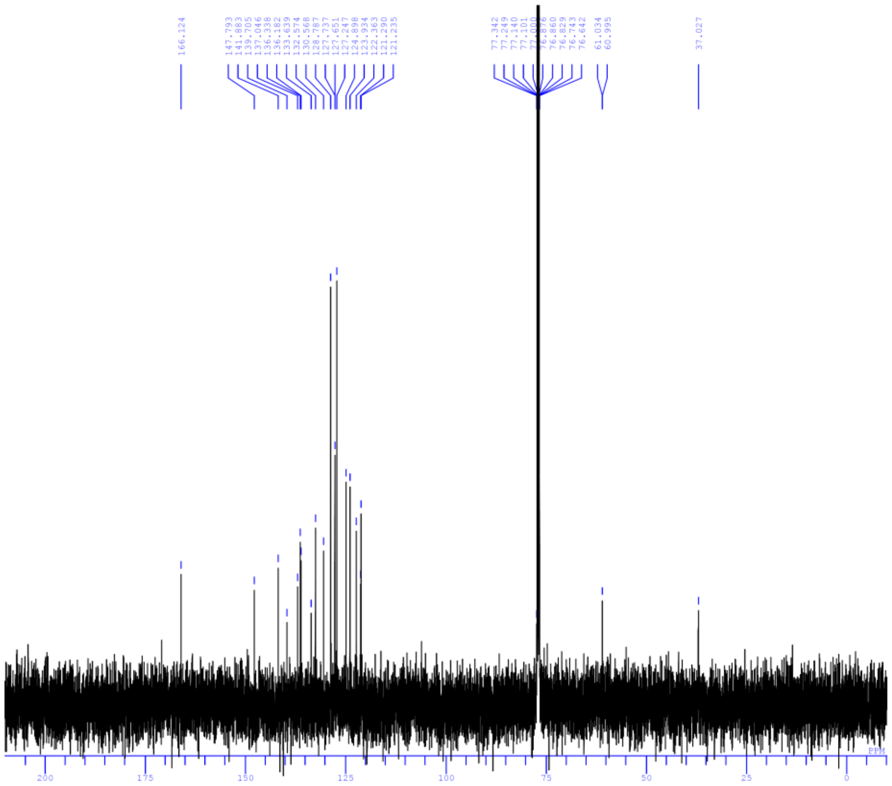

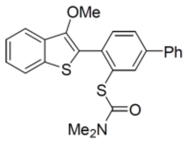

^{1}H NMR of 5ac (400 MHz, $CDCl_3$)

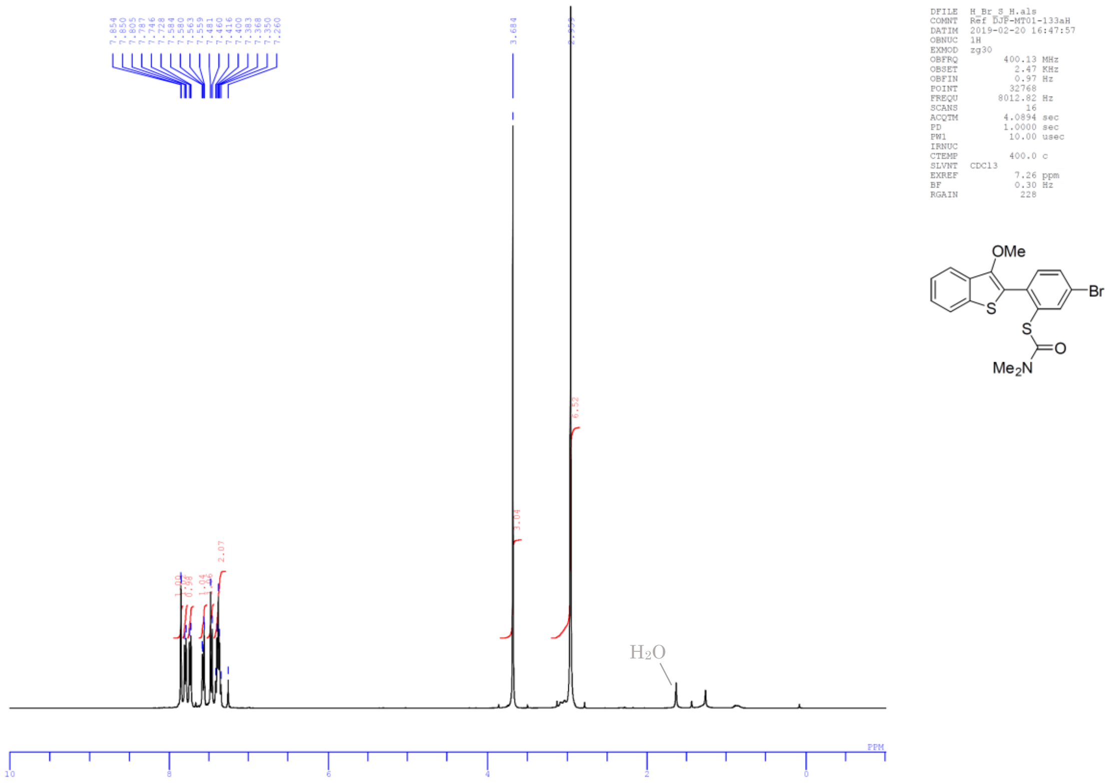

^{13}C NMR of 5ac (101 MHz, $CDCl_3$)

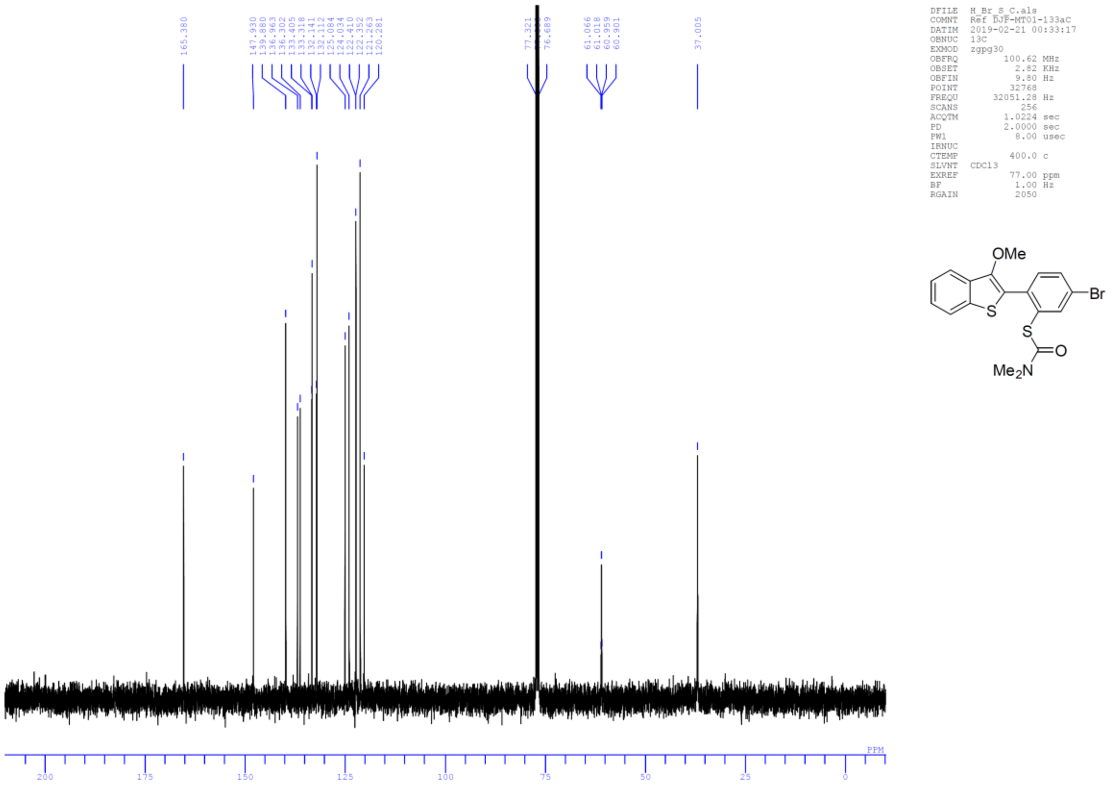

^{1}H NMR of 5ad (500 MHz, $CDCl_3$)

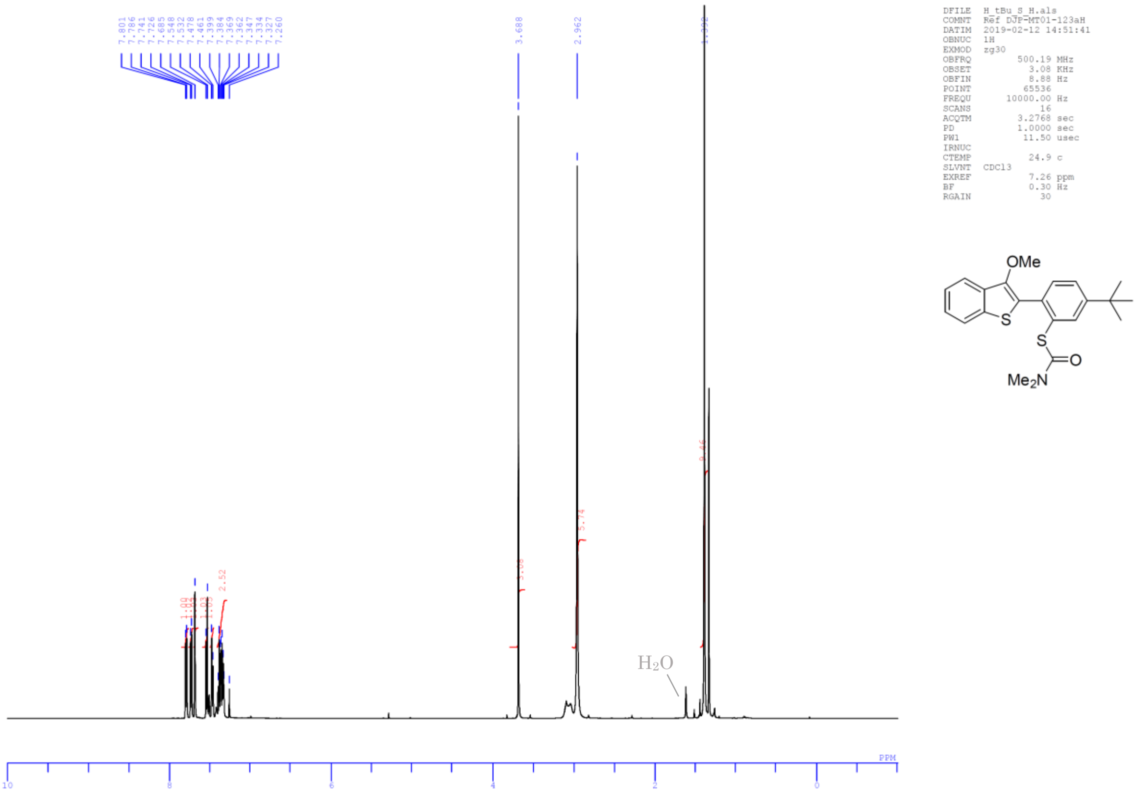

^{13}C NMR of 5ad (126 MHz, $CDCl_3$)

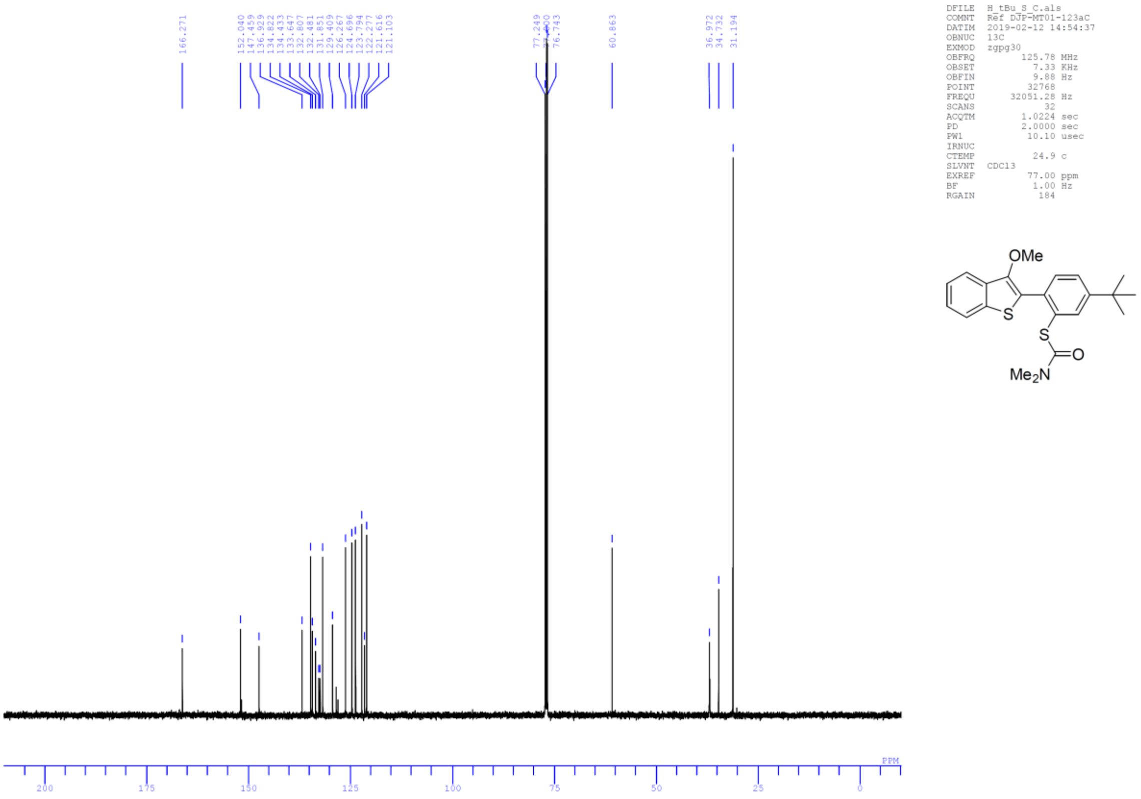

^{1}H NMR of 5ae (500 MHz, $CDCl_3$)

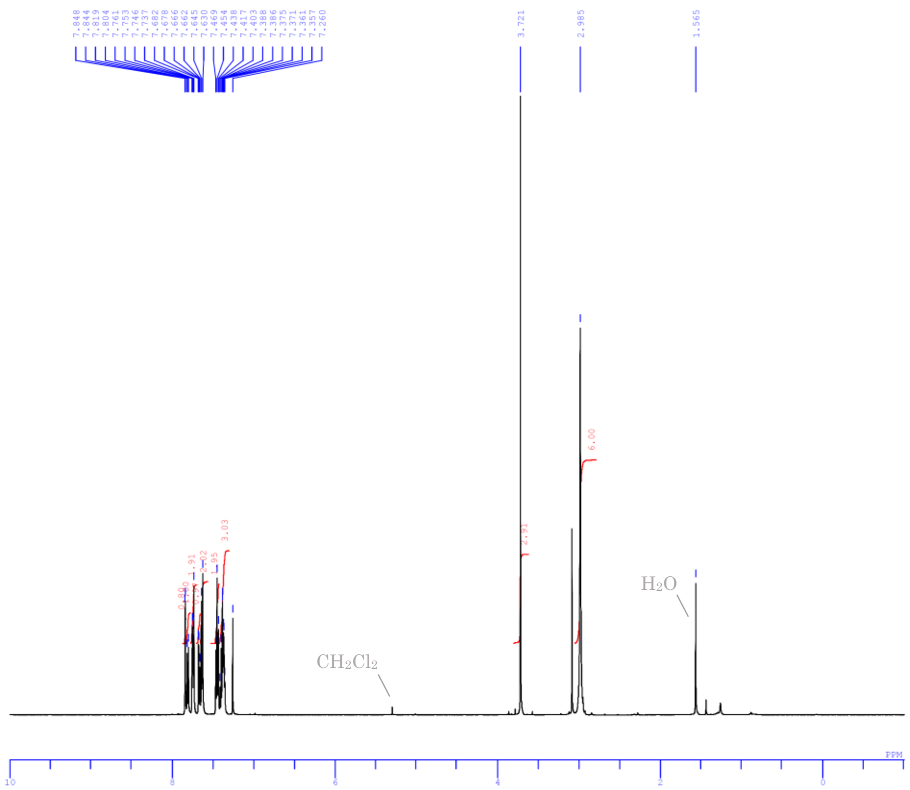

DFILE H_4-Ph_S_H.als
COMNT Ref DJF-MT01-108H
DATIM 2019-02-06 09:27:20
OBNUC 1H
EXMOD zg30
OBFRQ 500.19 MHz
OBSET 3.08 KHz
OBFIN 8.88 Hz
POINT 65536
FREQU 10000.00 Hz
SCANS 16
ACQTM 3.2768 sec
PD 1.0000 sec
PW1 11.50 usec
IRNUC
CTEMP 24.8 c
SLVNT CDCL3
EXREF 1.57 ppm
BF 0.30 Hz
RGAIN 47

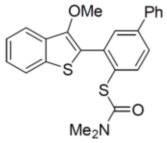

^{13}C NMR of 5ae (126 MHz, $CDCl_3$)

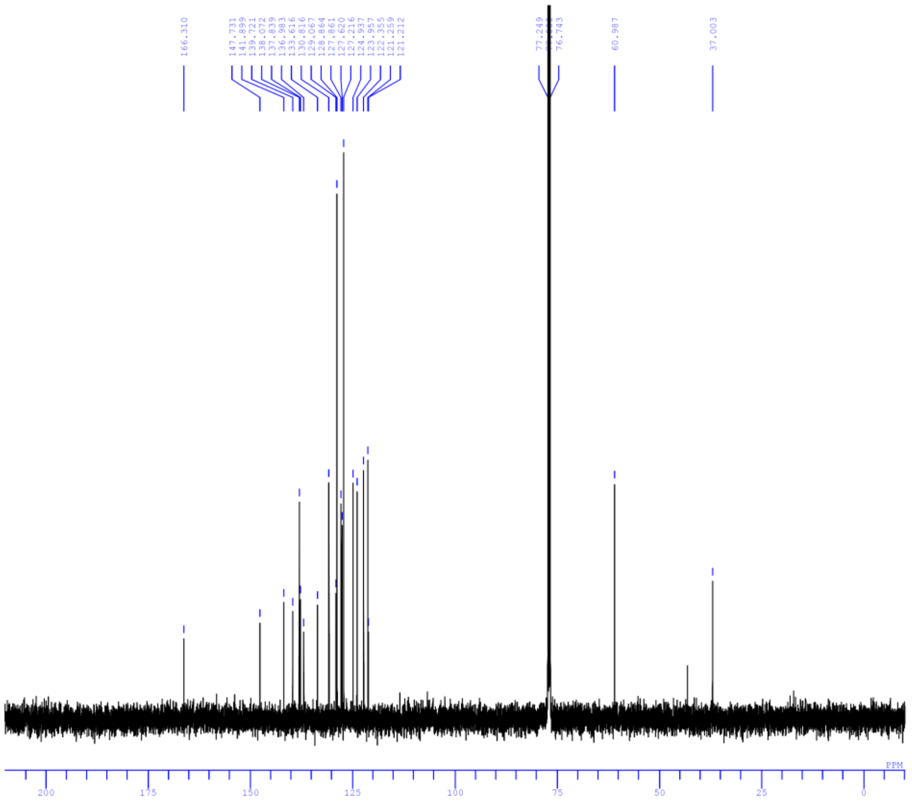

DFILE H_4-Ph_S_C.als
COMNT Ref DJF-MT01-108C
DATIM 2019-02-06 09:30:14
OBNUC 13C
EXMOD zgpg30
OBFRQ 125.78 MHz
OBSET 7.33 KHz
OBFIN 9.88 Hz
POINT 32768
FREQU 32051.28 Hz
SCANS 32
ACQTM 1.0224 sec
PD 2.0000 sec
PW1 10.10 usec
IRNUC
CTEMP 24.8 c
SLVNT CDCL3
EXREF 77.00 ppm
BF 1.00 Hz
RGAIN 184

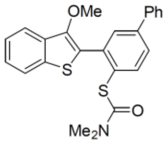

^{1}H NMR of 5bb (400 MHz, $CDCl_3$)

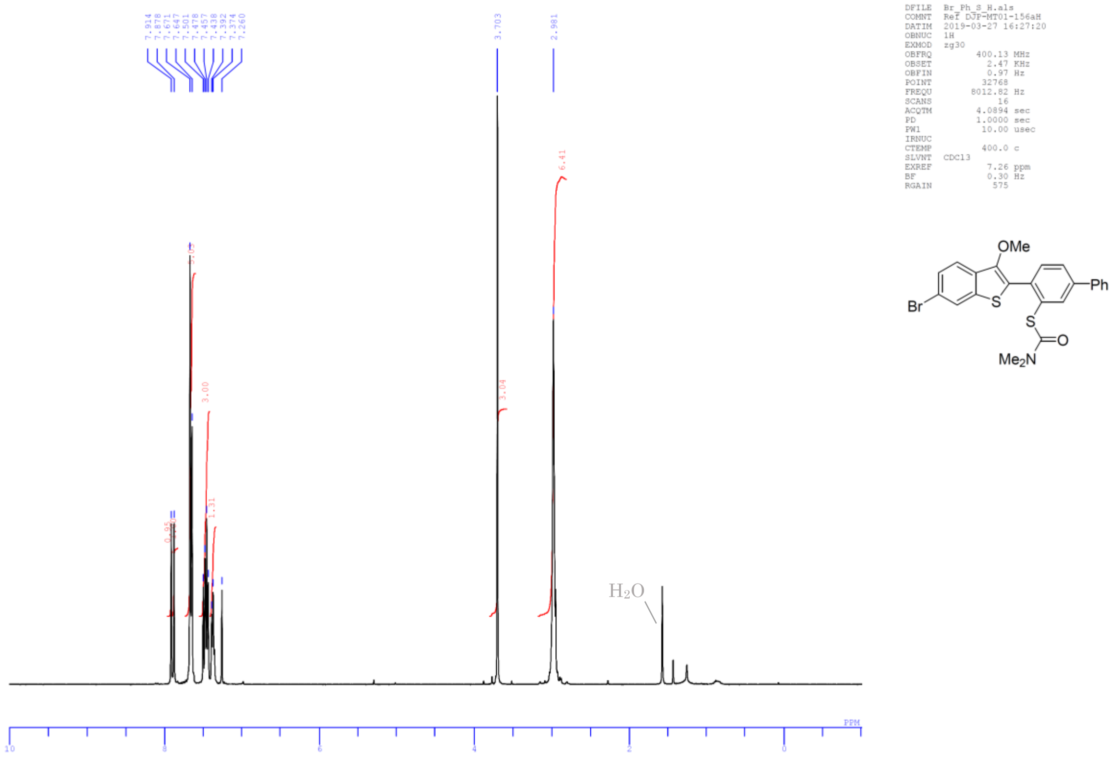

^{13}C NMR of 5bb (126 MHz, $CDCl_3$)

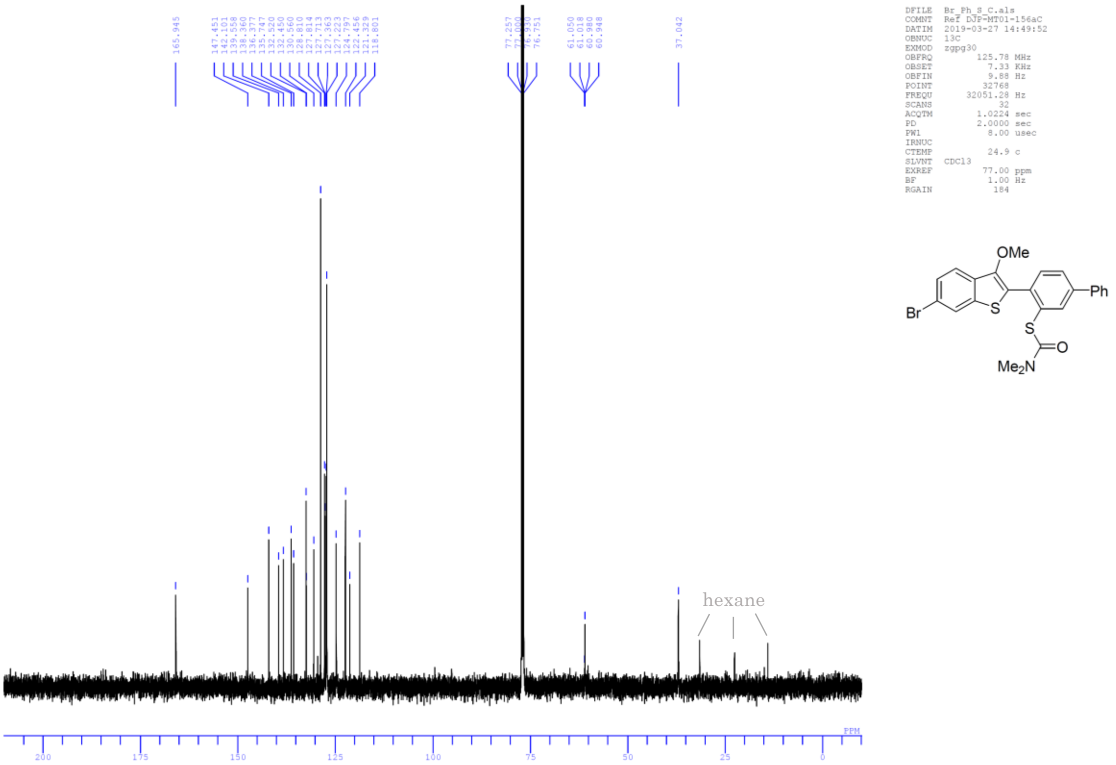

^{1}H NMR of 5bd (500 MHz, $CDCl_3$)

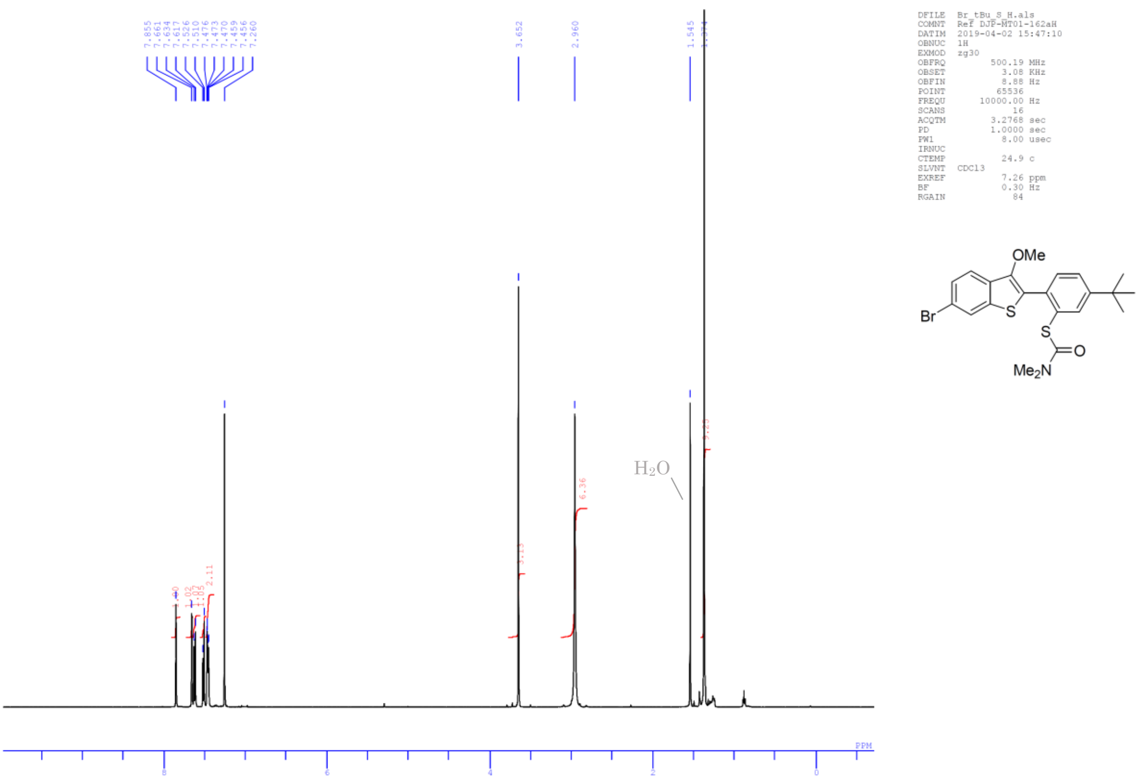

^{13}C NMR of 5bd (126 MHz, $CDCl_3$)

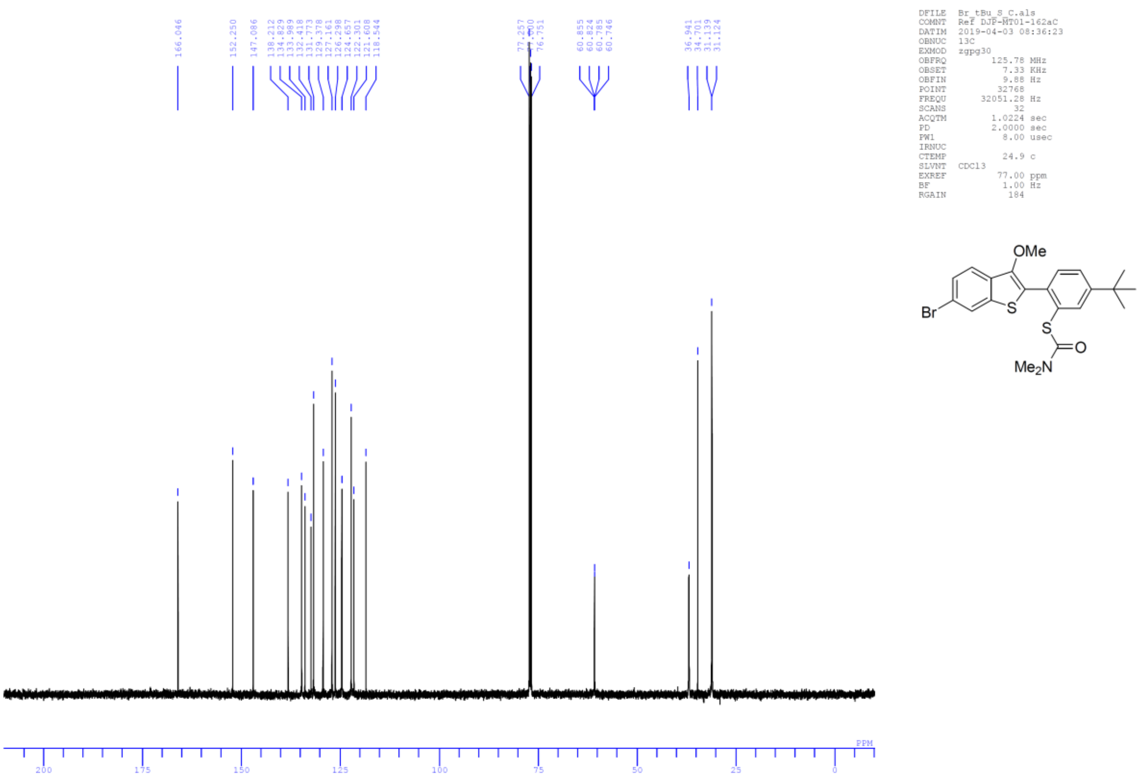

^{1}H NMR of 5bf (400 MHz, $CDCl_3$)

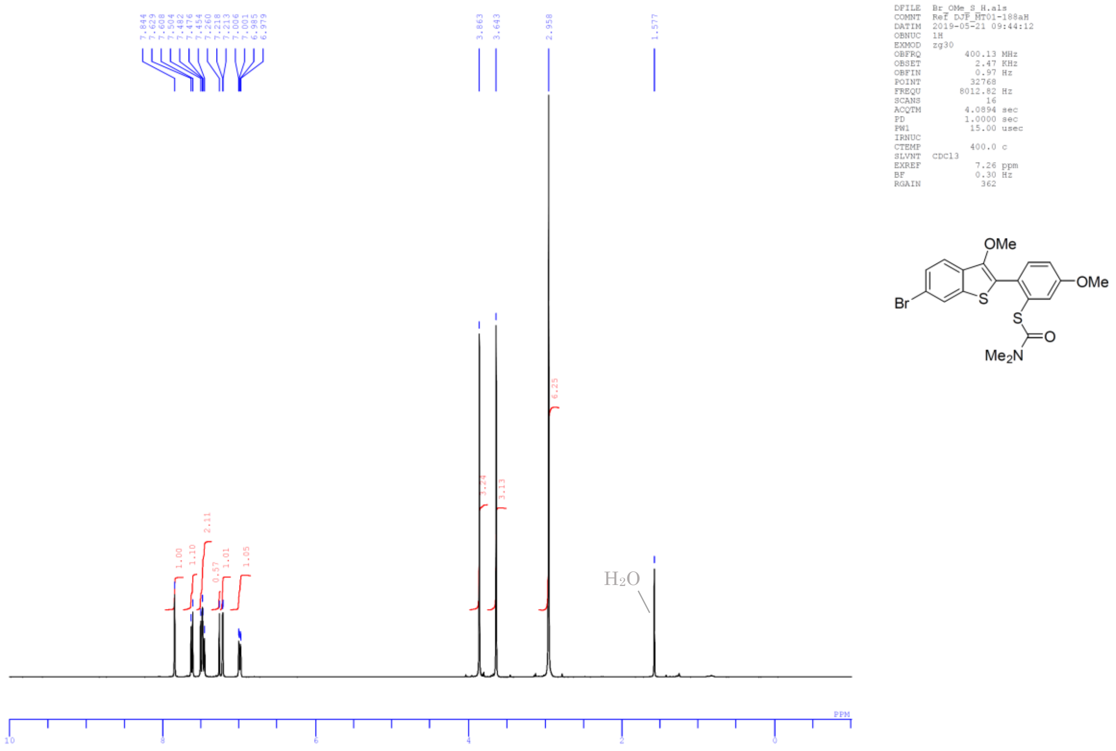

^{13}C NMR of 5bf (126 MHz, $CDCl_3$)

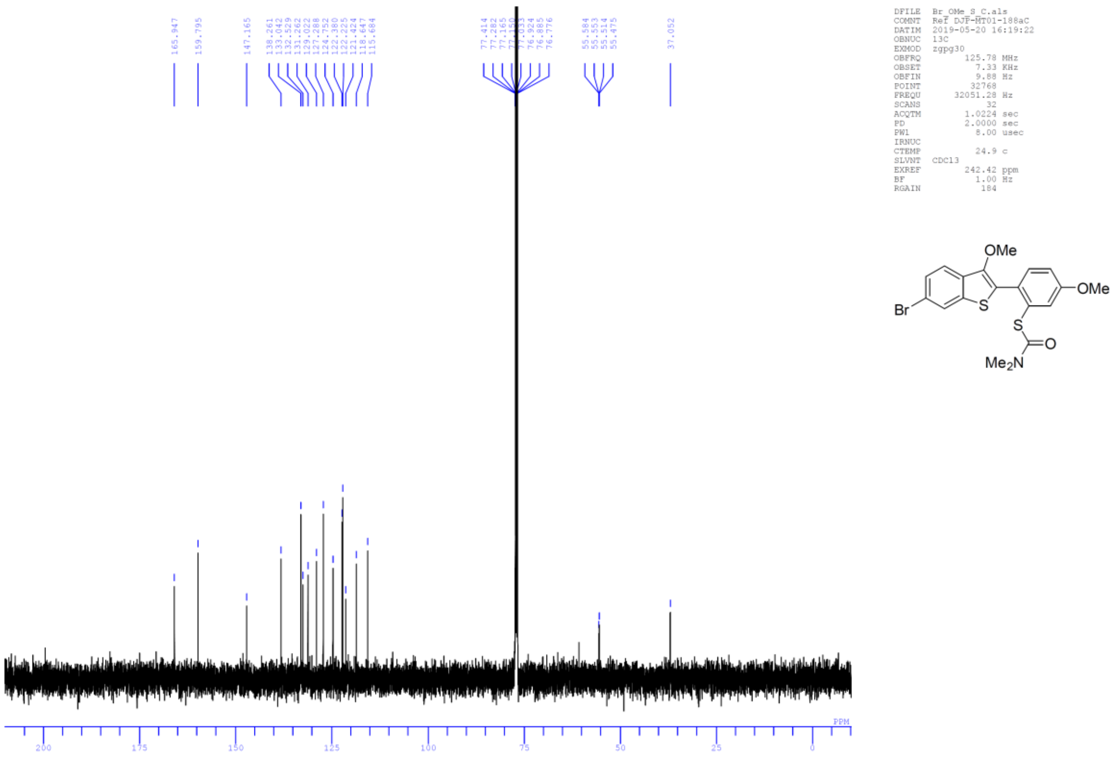

^{1}H NMR of 5bg (400 MHz, $CDCl_3$)

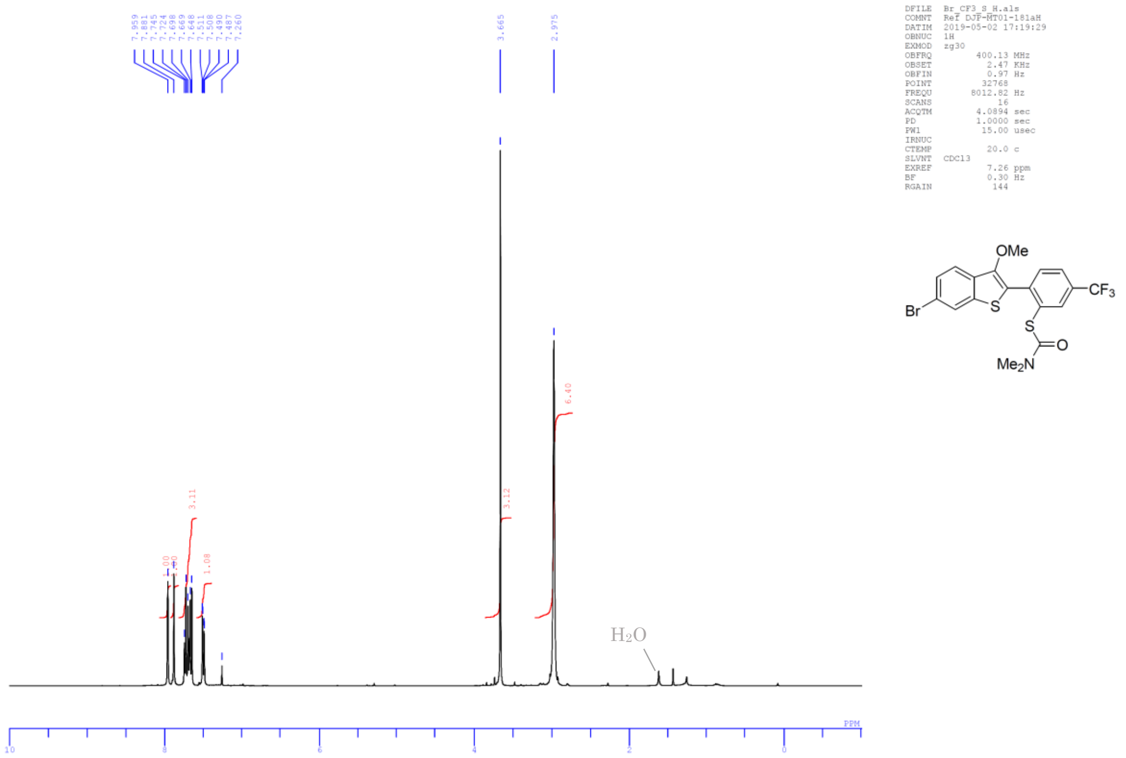

^{13}C NMR of 5bg (101 MHz, $CDCl_3$)

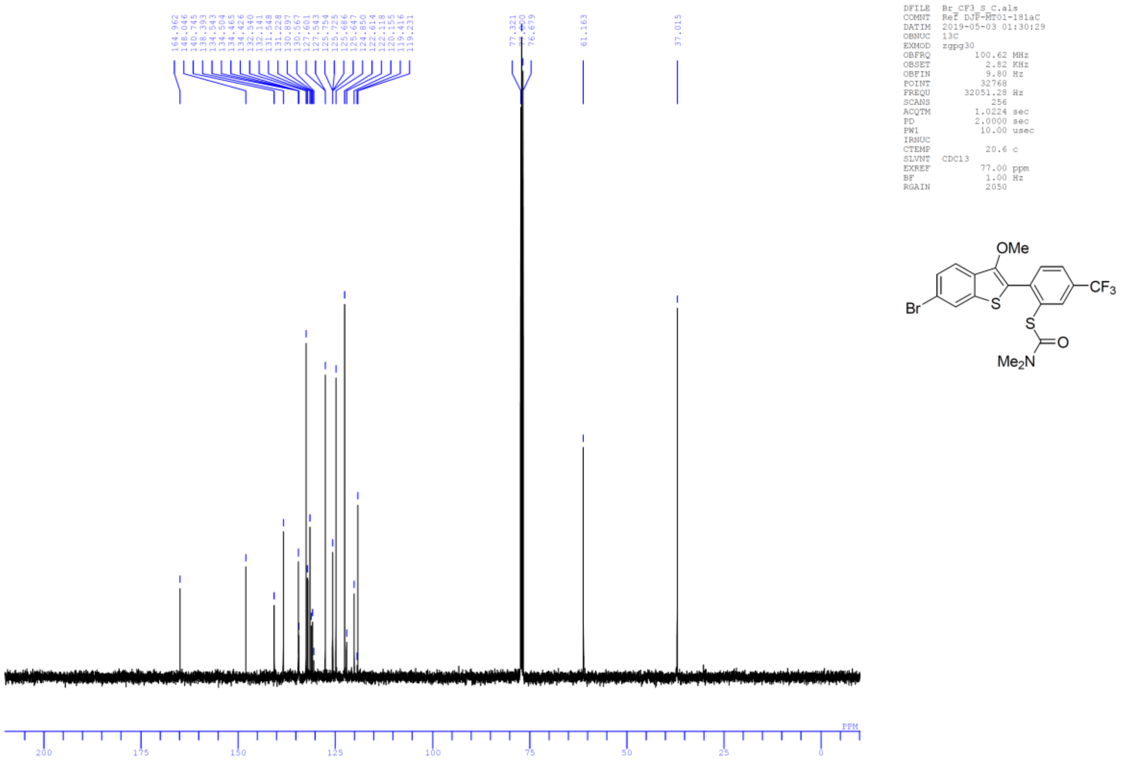

^{1}H NMR of 5cd (400 MHz, $CDCl_3$)

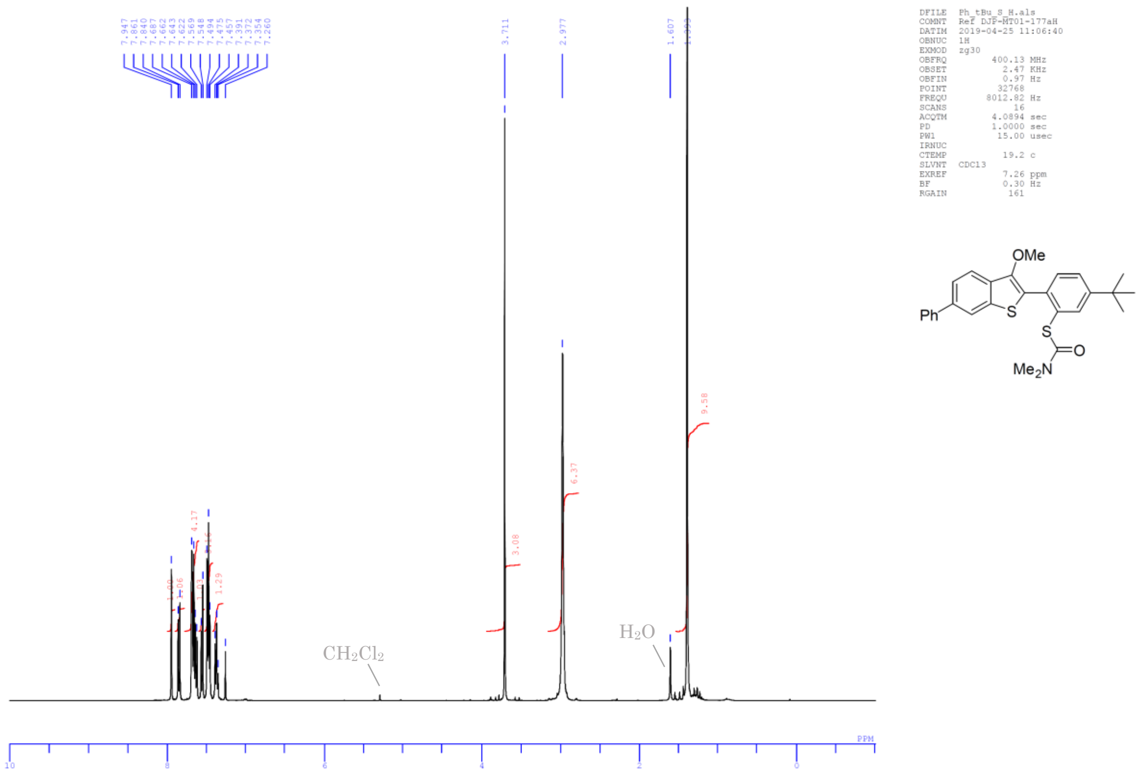

^{13}C NMR of 5cd (101 MHz, $CDCl_3$)

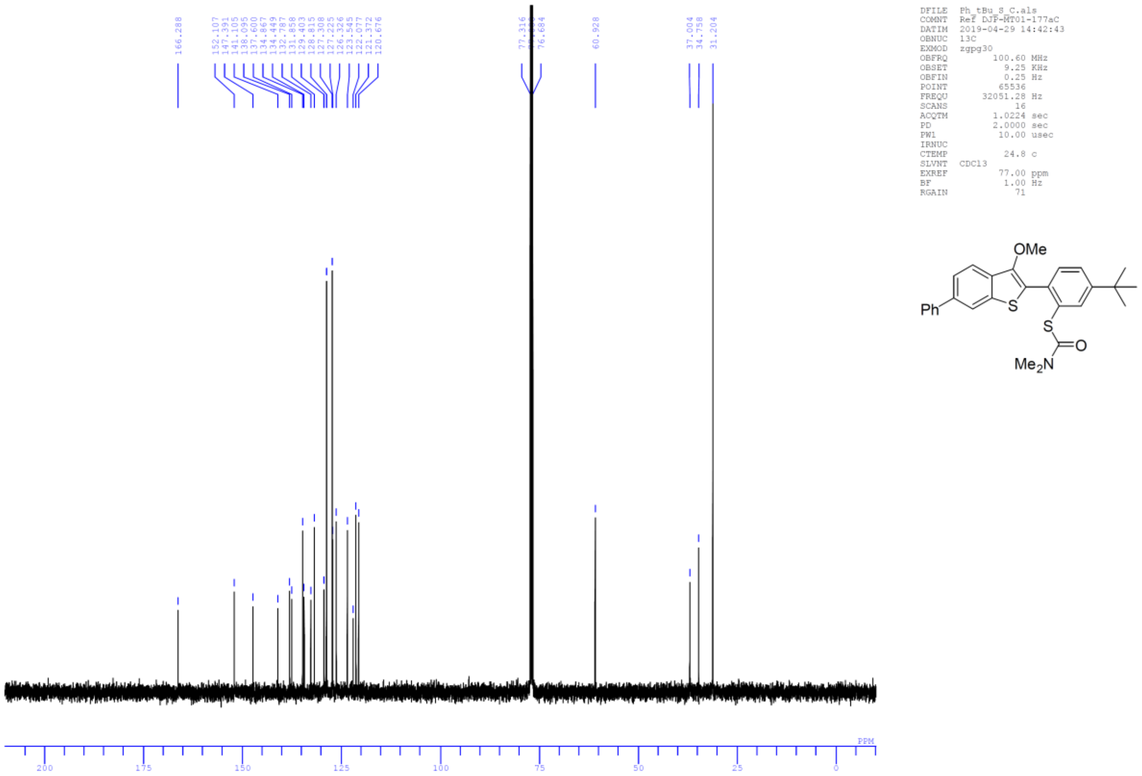

^{1}H NMR of 5ce (400 MHz, $CDCl_3$)

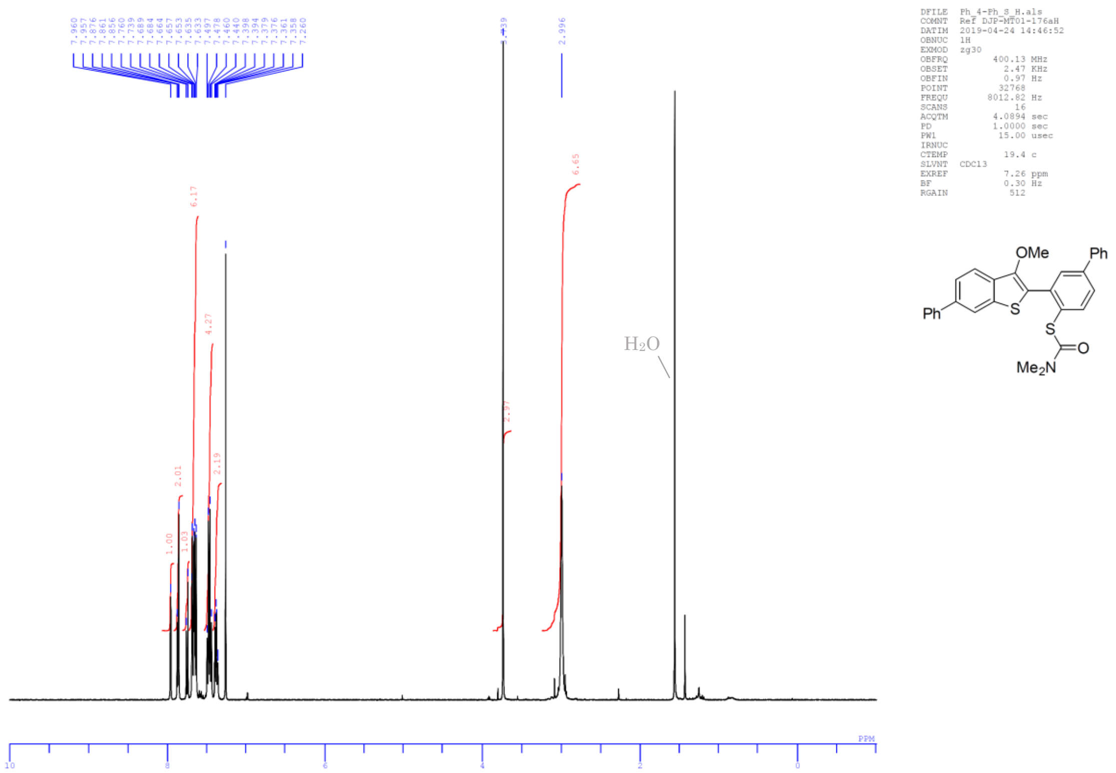

^{13}C NMR of 5ce (126 MHz, $CDCl_3$)

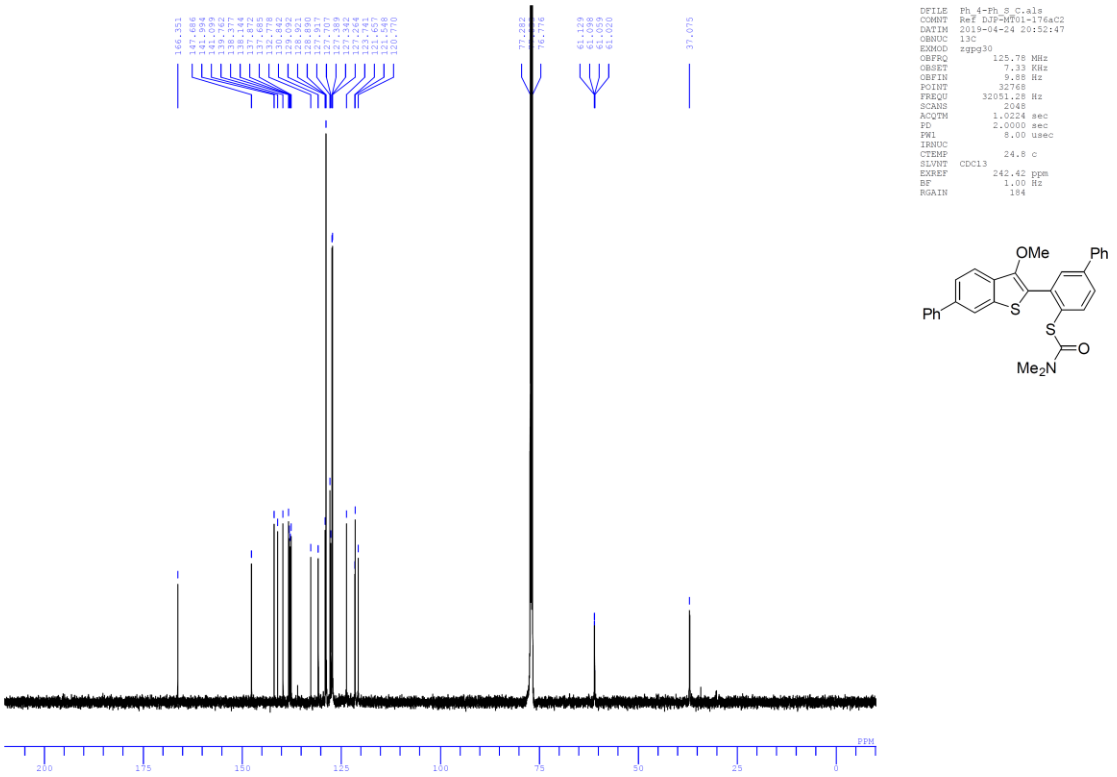

^{1}H NMR of 5eb (400 MHz, $CDCl_3$)

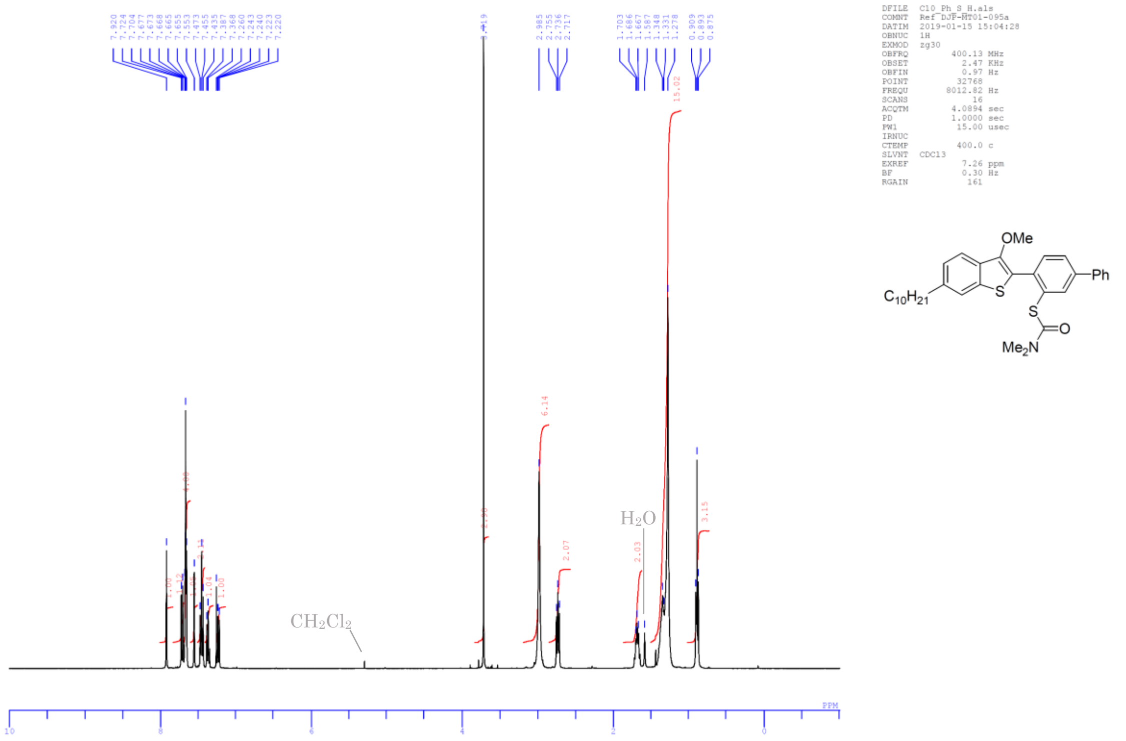

^{13}C NMR of 5eb (101 MHz, $CDCl_3$)

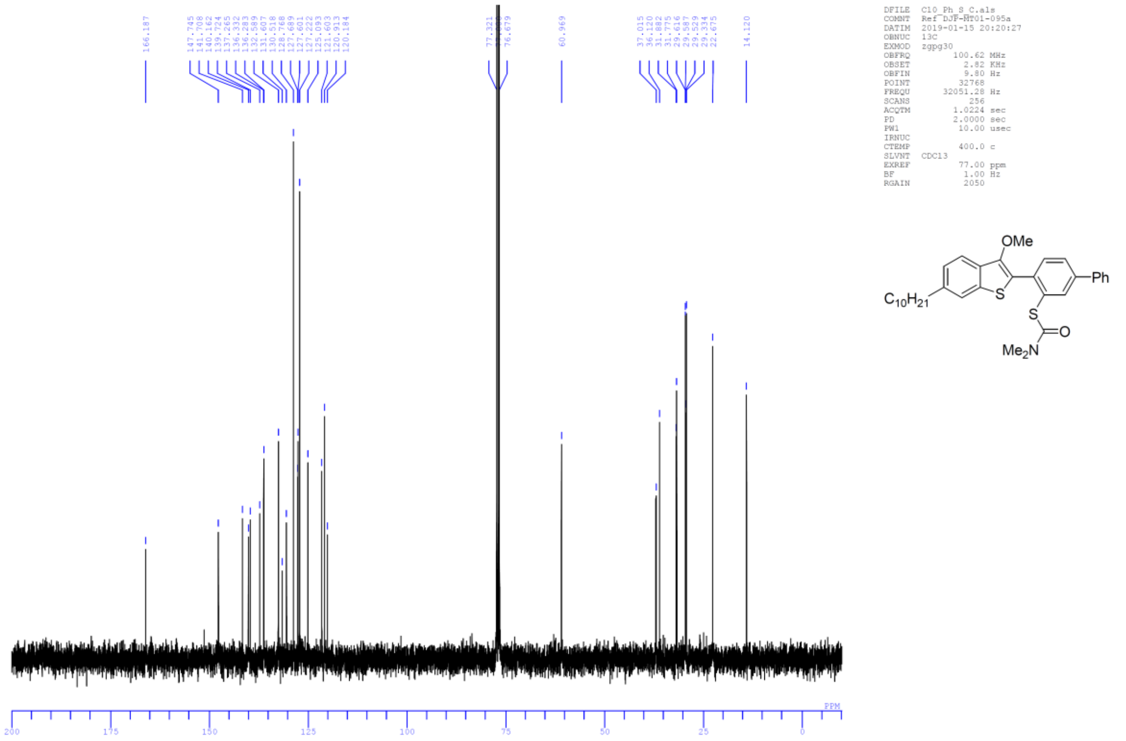

^{1}H NMR of 5eb’ (500 MHz, $CDCl_3$)

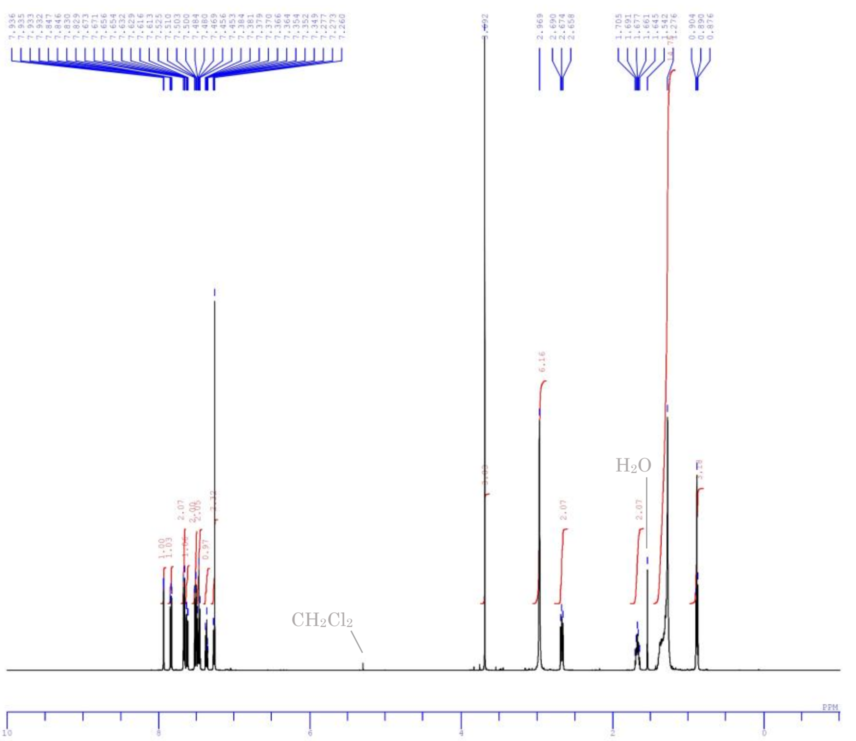

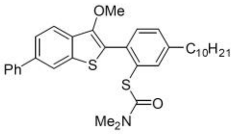

^{13}C NMR of 5eb’ (126 MHz, $CDCl_3$)

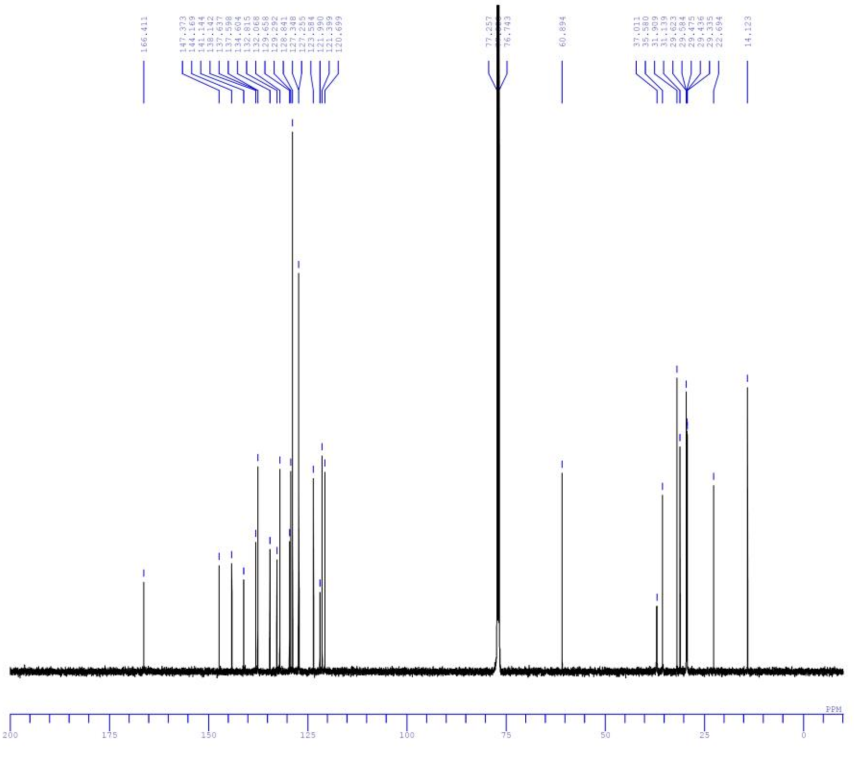

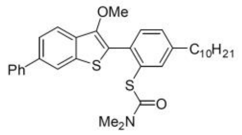

^{1}H NMR of 5ef (500 MHz, $CDCl_3$)

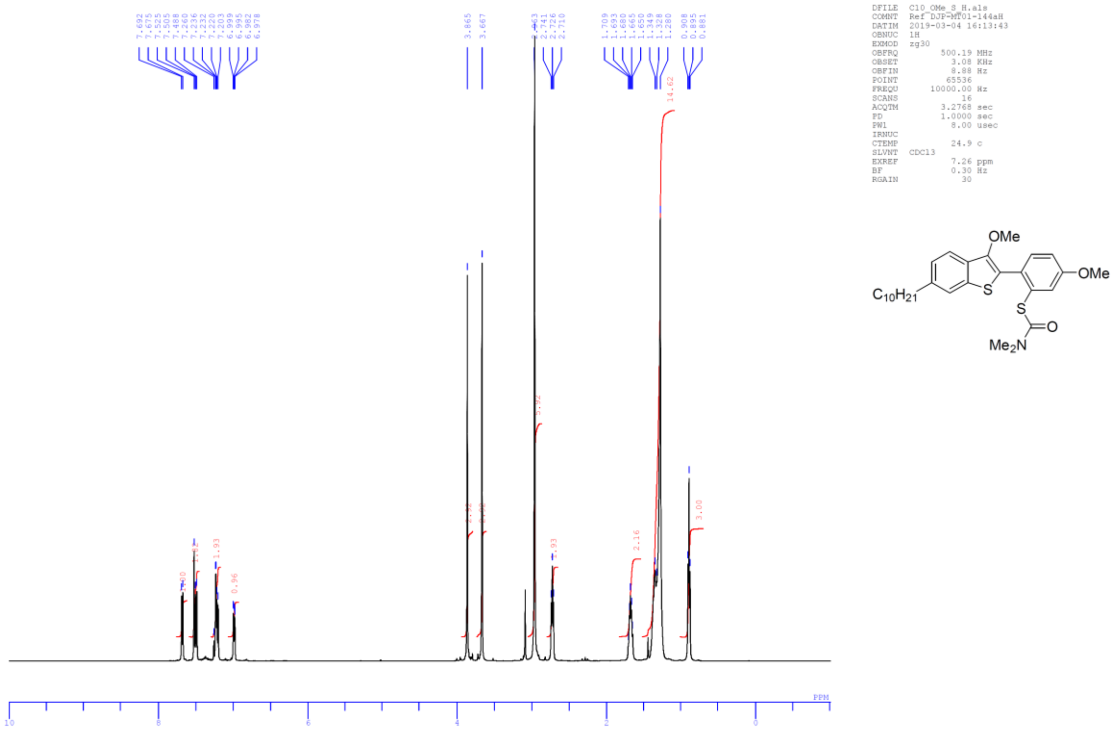

^{13}C NMR of 5ef (126 MHz, $CDCl_3$)

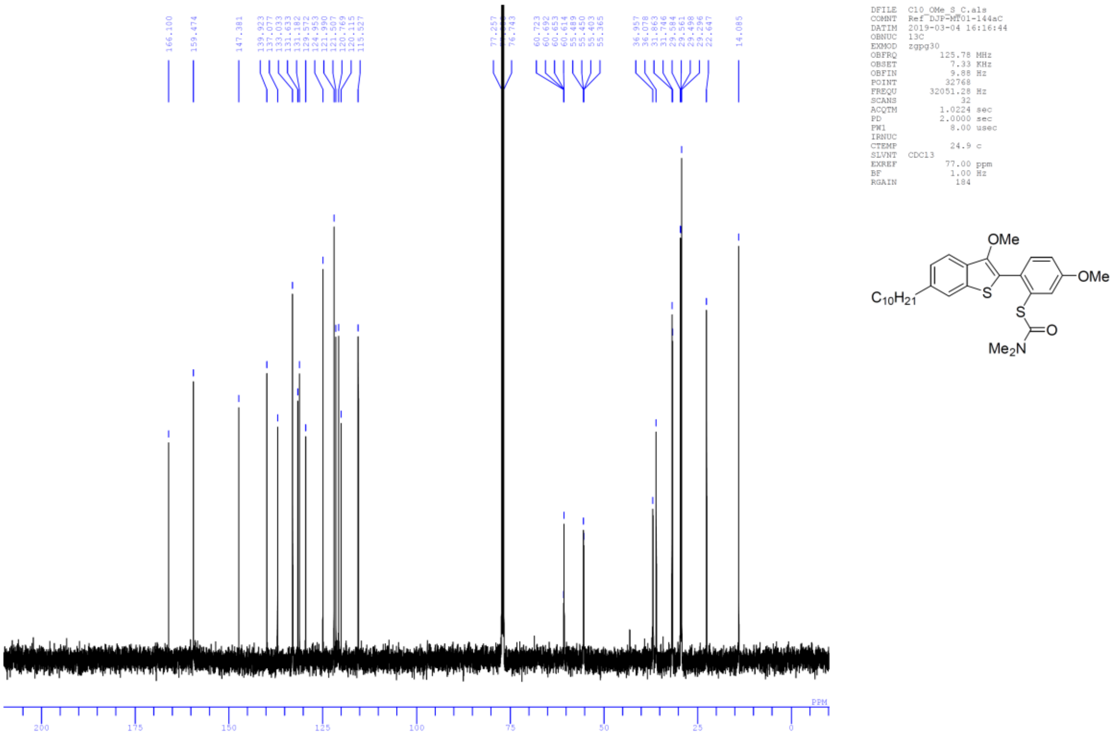

^{1}H NMR of 5eg (400 MHz, $CDCl_3$)

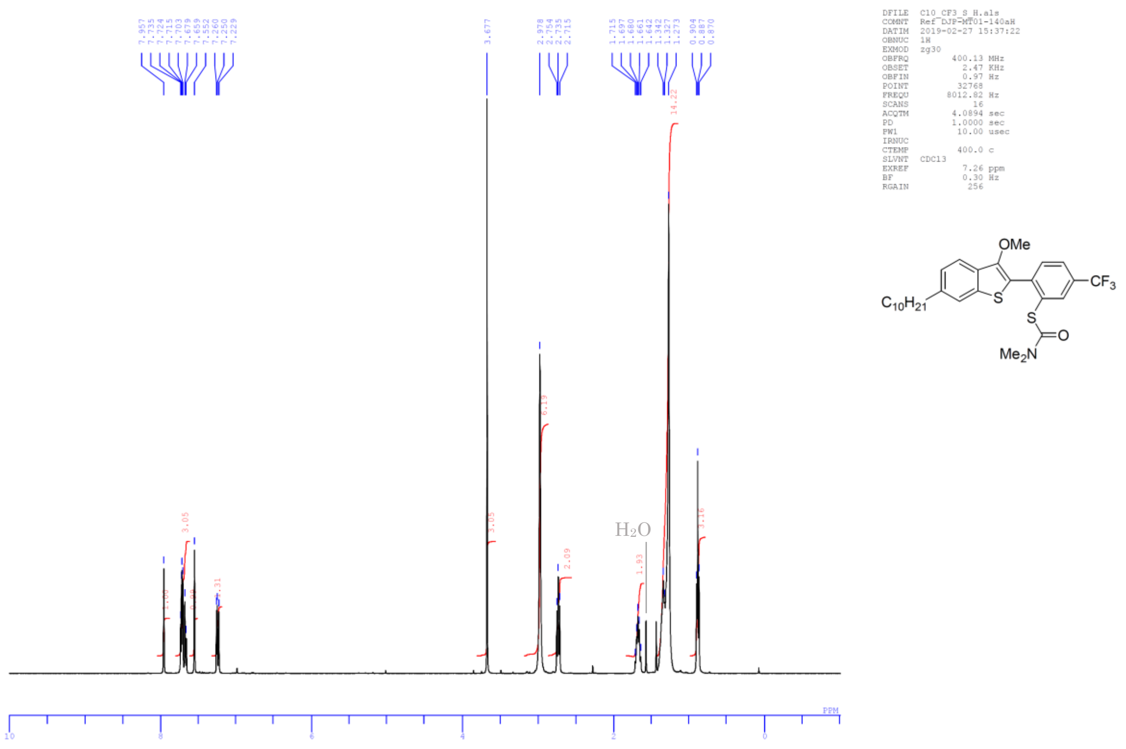

^{13}C NMR of 5eg (126 MHz, $CDCl_3$)

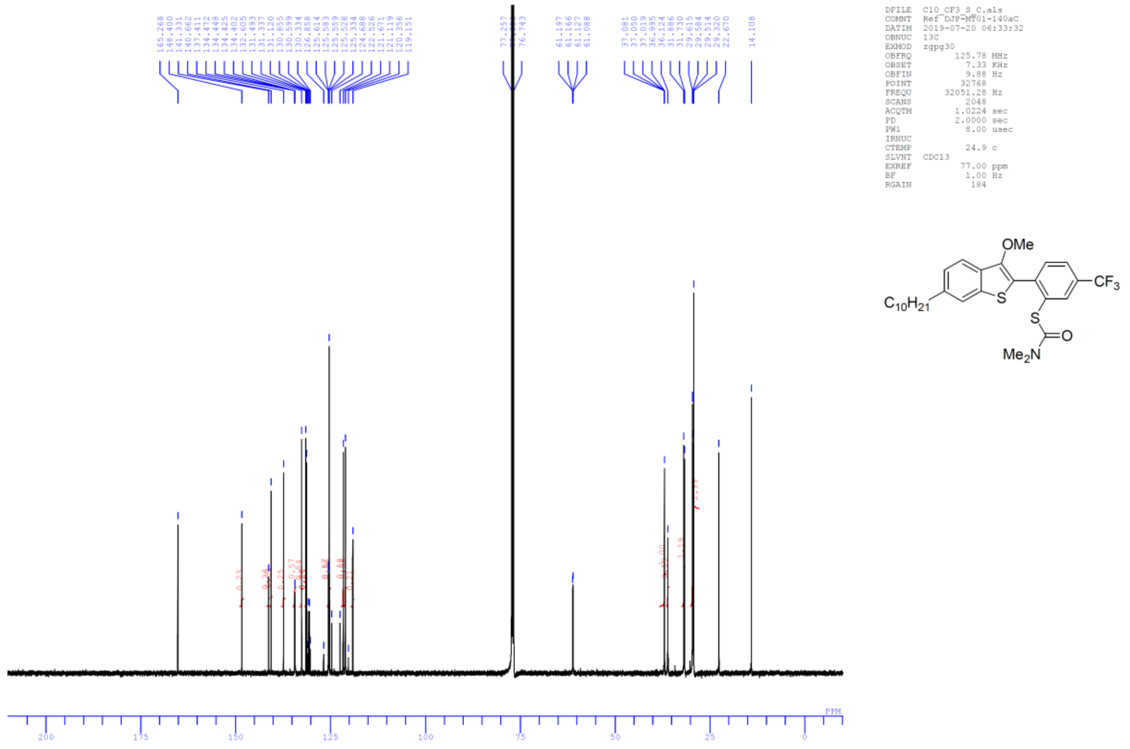

^{1}H NMR of 5eh (400 MHz, $CDCl_3$)

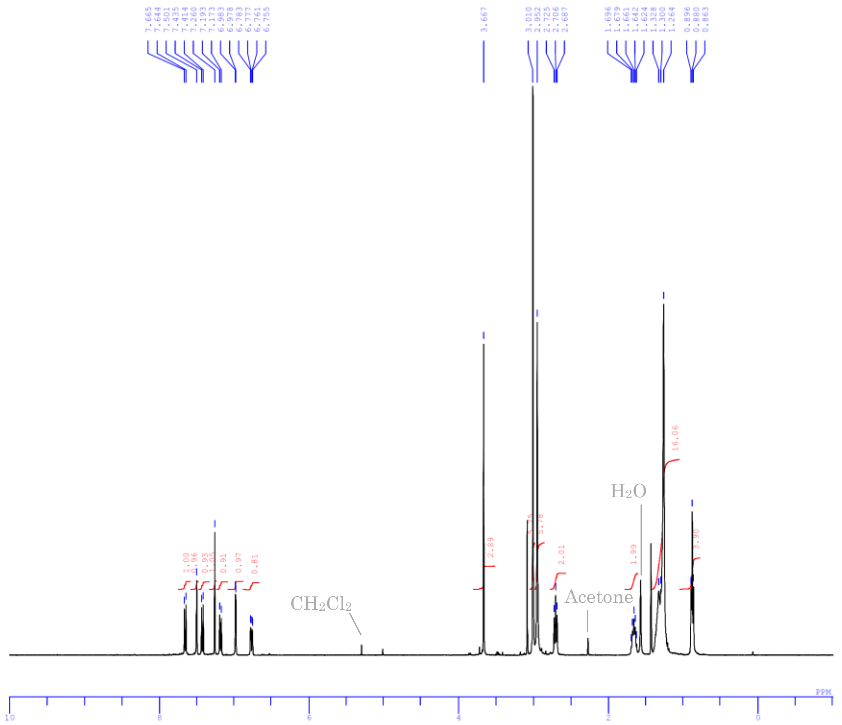

DFILE C10_NMe2_S_H.als
COMNT Ref DJP-MT01-150aH
DATIM 2019-03-08 16:49:12
OBNUC 1H
EXMOD zg30
OBFRQ 400.13 MHz
OBSET 2.47 KHz
OBFIN 0.97 Hz
POINT 32768
FREQU 8012.82 Hz
SCANS 16
ACQTM 4.0894 sec
PD 1.0000 sec
PW1 10.00 usec
IRNUC
CTEMP 400.0 c
SLVNT CDCl3
EXREF 7.26 ppm
BF 0.30 Hz
RGAIN 575

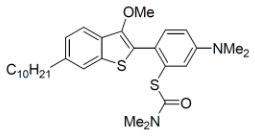

^{13}C NMR of 5eh (126 MHz, $CDCl_3$)

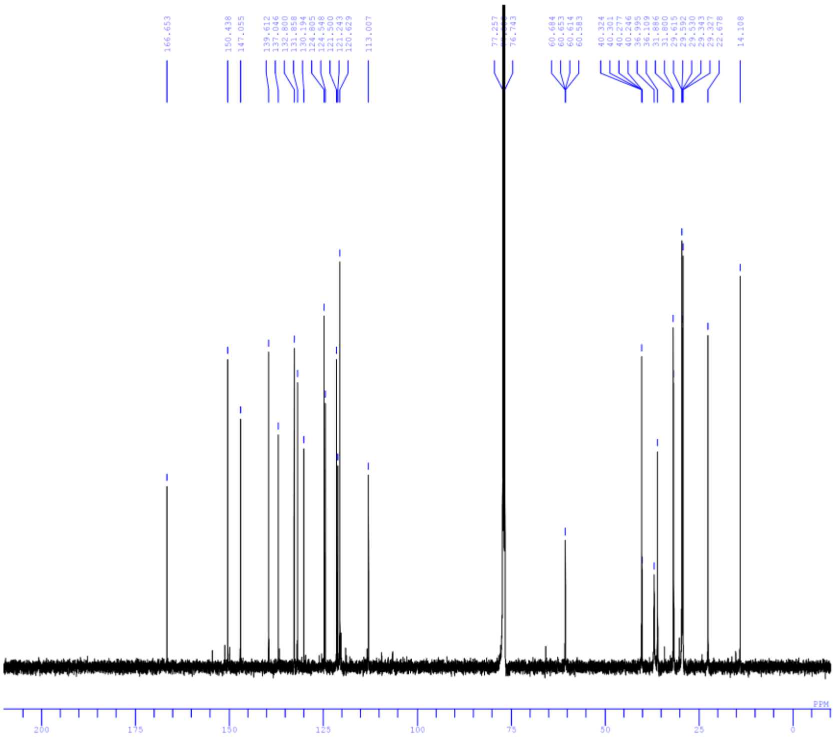

DFILE C10_NMe2_S_C.als
COMNT Ref DJP-MT C10NMe2SC2048
DATIM 2019-07-18 20:43:32
OBNUC 13C
EXMOD zgpg30
OBFRQ 125.78 MHz
OBSET 7.33 KHz
OBFIN 9.88 Hz
POINT 32768
FREQU 32051.28 Hz
SCANS 2048
ACQTM 1.0224 sec
PD 2.0000 sec
PW1 8.00 usec
IRNUC
CTEMP 24.9 c
SLVNT CDCl3
EXREF 77.00 ppm
BF 1.00 Hz
RGAIN 184

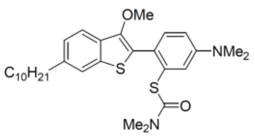

^{1}H NMR of 4aa (500 MHz, $CDCl_3$)

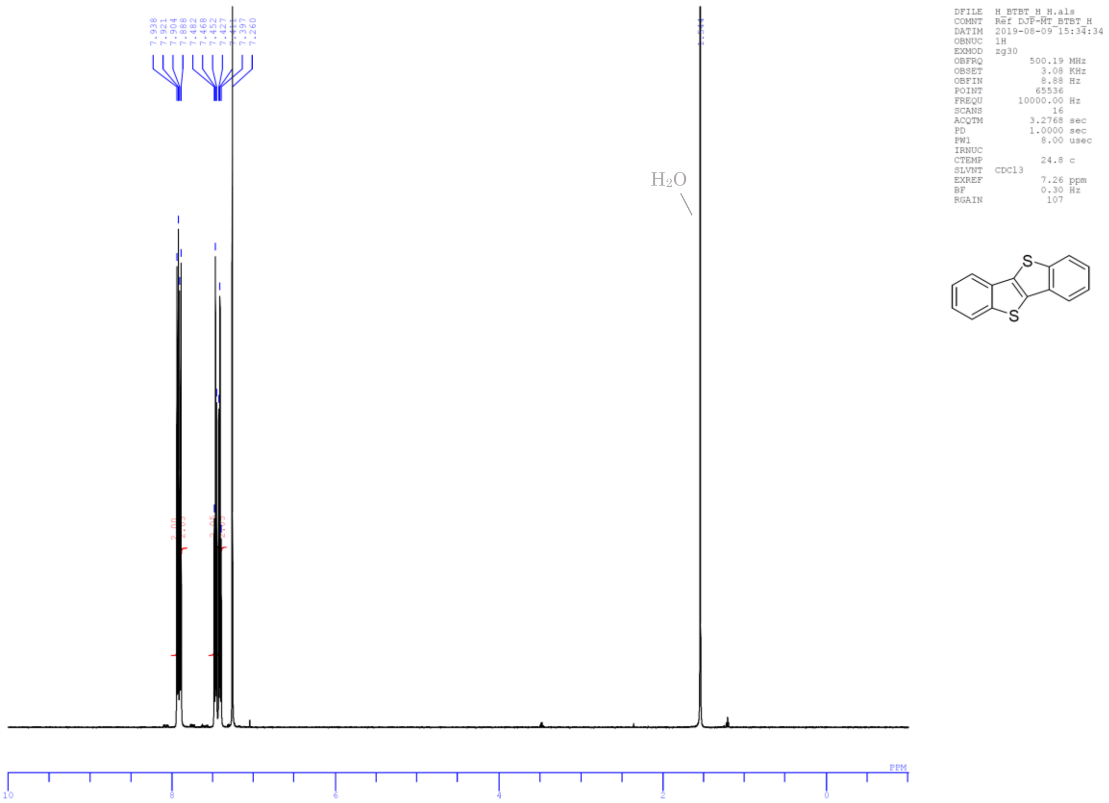

^{13}C NMR of 4aa (126 MHz, $CDCl_3$)

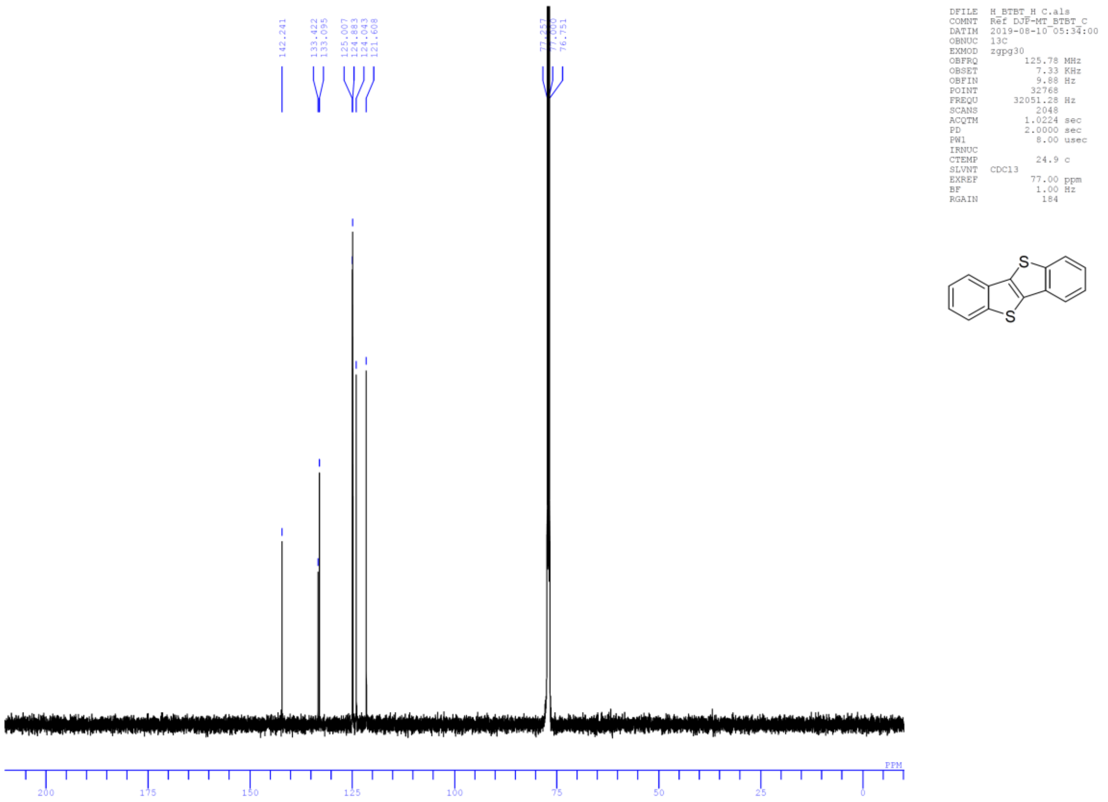

^{1}H NMR of 4ab (400 MHz, $CDCl_3$)

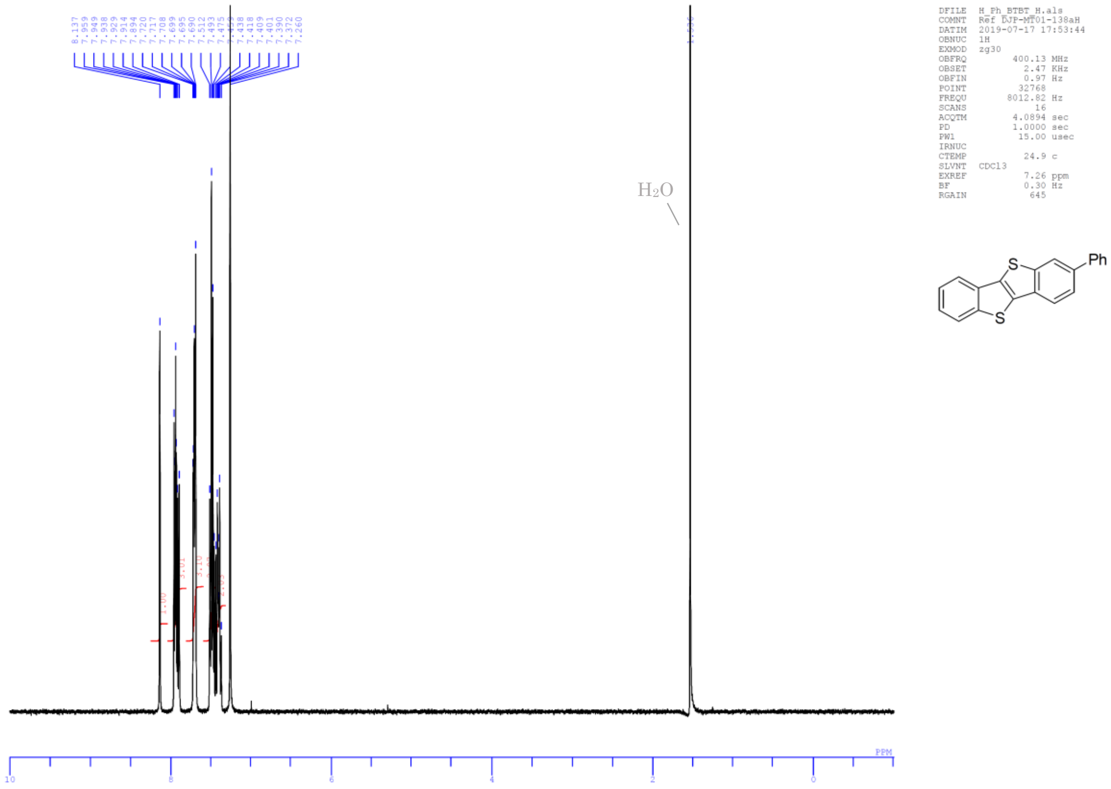

^{13}C NMR of 4ab (126 MHz, $CDCl_3$)

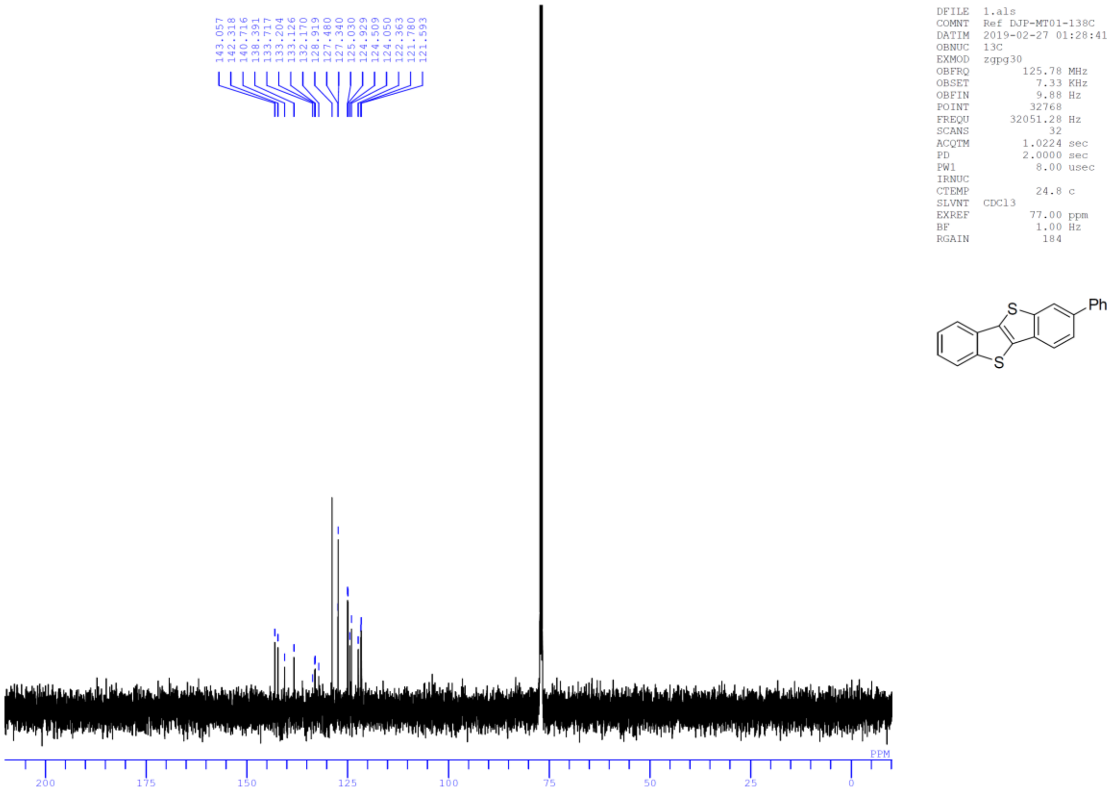

^{1}H NMR of 4ac (400 MHz, $CDCl_3$)

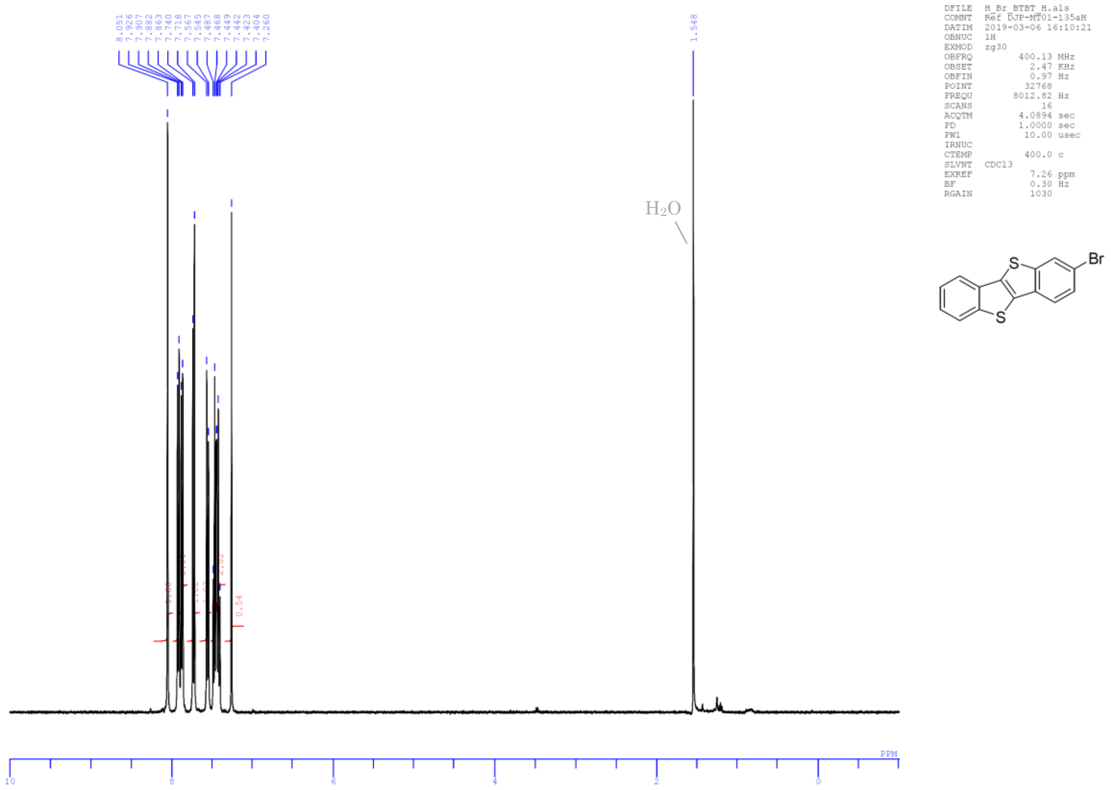

^{13}C NMR of 4ac (101 MHz, $CDCl_3$)

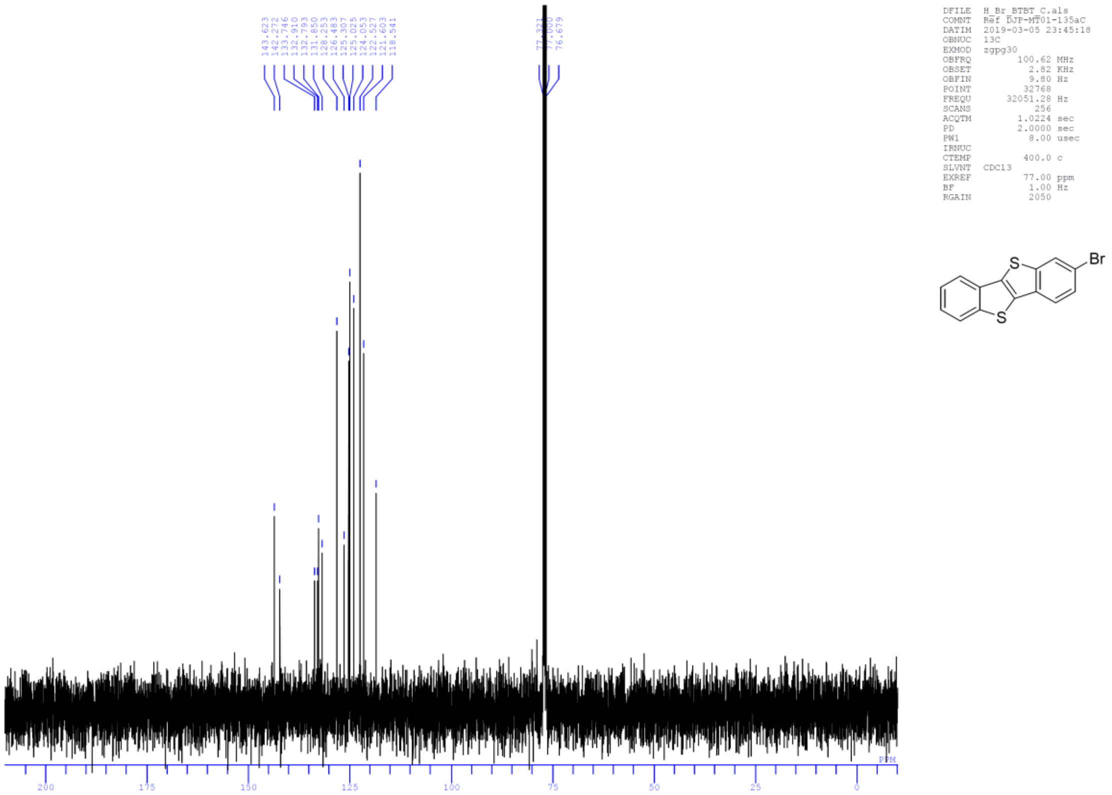

^{1}H NMR of 4ad (400 MHz, CDCl$_3$)

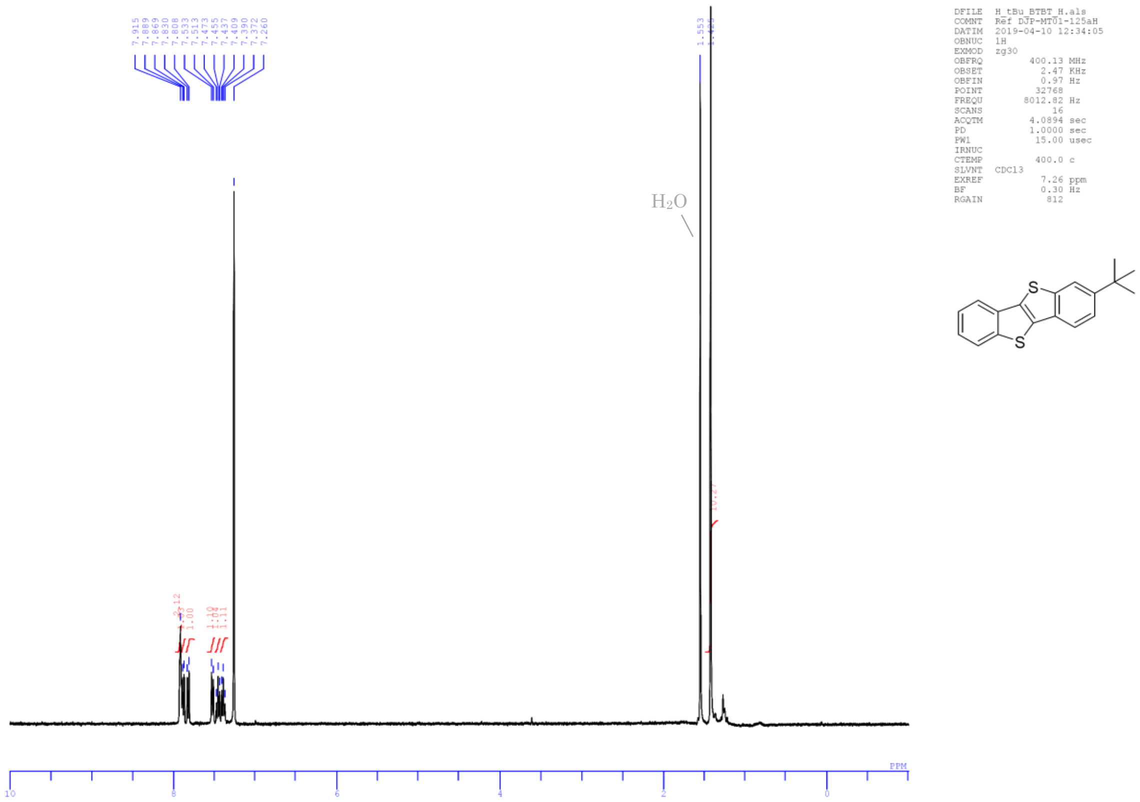

^{13}C NMR of 4ad (126 MHz, CDCl$_3$)

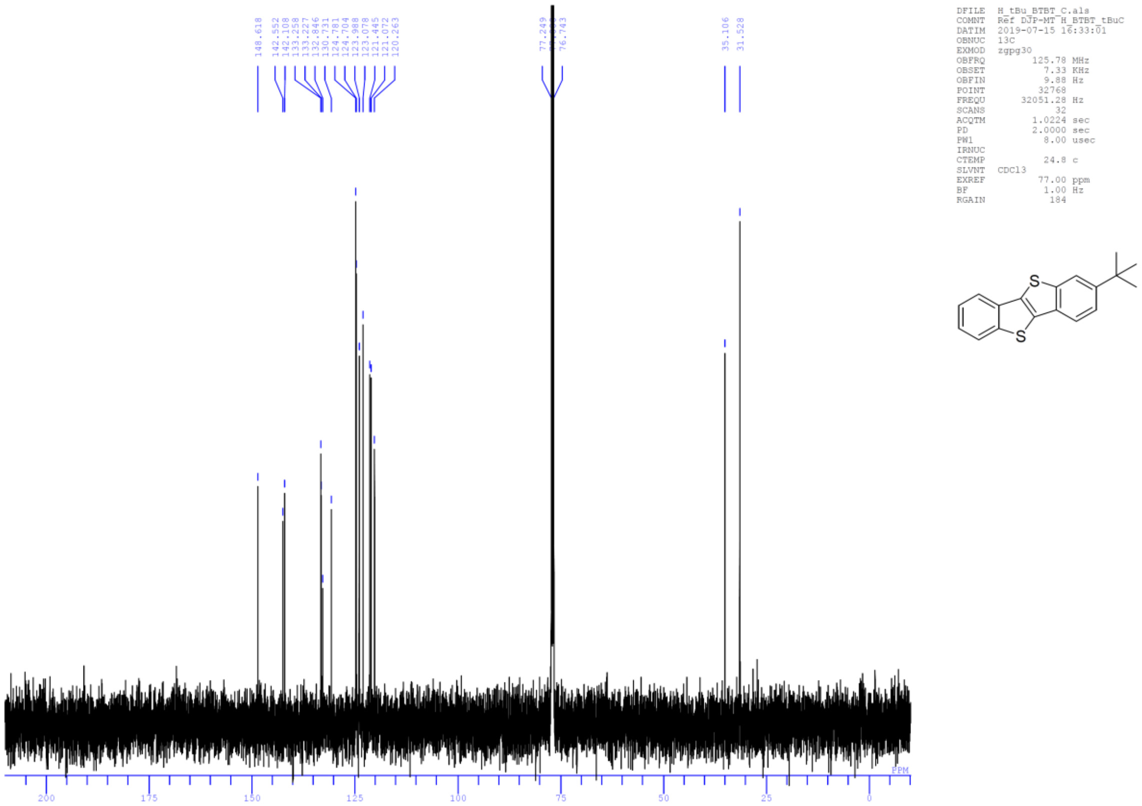

^{1}H NMR of 4ae (400 MHz, $CDCl_3$)

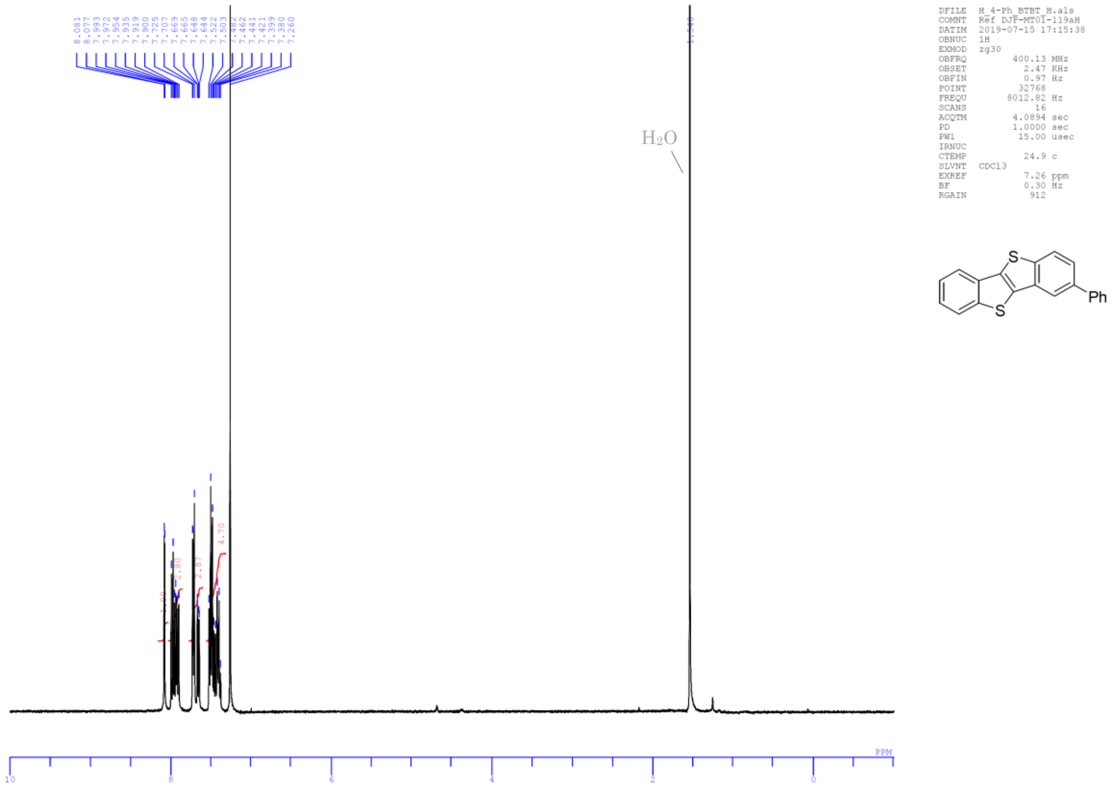

^{13}C NMR of 4ae (126 MHz, $CDCl_3$)

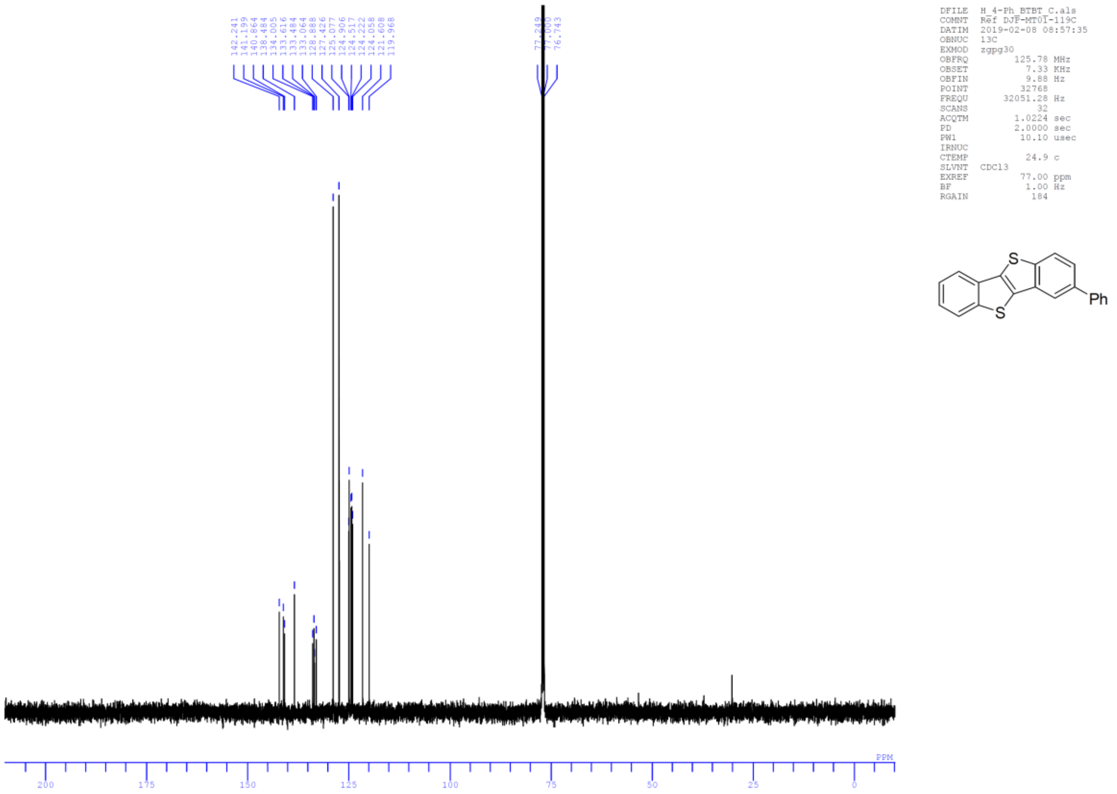

^{1}H NMR of 4bb (400 MHz, $CDCl_3$)

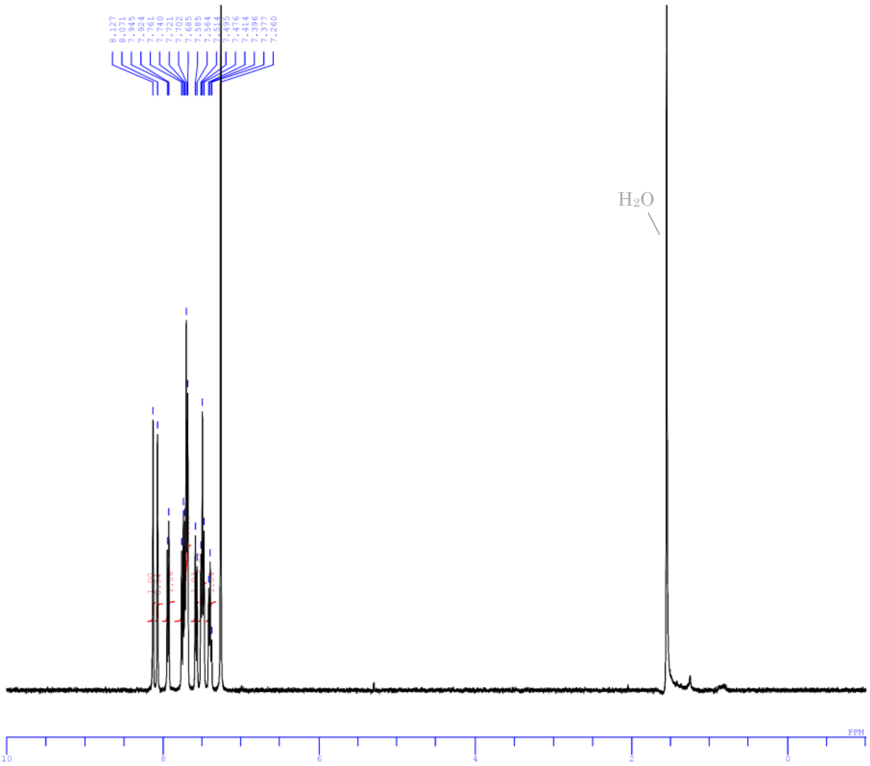

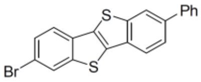

^{13}C NMR of 4bb (126 MHz, $CDCl_3$)

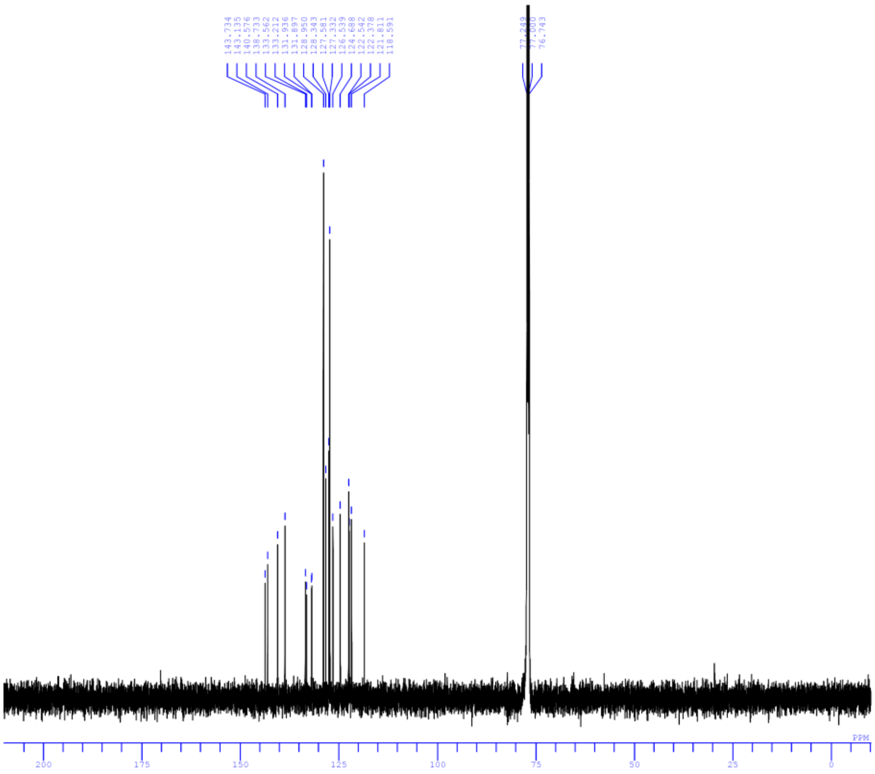

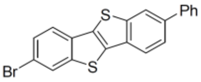

^{1}H NMR of 4bd (500 MHz, $CDCl_3$)

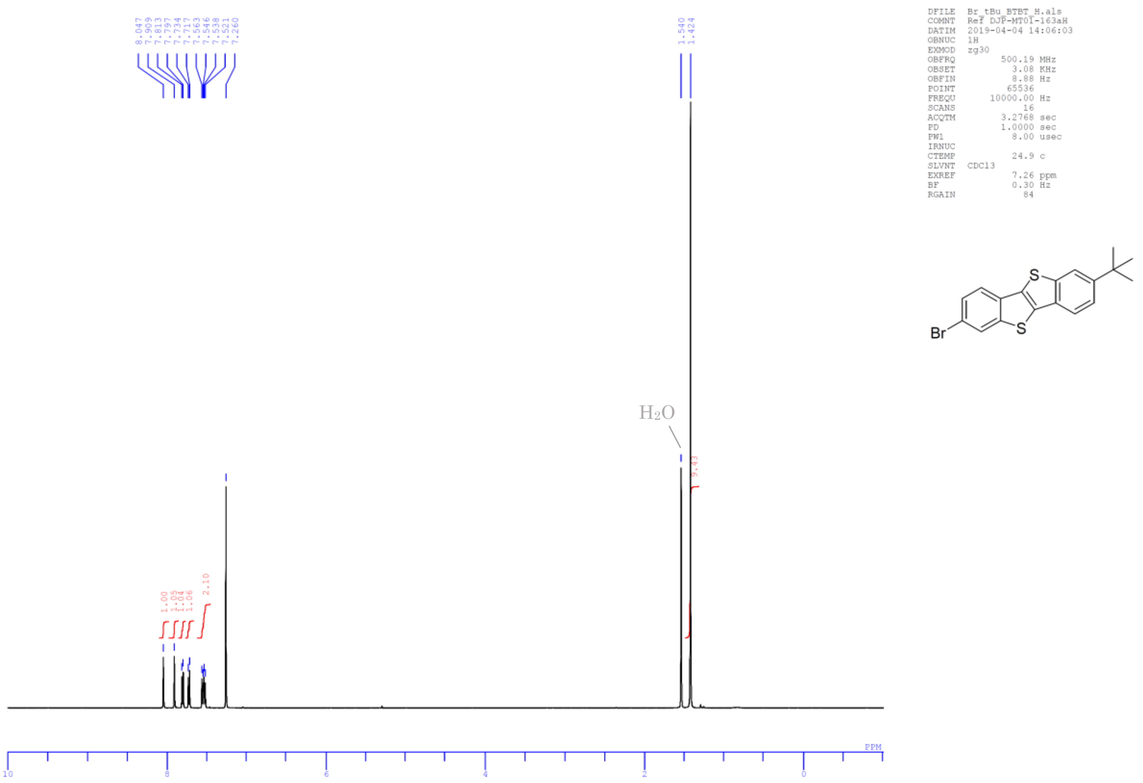

^{13}C NMR of 4bd (126 MHz, $CDCl_3$)

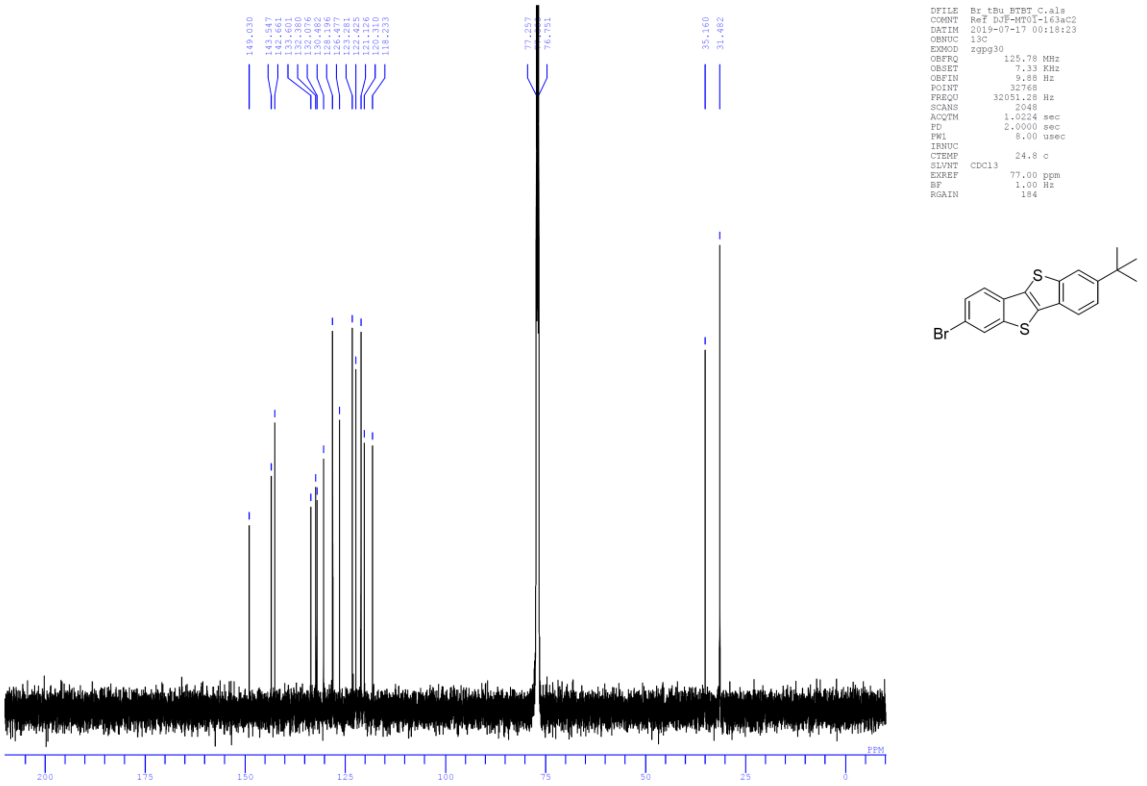

^{1}H NMR of 4bf (400 MHz, $CDCl_3$)

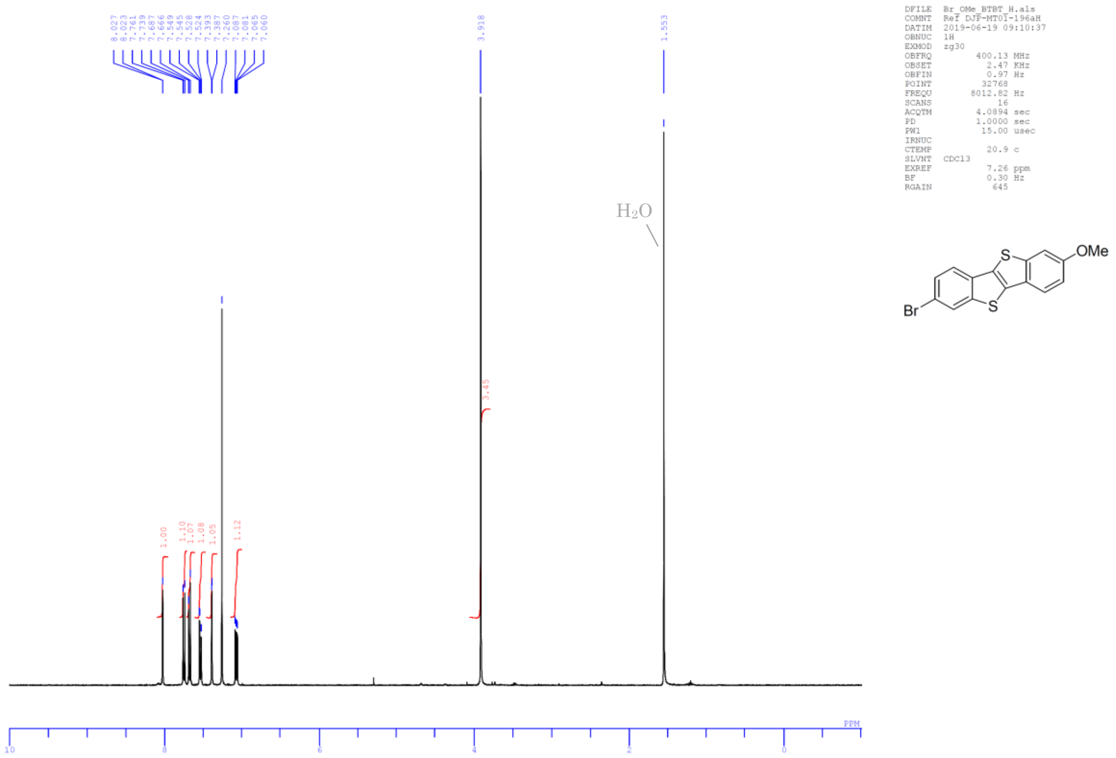

^{13}C NMR of 4bf (126 MHz, $CDCl_3$)

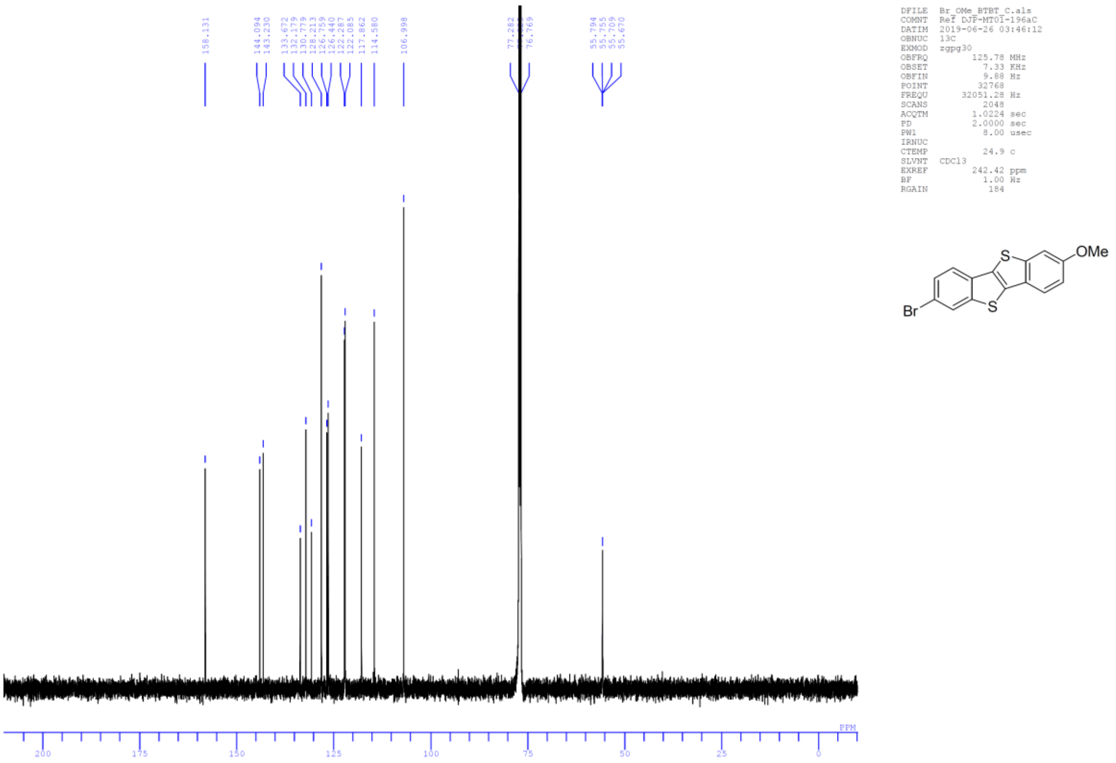

^{1}H NMR of 4bg (400 MHz, $CDCl_3$)

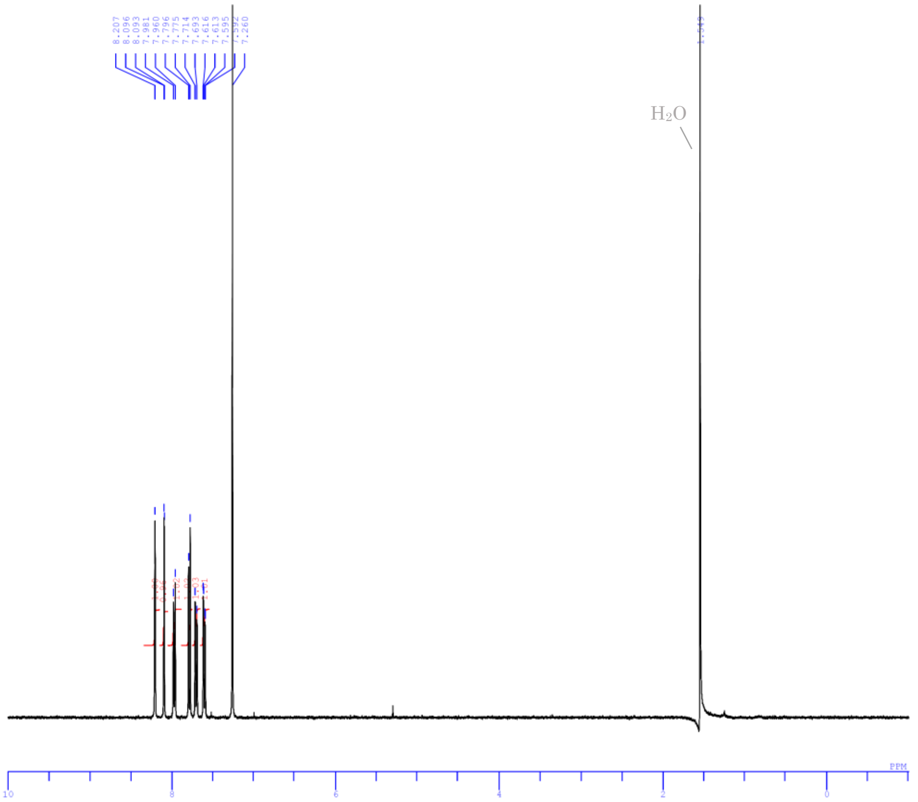

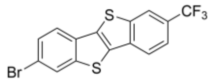

^{13}C NMR of 4bg (126 MHz, $CDCl_3$)

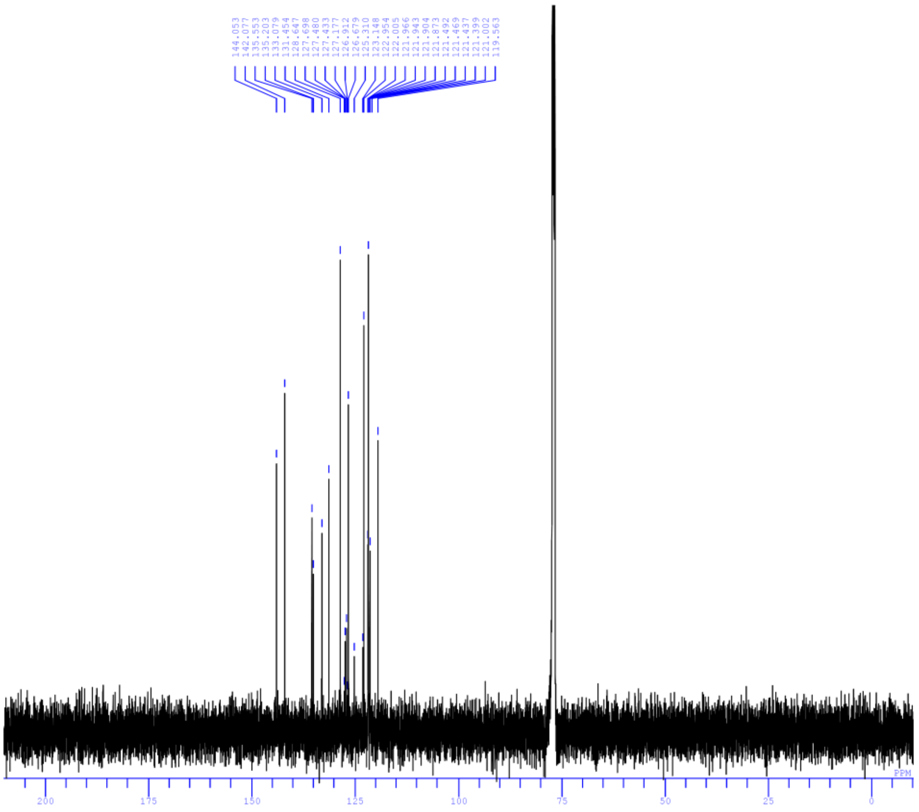

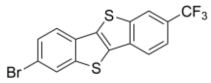

^{1}H NMR of 4cd (500 MHz, $CDCl_3$)

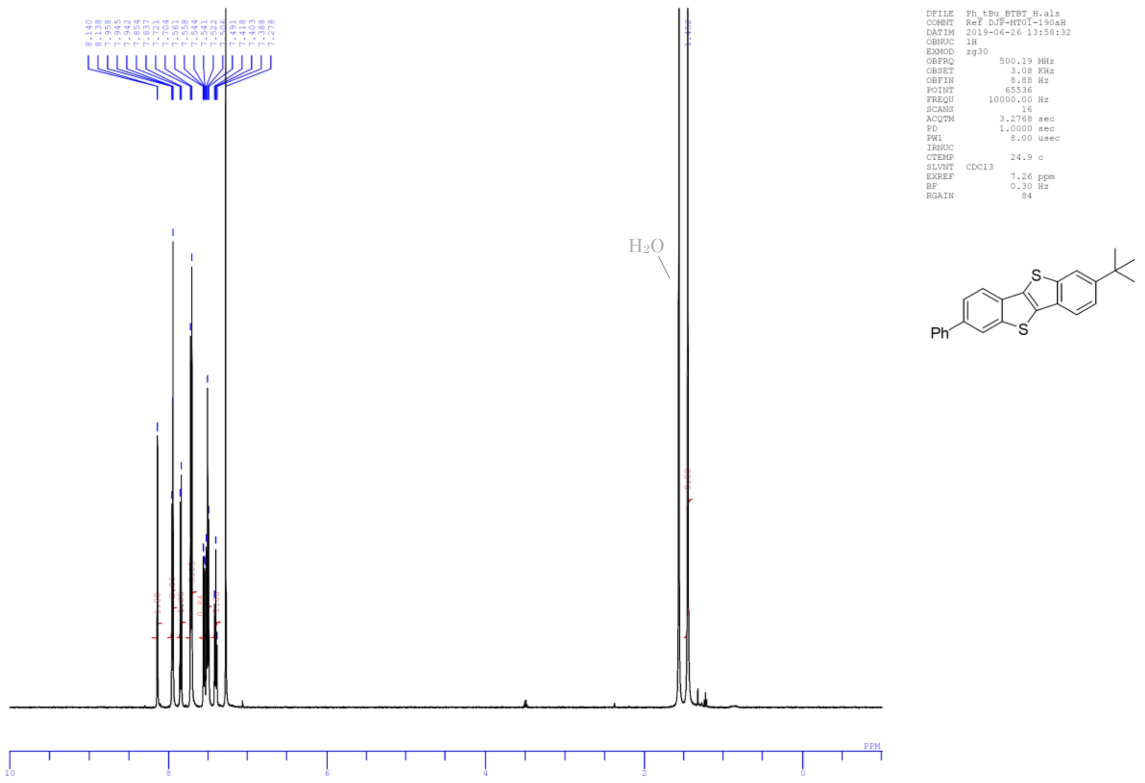

^{13}C NMR of 4cd (126 MHz, $CDCl_3$)

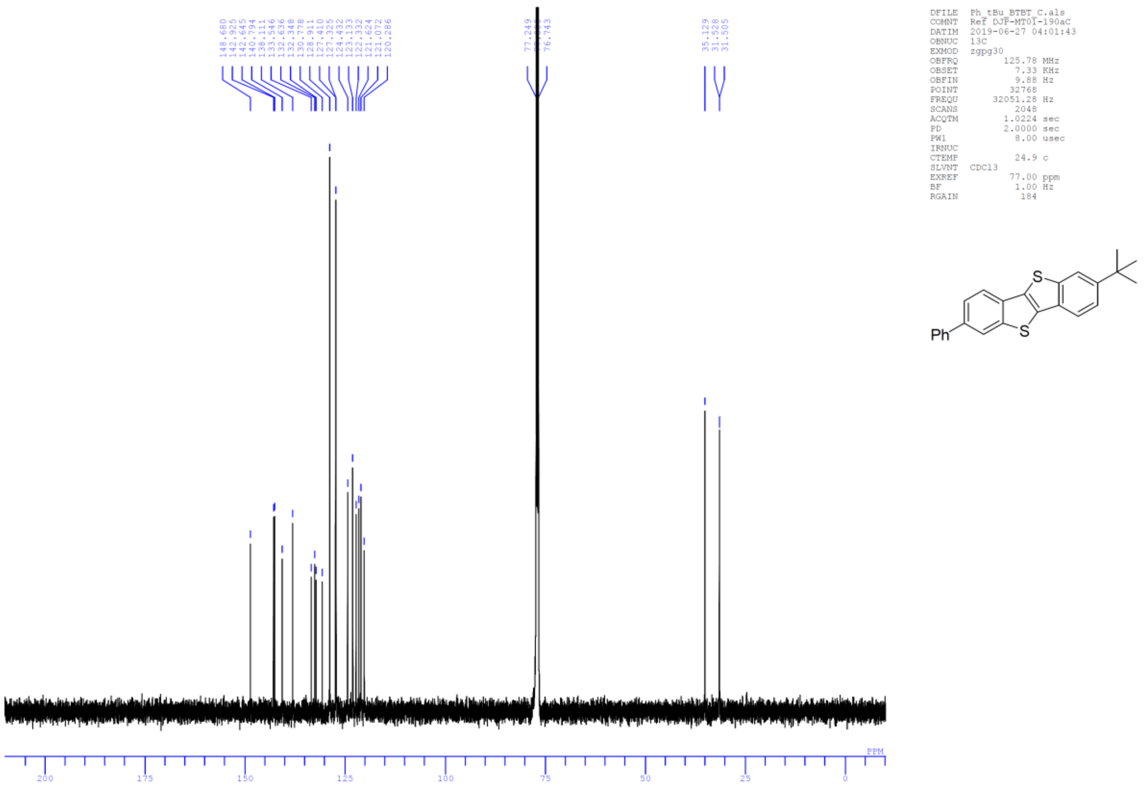

^1H NMR of 4ce (500 MHz, $CDCl_3$)

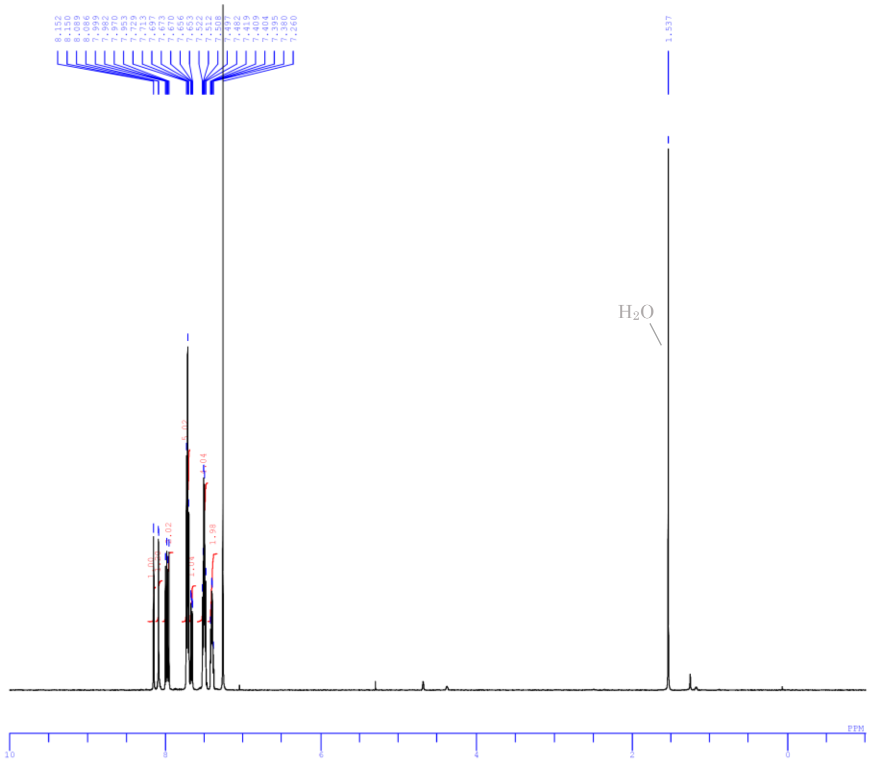

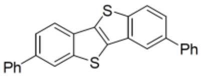

^{13}C NMR of 4ce (126 MHz, $CDCl_3$)

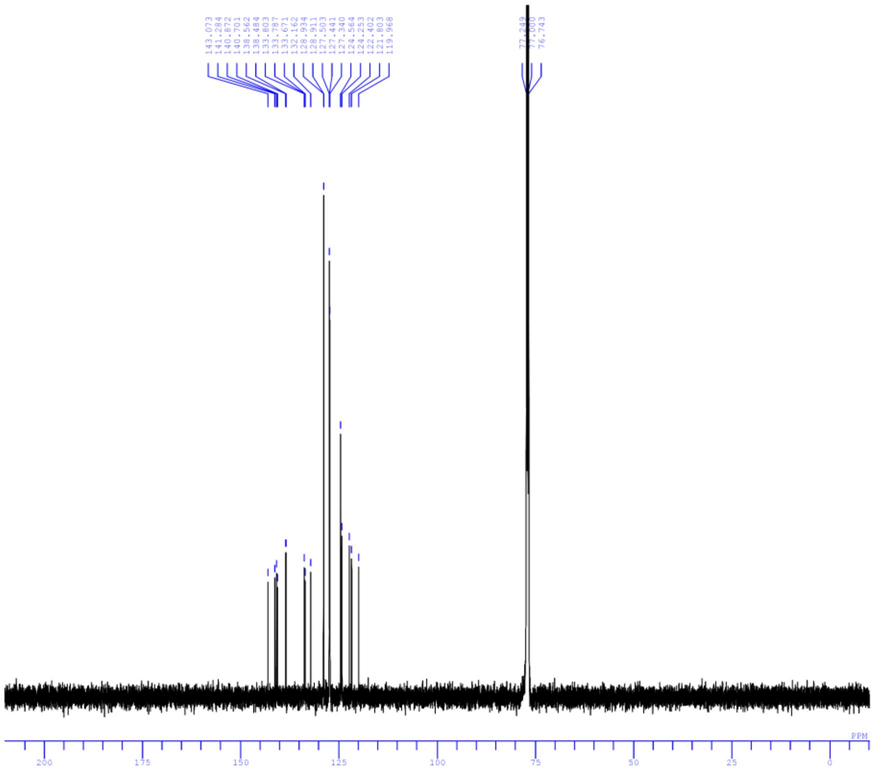

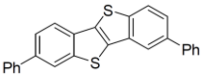

^{1}H NMR of 4eb (500 MHz, $CDCl_3$)

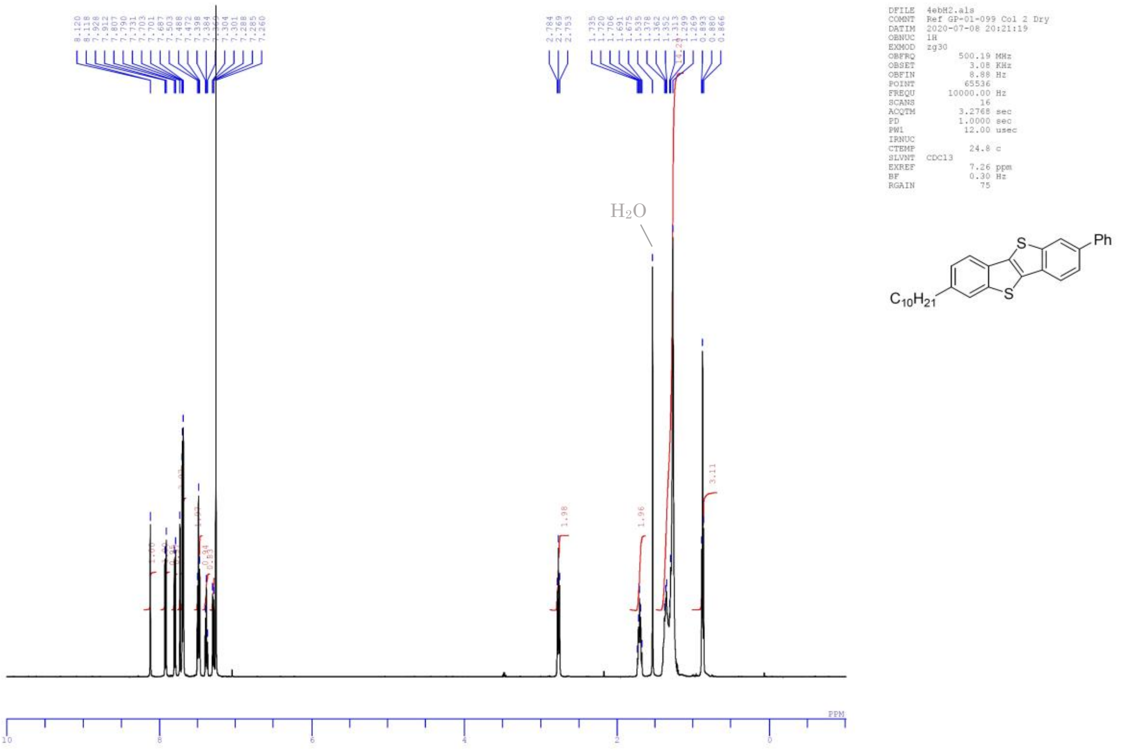

^{13}C NMR of 4eb (126 MHz, $CDCl_3$)

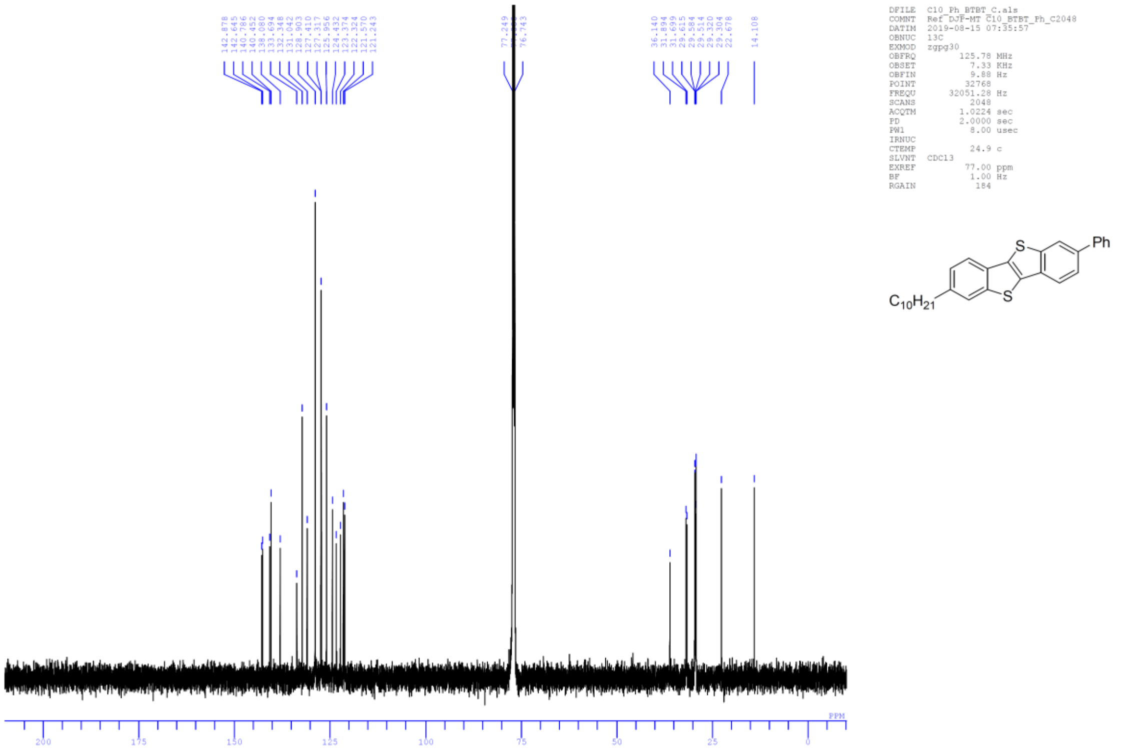

^{1}H NMR of 4ef (500 MHz, $CDCl_3$)

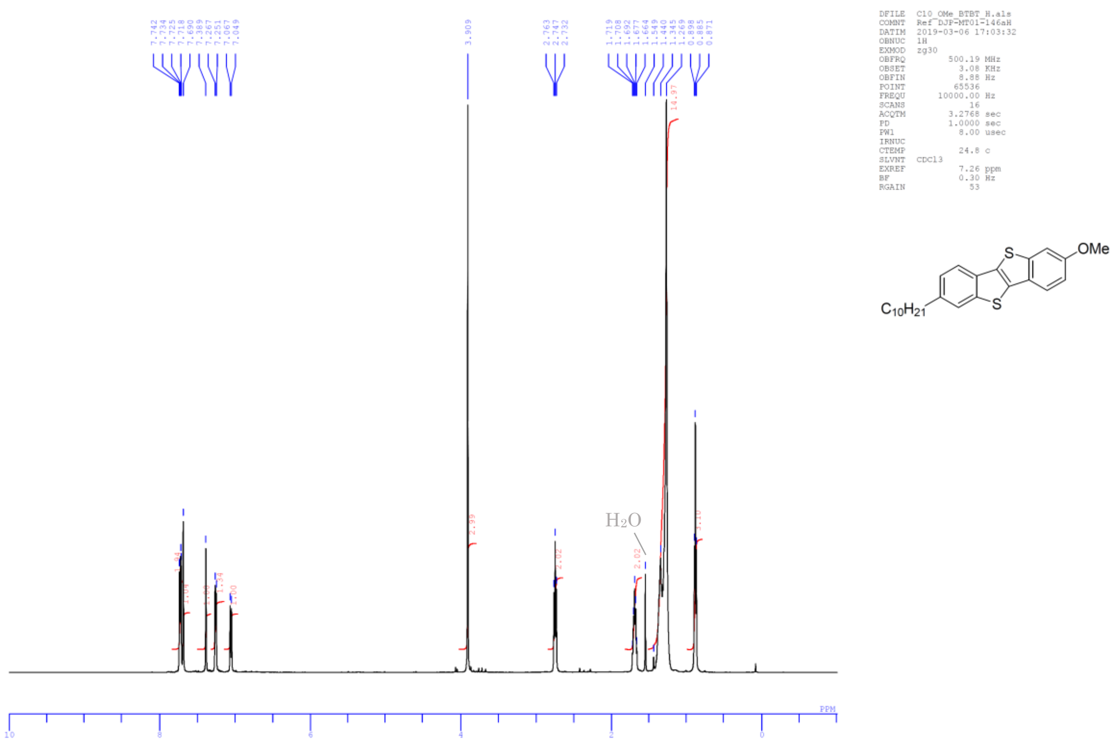

^{13}C NMR of 4ef (126 MHz, $CDCl_3$)

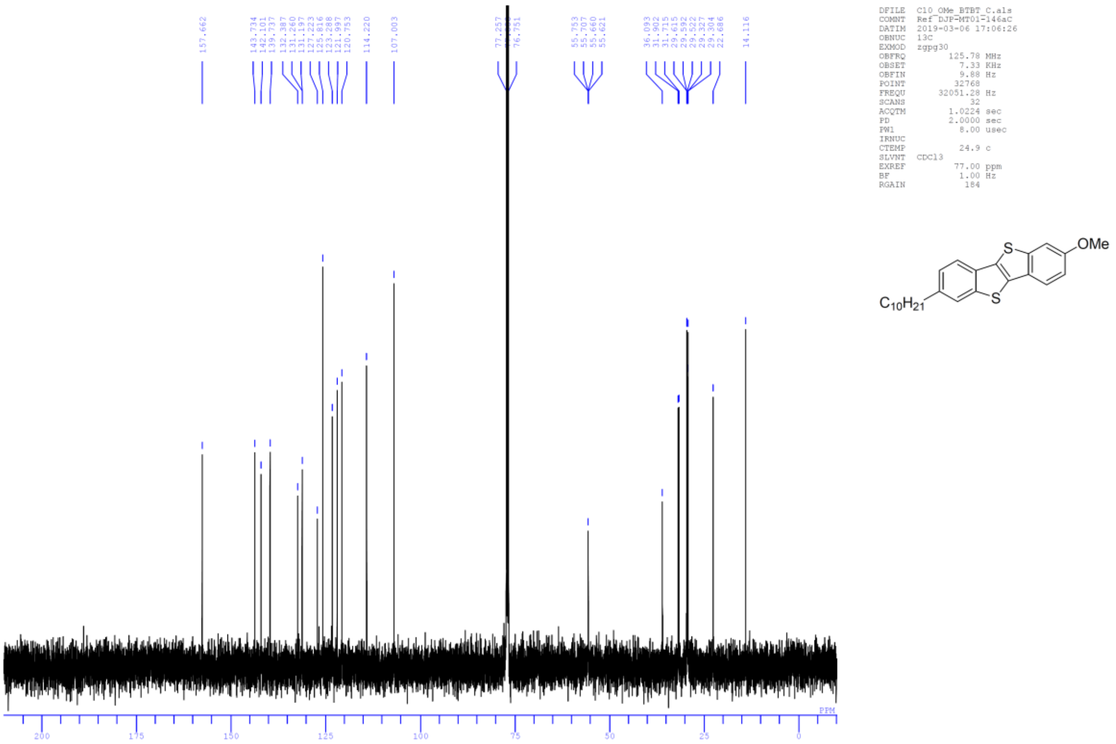

^{1}H NMR of 4eg (500 MHz, $CDCl_3$)

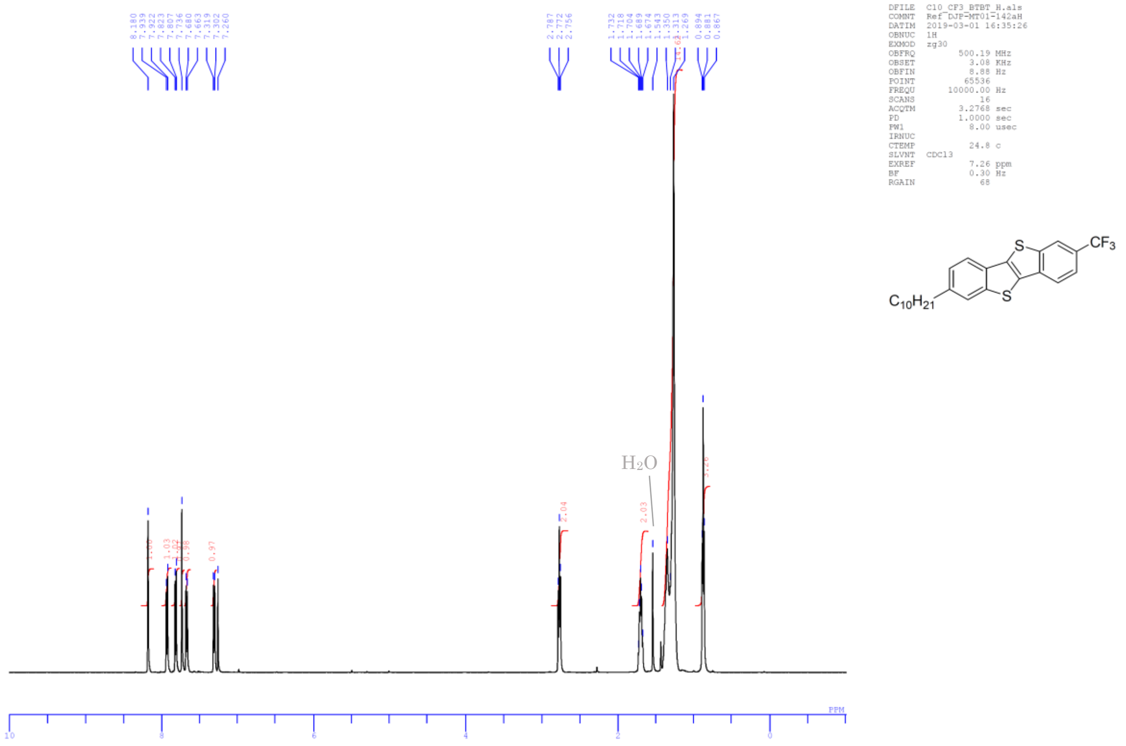

^{13}C NMR of 4eg (126 MHz, $CDCl_3$)

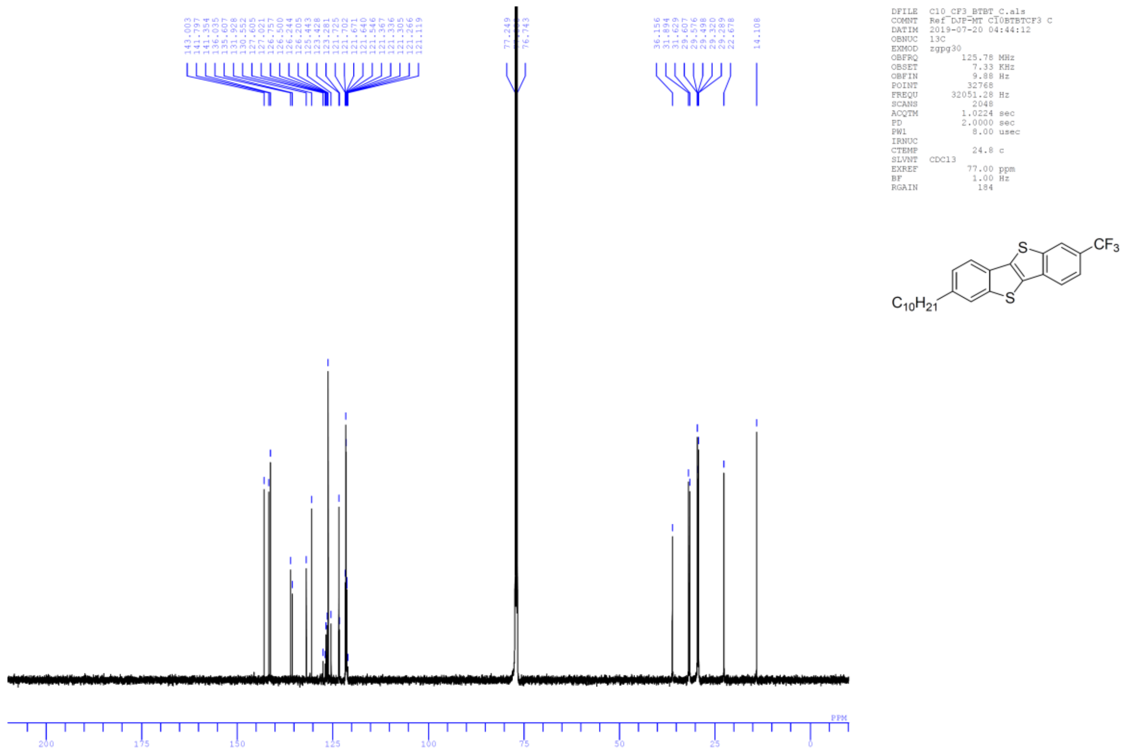

^{1}H NMR of 4eh (400 MHz, $CDCl_3$)

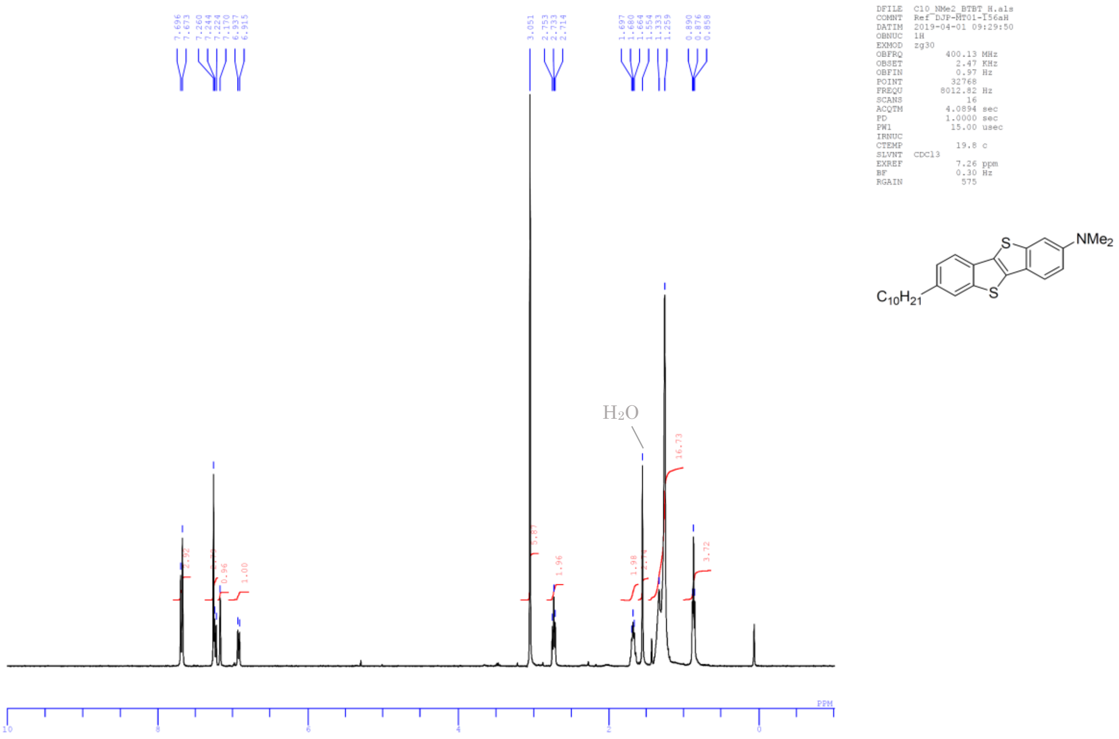

^{13}C NMR of 4eh (126 MHz, $CDCl_3$)

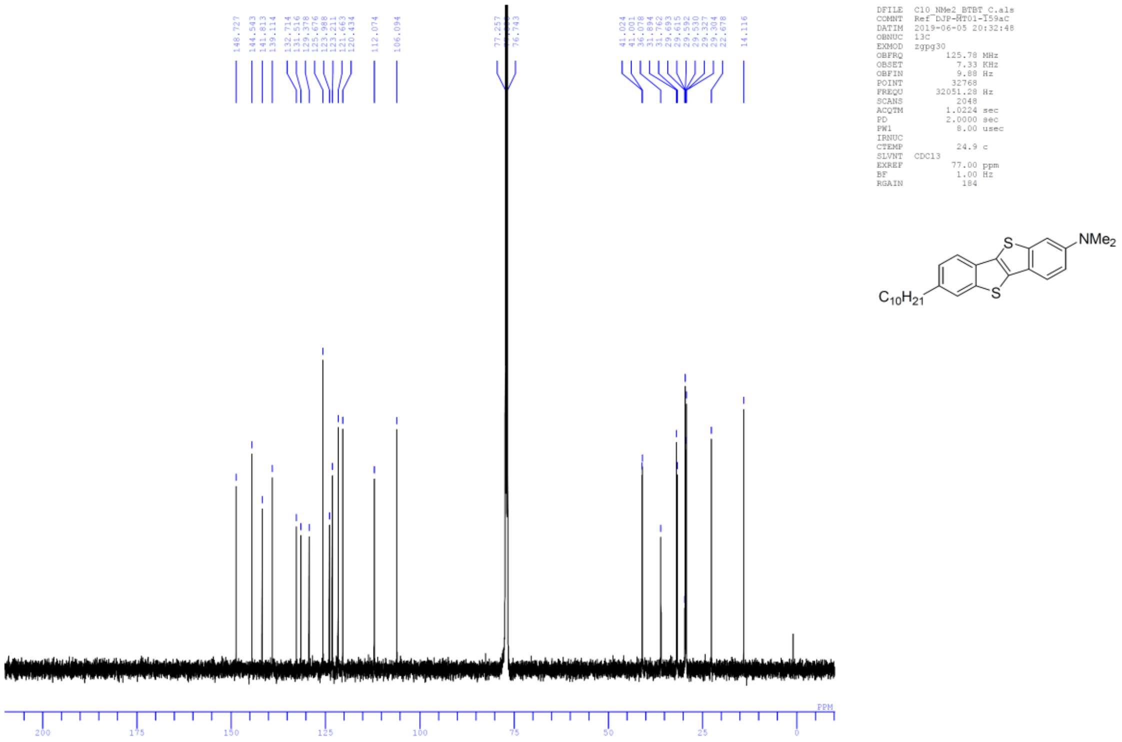

4. X-ray structure of 4ef

2-Decyl-7-methoxy[1]benzothieno[3,2-*b*][1]benzothiophene

CCDC 2103832

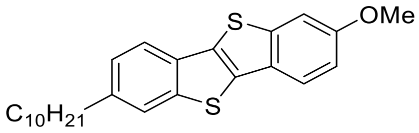

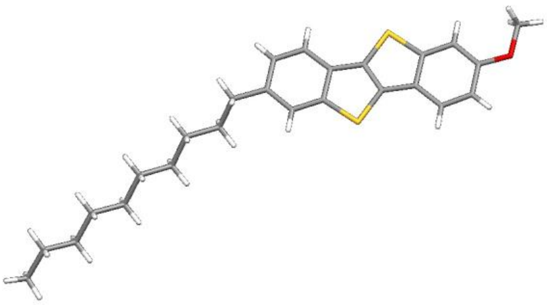

Bond Precision	C−C = 0.0124 Å	Wavelength	0.71073
Cell	a = 7.6759(3)	b = 48.446(2)	c = 5.9459(3)
	alpha = 90	beta = 90	gamma = 90
Temperature	240 K		
	Calculated		Reported
Volume	2211.08(17)		2211.08(17)
Space group	P c a 21		P c a 21
Hall group	P 2c -2ac		P 2c -2ac
Moiety formula	C25 H30 O S2		C25 H30 O S2
Sum formula	C25 H30 O S2		C25 H30 O S2
Mr	410.61		410.61
Dx,g cm-3	1.234		1.233
Z	4		4
Mu (mm-1)	0.254		0.254
F000	880.0		880.0
F000'	881.28		
k,k,lmax	10,69,8		10,63,7
Nref	6746[3685]		5052
Tmin,Tmax	0.929,0.970		0.499,1.000
Tmin'	0.913		
Correction method= # Reported T Limits: Tmin=0.499 Tmax=1.000 AbsCorr = MULTI-SCAN			
Data completeness	1.37/0.75	Theta(max)	30.484
R(reflections)	0.0900(4117)	wR2(reflections)	0.1988(5052)
S	1.101	Npar	255
