# Supplementary material for: Modular synthesis of unsymmetrical [1]benzothieno[3,2-b][1]benzothiophene molecular semiconductors for organic transistors
Source: Chem Sci. 2021 Nov 29;13(2):421–9. doi: 10.1039/d1sc05070b (PMC8730195; doi:10.1039/d1sc05070b)
Supplement: SC-013-D1SC05070B-s002 [file SC-013-D1SC05070B-s002.pdf]

Electronic Supplementary Material (ESI) for Chemical Science.
This journal is © The Royal Society of Chemistry 2021

Supporting Information

Part 2: Materials Section

Modular synthesis of unsymmetrical [1]benzothieno[3,2-b][1]benzothiophene (BTBT) molecular semiconductors for organic transistors

Masanori Tayu, Aiman Rahmanudin, Gregory J. P. Perry, Raja U. Khan, Daniel J. Tate, Raymundo Marcial-Hernandez, Yuan Shen, Ingo Dierking, Yurachat Janpatompong, Suphaluk Aphichatpanichakul, Adibah Zamhuri, Inigo Victoria-Yrezabal, Michael L. Turner* and David J. Procter*

Email: michael.turner@manchester.ac.uk, david.j.procter@manchester.ac.uk

Table of Contents

Part 1: Synthesis Section

1. **General synthesis procedures for BTBT derivatives** **S3**

   General Experimental **S3**

   General Procedure A: C-H Arylation of 3-Methoxybenzo[*b*]thiophene *S*-oxides **S4**

   General Procedure B: Synthesis of *S*-thiocarbamates **S4**

   General Procedure C: Hydrolysis and Cyclization to give BTBTs **S5**

2. **Experimental details for the synthesis of BTBT derivatives** **S6**

3. **^{1}H and ^{13}C NMR of BTBT derivatives** **S36**

4. **X-ray structure of 4ef** **S80**

Part 2: Materials Section

5. **Supporting figures and tables (as described in the main text)** **S83**

   Figure S1. Polarized optical microscopy of BTBT derivatives. **S83**

   Figure S2. Cyclic voltammograms of the BTBT derivatives. **S84**

   Figure S3. Transistor characteristics of 4eb semiconductor in bottom-gate/ top-contact configuration. **S85**

   Figure S4. Optical microscope images of the BTBT derivatives on SiO_2/ Si substrates. **S85**

   Figure S5-10. Representative transistor characteristics of pristine IDTBT and blends with BTBT derivatives. **S85-S90**

   Table S1. Summary of transistor parameters. **S91**

   Figure S11. Atomic force microscopy topography images of thin films. **S92**

6. **General experimental procedures** **S92**

   Synthesis **S92**

   Cyclic Voltammetry **S92**

   DFT Calculation **S93**

   DSC **S93**

   POM **S93**

   AFM **S94**

   OFET fabrication and characterization **S94-S95**

5. Supporting figures and tables as described in the main text

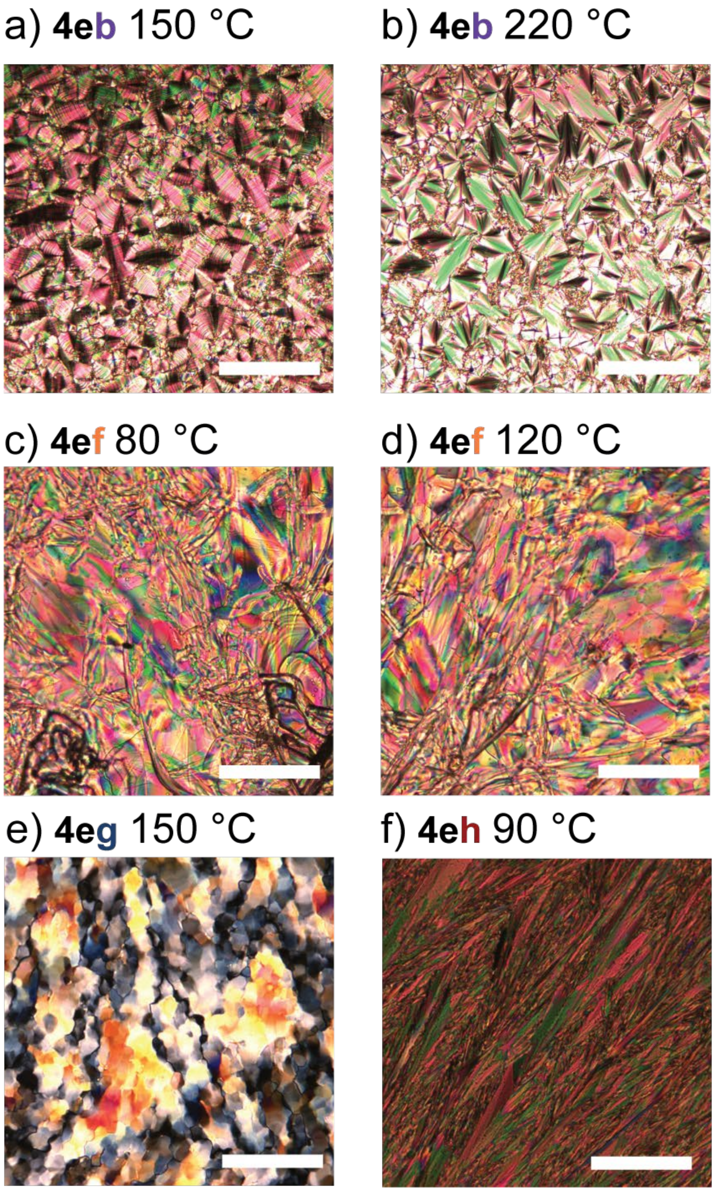

Figure S1. Polarized optical microscope images of 4eb (R = Ph) at a) 150°C in the SmE phase and b) 220 °C in the SmA phase; 4ef (R= OMe) at c) 80°C and d) 120°C; e) 4eg (R = CF_3) at 150 °C and f) 4eh (R = NMe_2) at 90°C. Scale bar (white) = 300 μm.

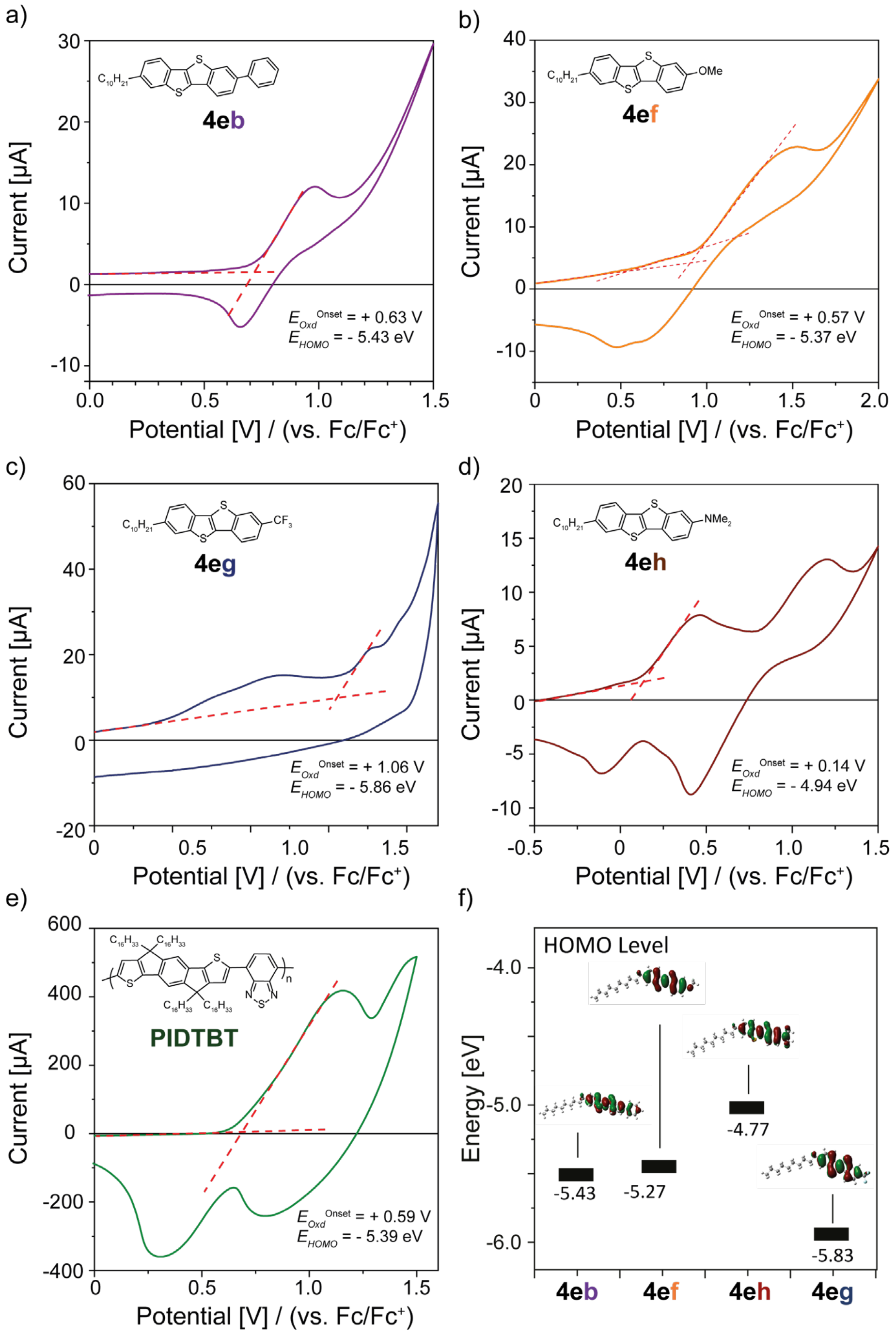

Figure S2. Cyclic voltammograms of the BTBT derivatives: a) 4eb (R = Ph); b) 4ef (R= OMe); c) 4eg (R = CF3); d) 4eh (R = NMe2);e) polymer semiconductor PIDTBT; f) DFT calculated HOMO levels including the molecular orbital diagram for each molecule.

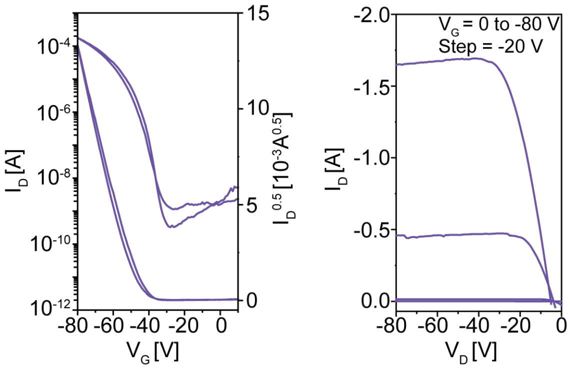

Figure S3. Representative transfer and output curves of transistors using **4eb** as the semiconductor in a bottom-gate/top-contact configuration. Thin film processing condition device fabrication were adapted from the previous report by H. Lino et.al.[1]

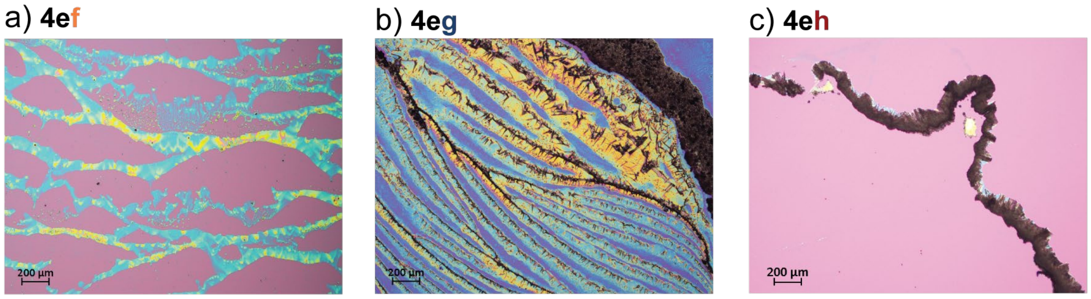

Figure S4. Optical microscope images of a) **4ef**, b) **4eg**, and c) **4eh**, *spin coated* from p-xylene (5 mg/mL) on SiO_2/Si substrates. Scale bar (black) = 200 µm.

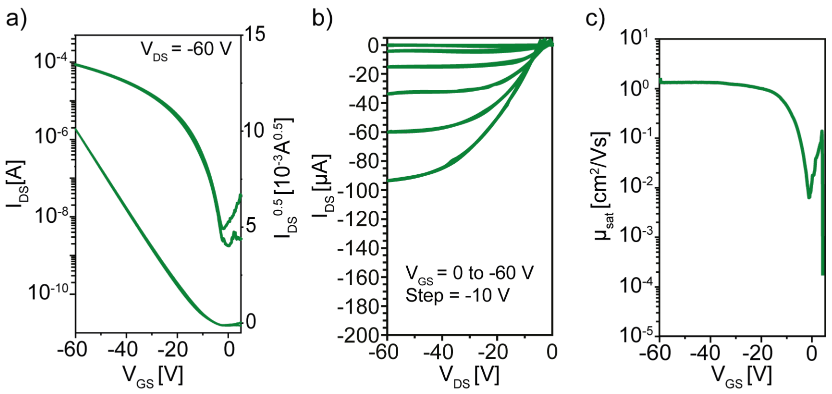

Figure S5. Representative a) transfer and b) output characteristics; and c) Mobility (μ_{sat}) applied in the saturation regime versus applied gate bias, V_G for pristine PIDTBT.

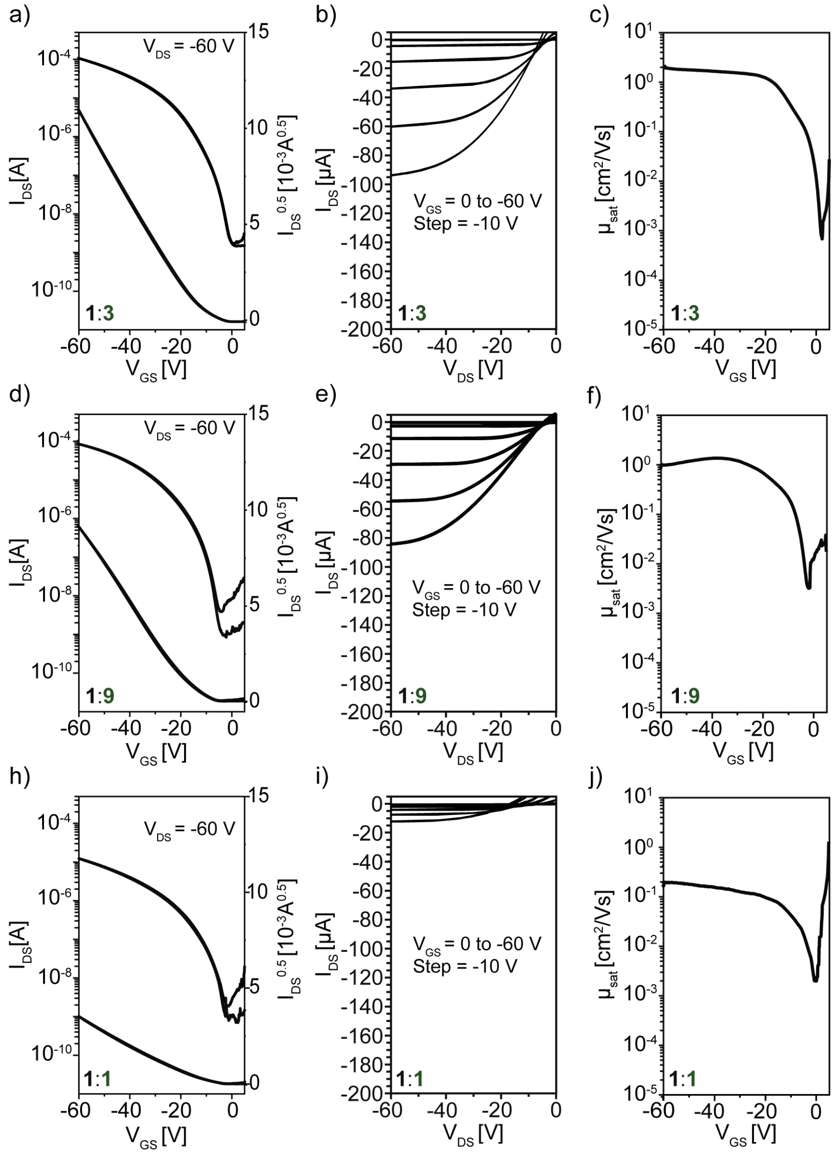

Figure S6. Representative transfer curves, output curves and mobility (μ_{sat}) versus applied gate bias of C8-BTBT:PIDTBT at blends ratio of a-c) 1:3, d-f) 1:9, and h-j) 1:1 ratio.

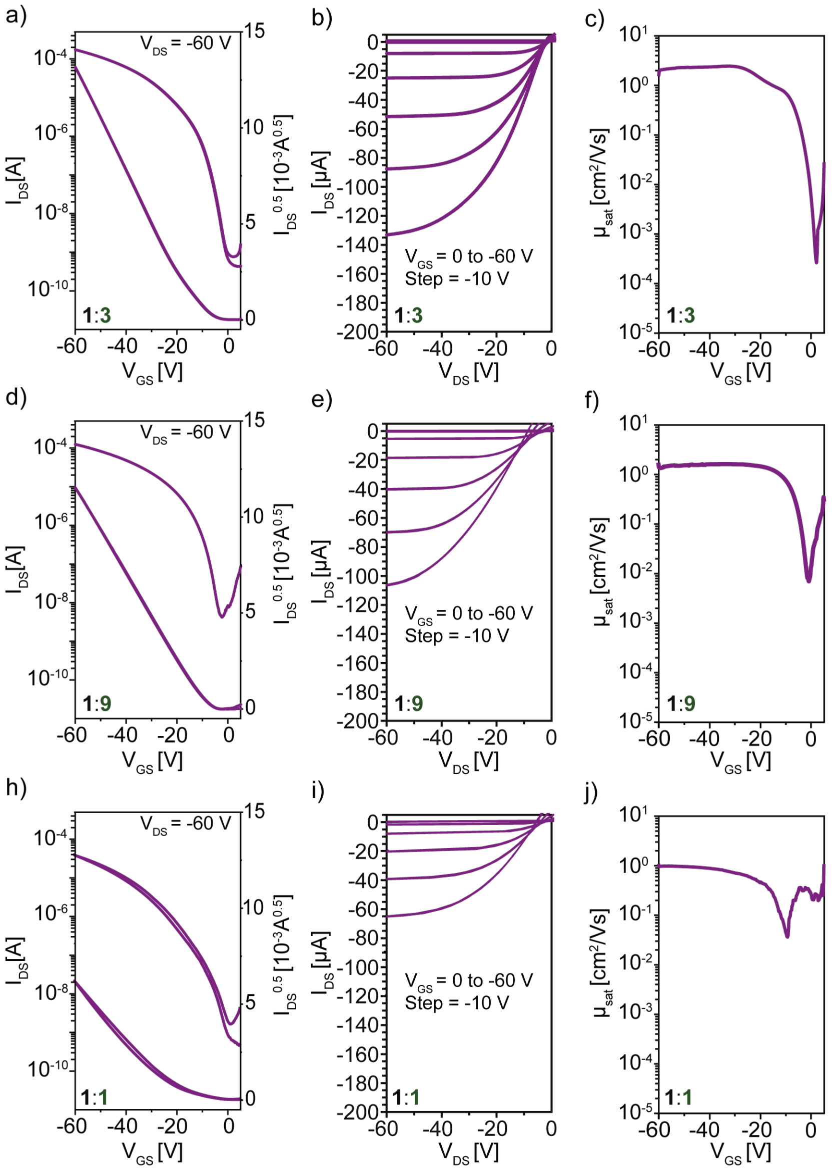

Figure S7. Representative transfer curves, output curves and mobility (μ_{sat}) versus applied gate bias of 4eb:PIDTBT at blends ratio of a-c) 1:3, d-f) 1:9, and h-j) 1:1 ratio.

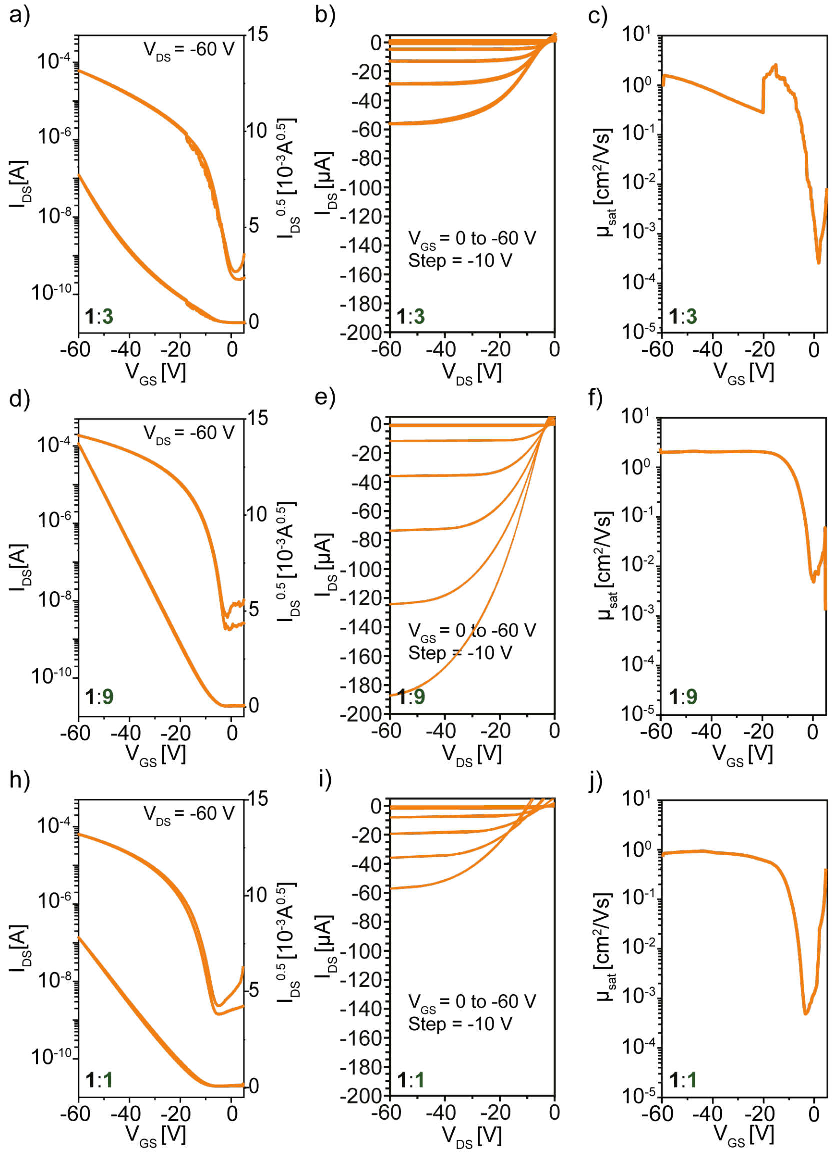

Figure S8. Representative transfer curves, output curves and mobility (μ_{sat}) versus applied gate bias of 4ef:PIDTBT at blends ratio of a-c) 1:3, d-f) 1:9, and h-j) 1:1 ratio.

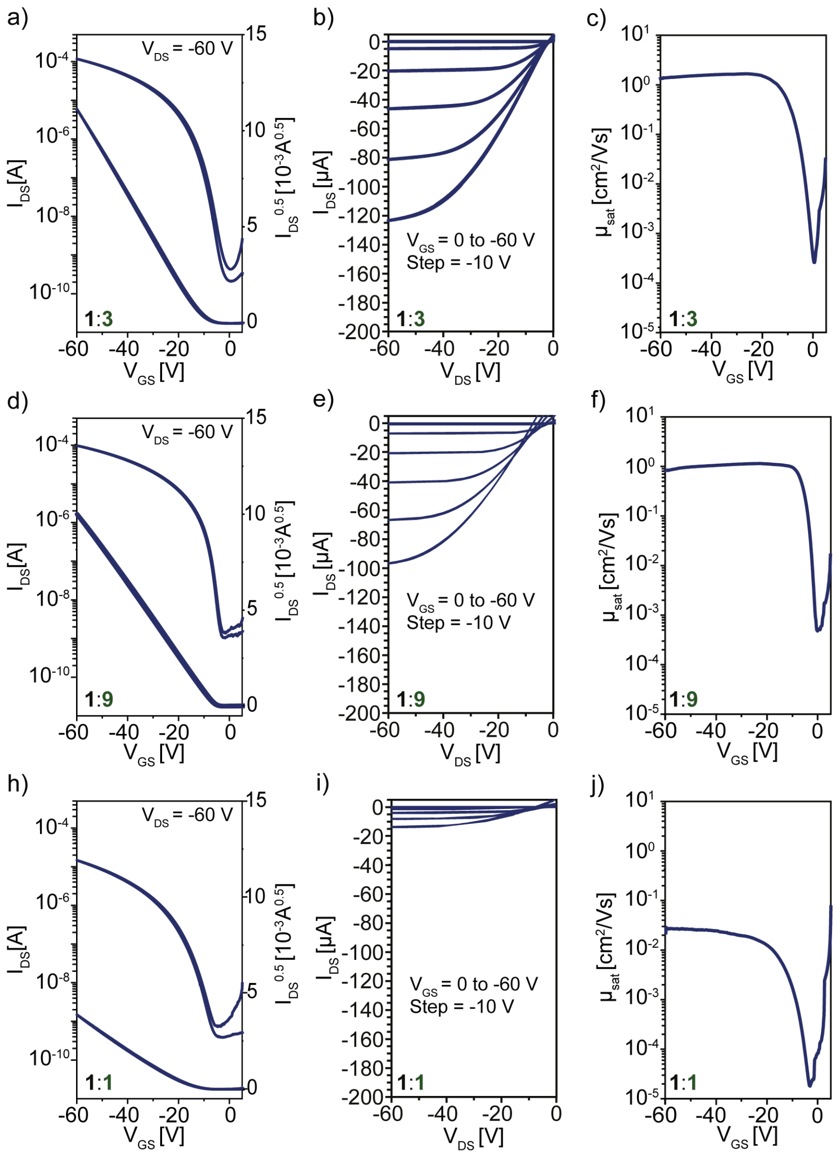

Figure S9. Representative transfer curves, output curves and mobility (μ_{sat}) versus applied gate bias of 4eg:PIDTBT at blends ratio of a-c) 1:3, d-f) 1:9, and h-j) 1:1 ratio.

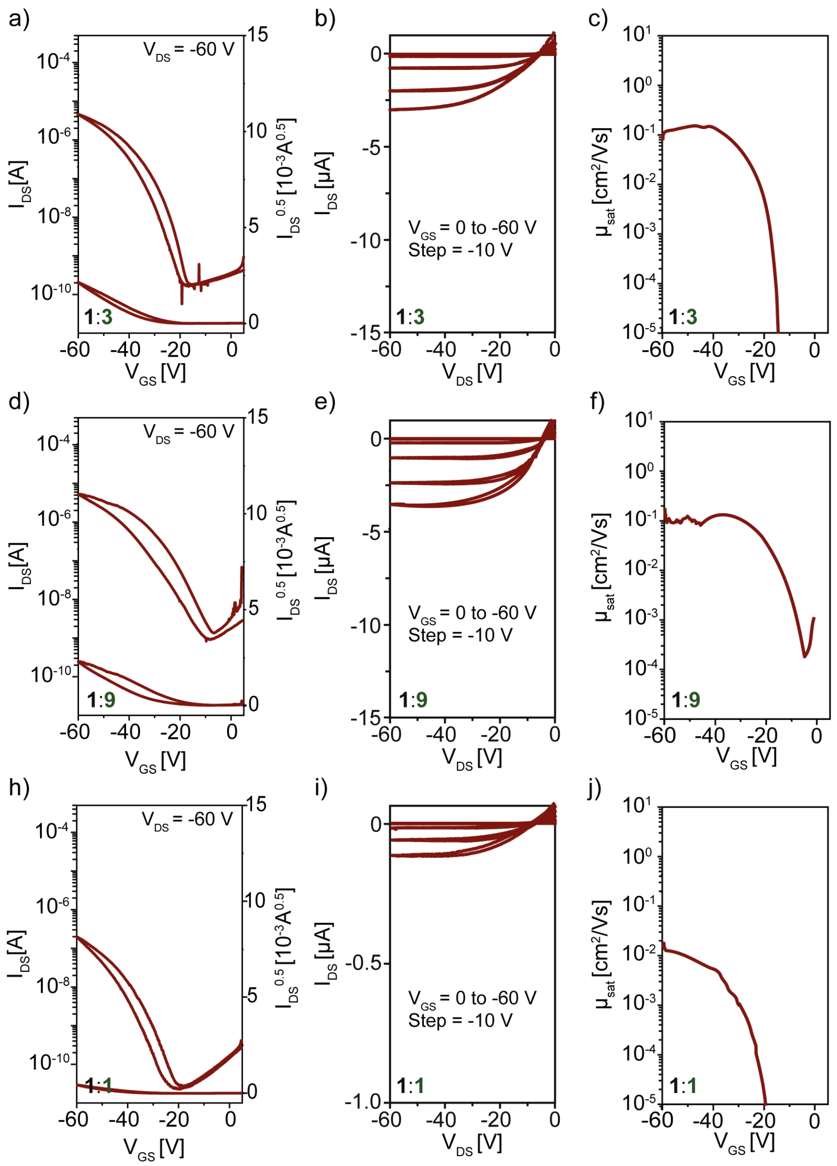

Figure S10. Representative transfer curves, output curves and mobility (μ_{sat}) versus applied gate bias of 4eh:PIDTBT at blends ratio of a-c) 1:3, d-f) 1:9, and h-j) 1:1 ratio.

Table 1. Summary of OFET Performance of Small-Molecule:Polymer Semiconductor Blends

OSC[a]	μ_{sat}[b] [$cm^2V^{-1}s^{-1}$]			V_{th}[b] [V]			I_{on}/I_{off}		
**PIDTBT**	1.02 ± 0.1			-3.3 ± 3.3			3.8×10^5		
**Blend Ratio**	**1:9**	**1:3**	**1:1**	**1:9**	**1:3**	**1:1**	**1:9**	**1:3**	**1:1**
**C_8-BTBT : PIDTBT**	0.96 ± 0.09	1.19 ± 0.3	0.11 ± 0.06	-6.2 ± 2.2	-10.8 ± 2.3	-15.7 ± 3.6	3.1×10^4	4.7×10^4	4.8×10^3
**4eb : PIDTBT**	1.38 ± 0.1	1.89 ± 0.3	0.82 ± 0.2	-5.1 ± 0.7	-9.2 ± 2.9	-13.8 ± 1.8	3.3×10^4	3.4×10^5	2.3×10^3
**4ef : PIDTBT**	1.87 ± 0.2	1.12 ± 0.2	0.66 ± 0.1	-6.7 ± 0.9	-12.9 ± 3.4	-13.8 ± 1.8	1.1×10^5	9.3×10^6	2.2×10^4
**4eg : PIDTBT**	1.00 ± 0.04	1.54 ± 0.05	0.15 ± 0.05	-7.0 ± 1.3	-9.0 ± 1.3	-15.8 ± 1.2	3.4×10^4	4.3×10^5	2.6×10^3
**4eh : PIDTBT**	0.08 ± 0.03	0.08 ± 0.04	0.005 ± 0.003	-12.9 ± 2.6	-30.3 ± 4.6	-36.6 ± 1.8	4.9×10^3	3.4×10^4	4.0×10^3

[a] See general experimental procedure for OSC solution preparation and device fabrication procedure.

[b] The OFET performance were averaged across 12 devices. μ and V_{th} were estimated in the saturation region using the gradual-channel approximation.

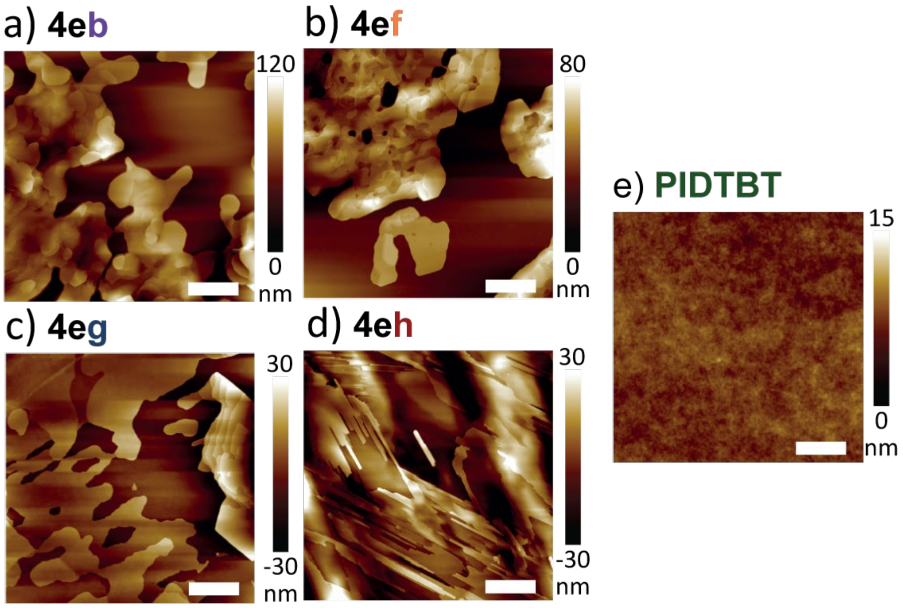

Figure S11. AFM topography images of thin films: a) 4eg; b) 4ef; c) 4eg; d) 4 eh; and e) PIDTBT. Scale bar (white) = 2 μm.

6. General experimental Procedure

OSC solution preparation: PIDTBT were made in accordance with previous procedures (W. Zhang, *et al.* J. Am. Chem. Soc. 2010, 132, 11437.). The OSC were dissolved in tetralin (10 mg/mL) for BTBT derivatives and in chlorobenzene (10 mg/mL) for PIDTBT separately, and left to stir overnight at 60 °C under argon atmosphere. The BTBT:PIDTBT solutions were blended in a 1:3, 1:9, and 1:1 ratio and left to stir for at least 30 minutes at 60 °C before processing into thin-films for the respective experiments.

Cyclic voltammetry measurements: The electrochemical CV were performed on a computer controlled CompactStat.h (Ivium Technologies ©) in a three-electrode configuration with a glassy carbon disk, Pt wire and Ag/Ag+ electrode as the working electrode, counter electrode, and reference electrode, respectively in a 0.1 M tetrabutylammonium hexafluorophosphate (NBu_4PF_6) acetonitrile solution as supporting electrolyte, at a scan rate of 50mV s^{-1}. The respective OSC solutions were drop casted on the glassy carbon disk and left to dry at room temperature before conducting the measurements. The potential of Ag/Ag+ reference electrode was internally

calibrated by using Ferrocene/Ferrocenium (Fc/Fc+) redox couple. The electrochemical energy levels were estimated by using the empirical formula: $E_{HOMO} = -(4.80 + E_{oxd}^{onset})$.

DFT Calculations: The electronic structures and properties of the molecules were studied using density functional theory (DFT) calculations, carried out with Gaussian09, and GaussView 5.0.8 molecular visualization program, employing single-point calculations with the B3LYP (Becke three-parameter hybrid correlation functional combined with Lee–Yang–Parr correlation functional). In the first step of the computational studies, geometry optimizations were carried out using 6-31G" basis set. After geometry optimization, electronic structure calculations were performed using a larger 6-311G(d,p) basis set.

Thermal Characterization: Differential Scanning Calorimetry (DSC) thermograms were measured with a PerkinElmer DSC8000 calibrated with indium and zinc, using a scanning rate of 20 °C min^{-1}. Samples were drop-casted from a known concentration of the OSC solution onto a glass substrate and left to evaporate slowly at room temperature under nitrogen atmosphere. The substrate were placed under vacuum to remove any residual solvents before transferring the solid powder into an aluminium pan for its thermal characterization.

Polarized optical microscopy: Sample Preparation and Measurements: The BTBT derivatives were placed on glass substrates and covered with cover slips. Both, the glass substrates and cover slips, are ultrasonically bathed for 20 minutes and subsequently baked at 100 °C for 10 minutes to evaporate the water. They are then spin-coated with glycerol to induce planar molecular alignment. The prepared samples are heated in a hot stage (LTSE350, Linkam) which is controlled by a temperature controller (TP 94, Linkam). Samples are then observed through a polarizing microscope (Leica OPTIPOL) with crossed polarizers. Images and movies are recorded

by a charge-coupled device camera (UI-3360CP-C-HQ, uEye Gigabit Ethernet).

Atomic Force Microscopy: The surface morphologies were measured with a Bruker Multimodal 8 atomic force microscope (AFM) in tapping mode using the SCANALYST-AIR cantilever with a silicon tip on a Nitride lever.

OFET fabrication and characterization procedure: Top-gate/bottom-contact device architecture was used with Corning glass as a carrier substrate. The glass substrates were cleaned in acetone, 10 wt% DECON 90 solution, DI water and isopropanol under sonication for 15 minutes each, followed by O_2 plasma treatment for 3 minutes before device fabrication. Cr/Au bottom electrodes were thermally evaporated under high vacuum (10^{-7} mbar) using a metal shadow mask with a patterned channel width = 1000 µm and length = 60 µm with a thickness of 3 nm/20 nm. The substrates were then treated with oxygen plasma for 3 minutes, and immediately treated with PFBT to adjust the work function of the Au electrodes. All OSC thin-films were processed in a nitrogen-filled glovebox. The respective OSC solutions were spin coated at 550 rpm for 10 s and 20 s at 1500 rpm to obtain the OSC thin-films which were then annealed at 120 °C for 5 minutes and left to cool to room temperature. AF2400 solution (25 mg mL^{-1}) was then spin-coated on top of the OSC thin film at 550 rpm for 20 s followed by 750 rpm for 30 s to obtain a dielectric layer of approximately 380 nm with an estimated aerial capacitance of C_i = 3.8 nF cm^{-2} measured at 100 kHz using an Agilent 4284A precision LCR meter. The substrates were annealed at 50 °C for 60 minutes and left to cool over night. Evaporation of the Al-gate electrode over a shadow mask leads to an OFET device ready for transistor characterization. The electrical properties of the OFETs were measured in the dark under ambient condition using an Agilent B1500 semiconductor parameter analyzer. The field-effect mobility (µ) was extracted using equation (1) in the saturation regime from the drain current versus gate voltage sweep:

$$I_{DS_{sat}} = C_i \mu_{sat} \frac{W}{2L} (V_{GS} - V_{th})^2 \qquad (1)$$

where I_{DS} is the drain current in the saturated regime, μ_{sat} is the field-effect mobility, C_i is the capacitance per unit area of the gate dielectric layer, V_{GS} and V_{th} are gate voltage and threshold voltage and W and L are channel width and length, respectively.
